# Crawling towards complex interactions: the impact of 6PPD-quinone and increased temperatures on the freshwater snail *Ampullaceana balthica*

**DOI:** 10.1007/s10646-026-03080-1

**Published:** 2026-04-24

**Authors:** Núria de Castro-Català, Catalina Lizama, Jordi Serra, Mira Čelić, Isabel Cadena, Mira Petrovic, Isabel Muñoz

**Affiliations:** 1 Department of Evolutionary Biology, Ecology and Environmental Sciences, Universitat de Barcelona, Av. Diagonal, 643, Barcelona, 08028 Spain; 2 Catalan Institute for Water Research (ICRA- CERCA), 17003 , Carrer Emili Grahit 101 Parc Científic I Tecnològic de la Universitat de Girona,, Girona, Spain; 3 Catalan Institution for Research and Advanced Studies (ICREA), Barcelona, Spain

**Keywords:** Tire wear contaminant, Wandering snail *Ampullaceana balthica*, Reproductive, physiological, Behavioural endpoints, Thermal stress

## Abstract

N-(1,3-Dimethylbutyl)-N′-phenyl-p-phenylenediamine-quinone (6PPD-quinone or 6PPD-Q) is an oxidation product of 6PPD, an antioxidant used in tires to prevent rubber degradation, that has been associated with high mortality in juvenile coho salmon at concentrations as low as 95 ngL⁻¹. While research has focused primarily on fish, the effects of 6PPD-Q on freshwater invertebrates remain limited. In this study, we assessed the toxicological impact of this contaminant on the freshwater snail *Ampullaceana balthica* over a 10-day experiment under two different temperature conditions. *A. balthica* was chosen because it is widely distributed in temperate and Mediterranean regions and is commonly used as a model organism in environmental toxicology studies. Although 6PPD-Q had a limited impact on embryonic hatching, adult snails experienced significant effects on reproduction, growth, and motility, with more pronounced impacts at higher temperatures. Specifically, 6PPD-Q reduced clutch and egg production, particularly during the first days of exposure. Elevated temperature increased reproduction, but its interaction with 6PPD-Q lowered the overall reproductive output. The combined stressors also impaired growth, motility and embryo development. These sublethal effects may lead to population declines and cascading impacts on freshwater community structure and ecosystem functioning, particularly under climate change scenarios. This highlights the urgent need for comprehensive risk assessments of emerging contaminants such as 6PPD-Q to better understand their ecological impacts.

**Supplementary Information:**

The online version contains supplementary material available at 10.1007/s10646-026-03080-1.

## Highlights

First evidence of sublethal 6PPD-Q effects on molluscs at environmental levels.

Repeated 6PPD-Q exposure impaired reproduction, growth, motility and embryo development in *Ampullaceana balthica*.

High temperatures increased egg production, but 6PPD-Q reduced reproductive output.

Growth rates declined with combined exposure to high temperatures and 6PPD-Q.

**Supplementary Information:**

The online version contains supplementary material available at 10.1007/s10646-026-03080-1.

## Introduction

Tire wear particles (TWP) are a growing source of pollution in urban freshwater ecosystems, released through tire abrasion and transported via stormwater runoff. These particles contain complex chemical mixtures that can persist in aquatic environments and disrupt biodiversity and ecosystem functioning by altering species composition, impairing reproduction, and destabilizing trophic interactions (Liu et al. 2024a, b; Song et al. 2025). Among the most concerning components of TWP is 6PPD (N-(1,3-dimethylbutyl)-N′-phenyl-1,4-phenylenediamine), a widely used rubber antioxidant that accounted for over 50% of global antioxidant consumption in 2017 (Li et al. 2023). During tire wear, 6PPD is released into the environment and undergoes oxidative transformation, particularly in the presence of ozone and UV radiation, forming 6PPD-quinone (6PPD-Q), a derivative that has been detected in stormwater runoff at concentrations up to 19 µgL⁻¹ and in surface waters during storm events at levels up to 3.5 µgL⁻¹ (Di et al. 2022; Johannessen et al. 2021; Seiwert et al. 2022; Tian et al. 2022).

At the same time, climate change is introducing additional stressors that may exacerbate these impacts such as frequent extreme weather events and increased thermal stress, particularly in semiarid regions where water availability is limited (IPCC, 2023; Terrado et al. 2014). Rising water temperatures, driven by global warming, can reduce the dilution capacity of aquatic systems by enhancing evaporation and lowering river discharge (Bolan et al. 2024). Warmer conditions also increase the solubility and bioavailability of pollutants such as 6PPD-Q, intensifying exposure for aquatic organisms (Holmstrup et al. 2010). These changes can lead to increased physiological stress, disrupting biological processes such as reproduction and embryo development (Hooper et al. 2013a; Seeland et al. 2012), and ultimately reducing the fitness and resilience of natural populations (Martínez-De León and Thakur 2024; Weiskopf et al. 2020). Moreover, chemical stressors may interact synergistically with increased temperatures associated with climate change, amplifying ecological impacts beyond those caused by each stressor individually (He et al. 2025; Zitoun et al. 2024). Notably, Holmstrup et al. (2010) found that in more than half of the studies reviewed, heat stress significantly increased pollutant toxicity. Understanding these combined effects is essential for assessing the risks posed by TWP-derived contaminants under future climate scenarios.

Research on 6PPD‑Q toxicity has so far been strongly centred on fish, largely because the compound was identified as the cause of acute die‑offs in coho salmon (*Oncorhynchus kisutch*), with lethal concentrations (LC50) as low as 95 ngL⁻¹ for juveniles (Tian et al. 2022). This has spurred research into its toxicity across other salmonid species. White spotted char (*Salvelinus leucomaenis)* is another sensitive species, with an LC50 of 0.51 µgL⁻¹, whereas rainbow trout (*Oncorhynchus mykiss*) and brook trout (*Salvelinus fontinalis*) show moderate sensitivity, with LC50 values between 0.59 and 1.96 µg L⁻¹. Alevins of lake trout (Salvelinus namaycush) exhibit a 45 day median lethal dose (LC50) of 0.39 µg L⁻¹ (Roberts et al. 2025). In contrast, the Arctic char (*Salvelinus alpinus*) and the nonsalmonid white sturgeon (*Acipenser transmontanus*) show greater tolerance, with LC50 values above 14.2 and 12.7 µg L⁻¹, respectively (Brinkmann et al. 2022; Hiki and Yamamoto 2022). Zebrafish (*Danio rerio*) and Japanese medaka (*Oryzias latipes*) show even greater tolerance, with LC50 values exceeding 40 µg L⁻¹ (Mayer et al. 2024). However, sublethal effects have been observed in zebrafish at lower concentrations. For instance, (Varshney et al. 2022) reported neurotoxic effects, including altered motor behaviour and bradycardia, after prolonged exposure to 10–20 µg L⁻¹ of 6PPD-Q. Similarly, Ricarte et al. (2023) demonstrated that short-term exposure to 2 µg L⁻¹ in zebrafish larvae leads to significant disruptions in essential behaviours, neurotransmitter profiles, circadian rhythms, and heart rates, highlighting the physiological disruptions that can occur at low concentrations even in the absence of mortality.

Beyond fish, information on 6PPD‑Q toxicity in aquatic invertebrates is comparatively scarce. Prosser et al. (2023) have found that 6PPD-Q does not cause significant mortality in four invertebrate species, the mayfly *Hexagenia* spp., the cladoceran *Daphnia magna*, the gastropod *Planorbella pilsbryi*, and the bivalve *Megalonaias nervosa*, at relatively low concentrations. However, the NOECs that they have reported, particularly for the gastropod *P. pilsbryi* (11.7 µg L⁻¹), do not eliminate the possibility of sublethal, long-term effects at concentrations that are still environmentally relevant, within the low µg L⁻¹ range. Despite the critical ecological roles of aquatic invertebrates, data on the sublethal toxicity of 6PPD-Q in these species remain limited. Recent studies have shown that prolonged exposure to 6PPD-Q at concentrations ranging from 1 to 10 µg L⁻¹ inhibits lifespan and induces multisystem toxic responses in *Caenorhabditis elegans* (Hua et al. 2024) and that *Daphnia pulex* experiences significant growth inhibition at 10 µg L⁻¹ (Shi et al., 2024).

In primary producers, such as the green algae *Chlorella vulgaris*, 6PPD-Q caused growth stimulation at concentrations ranging from 50 to 200 µg L⁻¹ but inhibited growth at higher concentrations (400 µg L⁻¹). Additionally, *C. vulgaris* experienced increased oxidative stress, affecting cell permeability and mitochondrial membrane potential stability (Liu et al. 2024a, b). These findings also suggest that the sublethal effects of 6PPD-Q could have broader ecological implications at low environmentally relevant concentrations.

Despite their ecological importance as grazers, bioindicators, and key components of benthic food webs, freshwater gastropods remain largely untested. These snails are important primary consumers in freshwater ecosystems and are particularly vulnerable to pollution because of their low motility. Unlike more mobile species such as fish, snails cannot escape from contaminated environments, increasing their susceptibility to prolonged exposure to pollutants (Baroudi et al. 2020). Freshwater snails feed primarily on biofilm that grow on submerged surfaces like stones or cobbles (Hladyz et al. 2011). This feeding behaviour exposes them to sunlight and, consequently, to increases in temperature, making them particularly vulnerable under warming conditions. Our study aimed to assess the impact of 6PPD-Q on the gastropod *Ampullaceana balthica* (Linnaeus, 1758) in this context. We hypothesized that chronic exposure to environmentally relevant concentrations of 6PPD-Q would lead to sublethal effects on the snails, with these impacts becoming more pronounced and potentially lethal at higher temperatures.

To assess these toxicological impacts, we exposed the snails and their offspring to environmentally relevant levels of 6PPD-Q at temperatures of 15 °C and 20 °C. With this research, we would like to contribute to a more accurate assessment of the risks that 6PPD-Q poses to freshwater invertebrates under future warming scenarios.

## Materials and methods

### Experiment setup

**Fig. 1:**
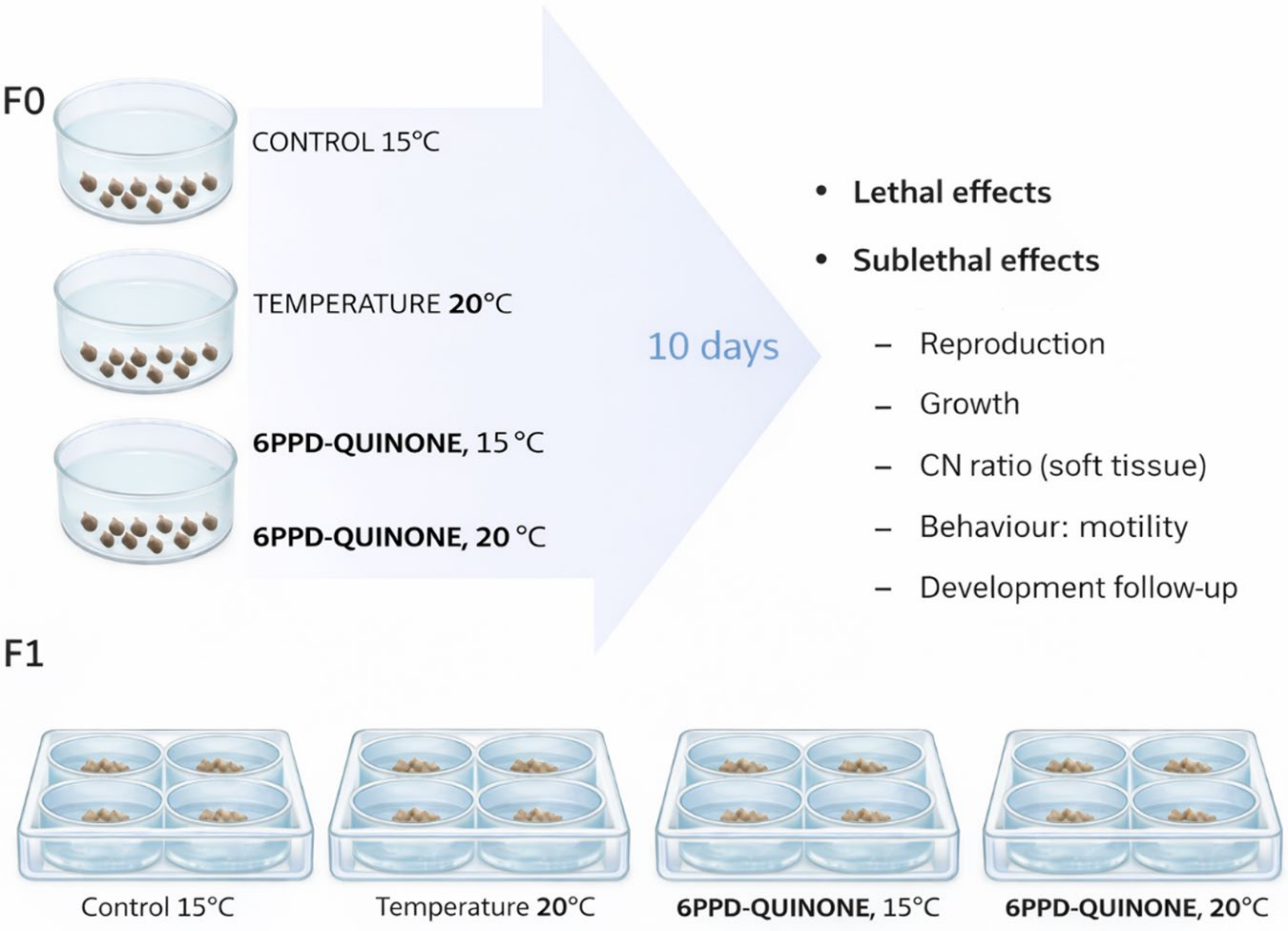
Experiment design and endpoints analysed

*A. balthica* snails, a simultaneous hermaphroditic freshwater gastropod species, were collected in late spring from the headwaters of the Ter River (42° 15’ 36’’, 2° 21’ 55’’). They were acclimated in 4 aquariums filled with 7 L of dechlorinated water at 15 °C and provided with constant aeration for 4 days prior to the experiment. After acclimation, they were exposed for 10 days in 5-L glass microcosms (26 cm diameter, 12 cm height) to 6PPD-Q at two different temperatures: 15 °C and 20 °C. The lower temperature of 15 °C falls within the typical temperature range recorded at the collection site during late spring. In contrast, 20 °C represents an upper-range temperature that can be reached during warm summer periods and may impose additional physiological stress if exposure is prolonged. This design allowed us to evaluate contaminant effects under both environmentally realistic and potentially stressful thermal conditions. All microcosms were first prepared at 15 °C, and then gradually adjusted by distributing them into two climate-controlled chambers, one maintained at 15 °C and the other gradually increased to 20 °C. The microcosms were maintained with calcium carbonate stones, constant aeration, and a 12 h:12 h light-dark cycle under controlled conditions. Each treatment group consisted of three replicates (microcosms), with 13 snails per replicate. The experiment lasted 10 days, and the analysed endpoints included mortality, growth, mobility, reproduction, embryonic development, and the CN ratio of the soft bodies (Fig. 1).

Snails were exposed to a nominal 6PPD-Q concentration of 10 µg L⁻¹, chosen to reflect environmentally relevant levels detected in surface waters during storm events (Cadena-Aizaga et al. 2025; Johannessen et al. 2021; Tian et al. 2022). The stock solution was prepared in absolute ethanol (final solvent concentration 0.01% v/v in exposure media) and diluted with dechlorinated water to achieve the target exposure concentration. The solution was thoroughly mixed before adult snails or embryos were introduced. The solvent was added to all other microcosms at the same final concentration to control for any potential solvent-related effects. A media renewal protocol was implemented, in which dechlorinated water (with or without the contaminant) was replaced every three days. This approach reflects real-world environmental conditions, particularly stormwater-driven runoff events that episodically introduce tire-derived contaminants, including 6PPDQ, into freshwater ecosystems (Cojoc et al. 2024; Johannessen et al. 2021; Tian et al. 2022). Such events generate transient concentration peaks rather than constant exposure, supporting the ecological relevance of pulsed exposure designs.

At the beginning of the experiment and after each renewal, 1 mg/snail of fish food (Tetramin^®^) was provided, following the recommendations of (Zimmer et al. 2012). The physical and chemical characteristics of the water were held constant throughout the experiment. Ammonia (NH₃/NH₄⁺) concentrations were monitored regularly using a commercial salicylate-based colorimetric test kit (API^®^ Ammonia Test Kit; detection range 0–8 mg L⁻¹), following the manufacturer’s instructions. Snail mortality was checked daily by gently touching each snail with the tip of a plastic pipette. If a snail showed no movement or response, such as retracting into its shell, it was considered dead. The number of dead snails was recorded each day, and any dead snails were promptly removed from the microcosms after being confirmed to be unresponsive. This method ensures accurate mortality tracking while preventing contamination from decomposing individuals.

### Reproduction and development follow-up

Clutches of *A. balthica* snails were systematically counted and collected every 3–4 days (on days 2, 4, 7, and 10), prior to water renewal. Immediately after collection, clutches were individually transferred to 6-well plates containing approximately 3 mL of exposure medium per well, under the same treatment and temperature conditions as their parental microcosm, thereby ensuring continuous second-generation (F1) exposure. The endpoints included the total number of clutches produced by the parental generation, the number of eggs per clutch, the number of embryos that successfully hatched, and the number of non-developed embryos, defined as those where development had stopped at the morula or gastrula stage.

### Growth and CN ratio analysis

At the end of the experiment, all snails were dried at 60 °C, weighed (dry weight), and their shells were measured under a stereomicroscope equipped with an ocular micrometer to calculate growth rates (Sampelayo et al. 1990). The carbon and nitrogen contents of the dried soft body samples were analysed via a Carlo Erba CN 1500 Analyzer (CCiT of the Universitat de Barcelona).

### Motility assessment

On Day 10, three snails from control and each treatment group were individually placed in Petri dishes to assess their movement. Each snail was video recorded for 15 min to capture their behaviour and mobility patterns. The footage from minute 2 (after acclimation) to minute 8 of each video was selected for analysis using the AnimApp application (Rao et al. 2019). This 2–8‑minute window excluded the initial acclimation period and, based on preliminary trials, provided a stable and comparable interval for reliable locomotor measurements.

### Statistical analyses

Generalized linear mixed-effects models (GLMMs), linear mixed-effects models (LMMs) and linear models (LM) were used to evaluate the effects of 6PPD-Q exposure (Treatment), temperature, time (Day), and their interactions on the different response variables. Experimental unit (microcosm) was included as a random intercept to account for non-independence among individuals within the same microcosm.

Count data were analysed using Poisson or negative binomial GLMMs with a log link (mortality counts, number of clutches, eggs per clutch). Proportional data (non-hatched and non-developed embryos) were analysed using binomial or beta-binomial GLMMs with a logit link to account for extra-binomial variation when present. Continuous response variables (growth rate, reproductive fitness, C:N ratio, and motility) were analysed using Gaussian LMMs. In this case, the data was log-transformed when necessary to meet the normality and homoscedasticity assumptions. GLMM fixed effects are reported as Wald z-tests, whereas LMM/LM fixed effects are reported as t-tests with Satterthwaite degrees of freedom.

When modelling rate data, offsets were included to account for exposure time and the number of individuals. The offsets were log(days) in mortality models and log (number of snails alive) in clutch-production models.

Model selection followed an information-theoretic approach based on AICc. Candidate models were generated from the global model using model dredging, and models with ΔAICc ≤ 2 were retained (Hobbs and Hilborn 2006; Johnson and Omland 2004). When multiple competing models were supported, model averaging was performed and parameter estimates were extracted from the averaged model. When more than one model fell within the ΔAICc ≤ 2 range, parameter estimates and test statistics were obtained from model averaging; otherwise, results correspond to the single best‑supported model.

Model assumptions were evaluated using residual diagnostics including tests for overdispersion, zero-inflation, and deviation from expected residual distributions (Bolker et al. 2009). For Gaussian models, diagnostic plots were inspected for normality and homoscedasticity of residuals.

Marginal and conditional R² values were calculated to quantify variance explained by fixed effects alone and by the full model (Nakagawa and Schielzeth 2013).

The final selected models are provided in R code and results for regression model selection are provided in Text S1.

All the statistical analyses were conducted in R v4.3.2 (R Core Team, 2023), using the packages MASS, car, lme4, glmmTMB, MuMIn, AICcmodavg, lmtest, DHARMa and ggplot2. Statistical significance was determined using a conventional α = 0.05 threshold.

### Chemical analysis of water samples

#### Compound preparation and sample handling

The compound 2-((4-methylpentan-2-yl)amino)-5-(phenylamino)cyclohexa-2,5-diene-1,4-dione (6PPD-Q; purity 97.1%, lot 1341848) was obtained from Dr. Ehrenstorfer (LGC). Stock solutions (6 mg L⁻¹) were prepared by dissolving 6PPQ in absolute ethanol (final solvent 0.01% v/v), stored at 4 °C in the dark for up to 7 days, and subsequently kept at − 20 °C until analysis.

Exposure water samples were collected on Day 0 and Day 2 under 20 °C conditions, and immediately prior to water renewal on Days 3 and 7 under both 20 °C and 15 °C conditions. All samples were stored at − 20 °C, following recommended guidelines. For direct analysis, stock solutions were diluted to 200 µg L⁻¹ to prevent precipitation or column overloading, and all samples were filtered through 0.45 μm PVDF membranes prior to LC analysis.

#### Sample preparation, extraction, and degradation experiment

Water samples (100 mL) were extracted in triplicate following the methodology described in detail elsewhere (Gago-Ferrero et al. 2015). Briefly, samples were sequentially filtered through glass fiber (0.7 μm) and PVDF (0.45 μm) membranes to remove particulates and were adjusted to pH 6.5–7.0 using 1.5 M ammonia or formic acid and spiked with 50 µL internal standard solution (0.5 µg/mL). For recovery assessment one blank sample was spiked with 25 or 50 µL of a standard mixture (1 µg mL). Solid Phase Extraction was performed on Oasis HLB cartridges (200 mg, 6 mL) preconditioned with dichloromethane, methanol, and water. Samples were loaded at ~ 2 mL/min, rinsed with water, dried under air for 30 min, and eluted with methanol and dichloromethane at 1 mL/min, followed by 1 min high-vacuum drying. Eluates were evaporated under nitrogen and reconstituted in 0.25 mL methanol: water (1:1, v/v; ×400 enrichment) and filtered through 0.2 μm PVDF membranes when necessary.

For the degradation assessment, 1 L microcosms were spiked with 6PPD-Q (10 mg L⁻¹ stock prepared as above) and incubated at 15 °C and 20 °C for 48 h (*n* = 2). Samples were collected at 30 min and 48 h and immediately analyzed alongside exposure water using UHPLC coupled to Orbitrap high-resolution mass spectrometry (UHPLC-Orbitrap-HRMS).

#### Instrumental analysis

All samples were analyzed using an ultra-high-performance liquid chromatography (UHPLC) system coupled to a high-resolution Orbitrap Exploris 120 mass spectrometer (Thermo Fisher Scientific). UHPLC separation was achieved on a Cortecs C18 + column (2.1 × 100 mm, 2.7 μm) with a VanGuard cartridge (2.1 × 5 mm, 2.7 μm) using water (0.1% formic acid) and methanol (0.1% formic acid) as mobile phases. The Orbitrap Exploris 120 mass spectrometer (Thermo Fisher Scientific) operated in positive ESI mode, with data acquired using all-ion fragmentation (AIF), following the method described by Gago-Ferrero et al. (2020).

#### Quality control and method validation

Instrument stability and analytical performance were assessed using calibration curves prepared over nine concentration levels, injected at the beginning and end of each sequence. Method limits of detection (LOD) and quantification (LOQ) were determined following standard procedures. Recoveries were evaluated by spiking exposure water blanks at two concentration levels, and all samples were analyzed in triplicate to ensure reproducibility. Routine quality control checks were performed to verify measurement reliability.

## Results

### Stock solution stability and instrumental performance

The 6PPD-Q stock solutions were stable when stored at 4 °C for up to 7 days and at − 20 °C for extended storage. Instrumental performance and sequence QA/QC. Instrument performance was monitored across analytical batches using bracketing calibration curves (at the start and end of each sequence) and replicate injections. Chromatographic performance was stable, with retention times typically within ± 0.05 min, and accurate-mass measurements meeting the identification criterion (mass error < 2 ppm). Repeatability was verified by replicate injections, yielding peak-area RSD values below 5%. Calibration curves across the working range showed excellent linearity (R² > 0.99) and did not indicate measurable response drift between the beginning and end of the analytical sequence (Fig. S1 and S2). Although a dedicated stock-solution stability study was not performed, consistent instrument response in bracketing calibrations and inter-day injections indicates that the working solutions remained fit-for-purpose over the timescale of the analyses.

### Method validation and recovery

Recovery studies conducted on spiked exposure water blanks (100 and 200 µg L⁻¹) yielded recoveries of 85–95%, with RSD values below 6%, confirming method precision. Limits of detection and quantification were determined as 0.5 µg L⁻¹ and 5 µg L⁻¹, respectively. No significant matrix effects were observed, indicating that the SPE procedure and sample filtration effectively removed interfering substances from water microcosmos.

### Exposure water sample analysis

Total ammonia concentrations remained below 0.2 mg L⁻¹ throughout acclimation and exposure. 6PPD-Q concentrations in exposure water samples were consistently measurable using both direct analysis and SPE-prepared extracts. We observed that freeze–thawing can lead to partial precipitation of the compound. This behavior may result in reduced solubility and uneven distribution in the exposure medium, and is most likely attributable to the low solubility of solid 6PPD-Q and potential crystallization (Hu et al. 2023). To minimize this effect, all frozen samples were thawed gently and homogenized prior to analysis. Samples filtered through 0.45 μm PVDF membranes prior to UHPLC analysis showed sharp, symmetric peaks with high signal-to-noise ratios (Fig. S3). The preconcentration factor achieved through SPE (×400) allowed detection of low concentrations with high reproducibility. The measured concentration of the stock solution (nominal concentration of 6 mg L⁻¹) was 2.09 mg L⁻¹. The samples collected on Day 0 and Day 2, under 20 °C conditions, presented concentrations of 1.9 µg L⁻¹ and 0.8 µg L⁻¹, respectively. The samples collected prior to water renewal on Days 3 and 7 presented concentrations below the detection limit of 0.5 µg L⁻¹. This decline over time may be attributed to photodegradation of 6PPD-Q, as the compound is known to degrade upon exposure to sunlight or UV radiation (Redman et al. 2023).

### Degradation kinetics of 6PPD-Q under different temperature conditions

In the 48 h degradation experiment, 6PPD-Q exhibited temperature-dependent degradation. At 15 °C, the compound decreased by 57.5% ± 3.1% over 48 h, whereas at 20 °C, degradation was faster, with a reduction of 82.8% ± 7.9% over the same period (Fig. S.4). These results demonstrate that temperature significantly influences 6PPD-Q stability in aqueous environments.

### Analytical reliability

Overall, the UHPLC-Orbitrap-HRMS method provided sensitive, accurate, and reproducible quantification of 6PPD-Q in both direct and SPE-prepared water samples. Quality control measures, including triplicate analysis and internal standard monitoring, confirmed the robustness and reliability of the analytical workflow.

### Mortality

Daily mortality was analysed using generalized linear mixed models (GLMMs) with a Poisson error distribution and log link, including unit (microcosm) as a random effect. Model selection based on AICc and model averaging identified treatment and time as the most supported fixed effects, whereas temperature was not retained in the final averaged structure (Table S1A).

**Fig. 2:**
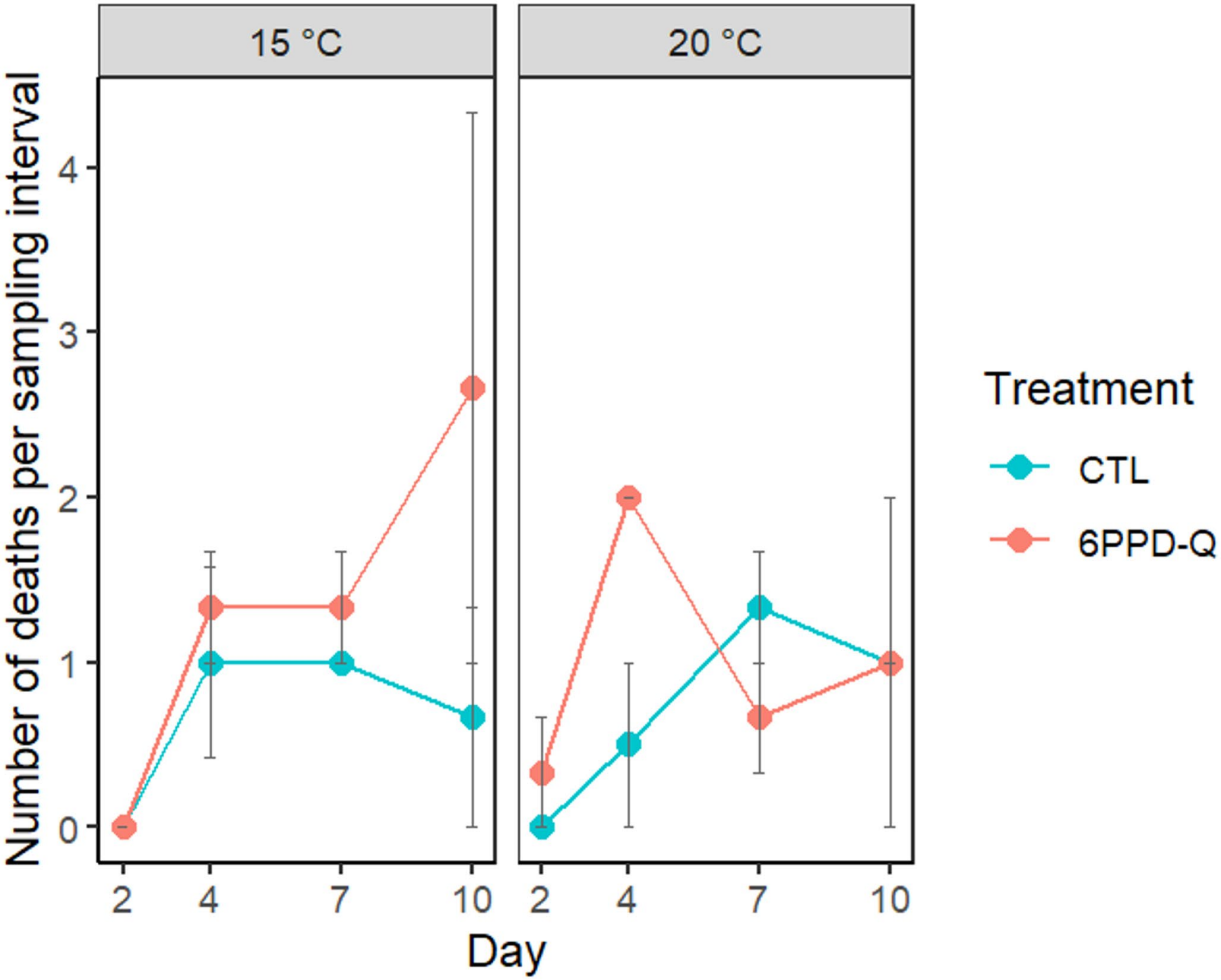
Mean number of deaths per sampling interval under control and 6PPD-Q treatments at 15 °C and 20 °C. Data are shown as mean ± s.e

The final selected Poisson GLMM did not detect any statistically significant effects of 6PPD-Q treatment (z = 1.60, *p* = 0.109) or time (z = 1.52, *p* = 0.129) on mortality (). Mean control mortality did not exceed 20% by the end of the experiment. (Fig. 2).

### Reproduction

**Fig. 3:**
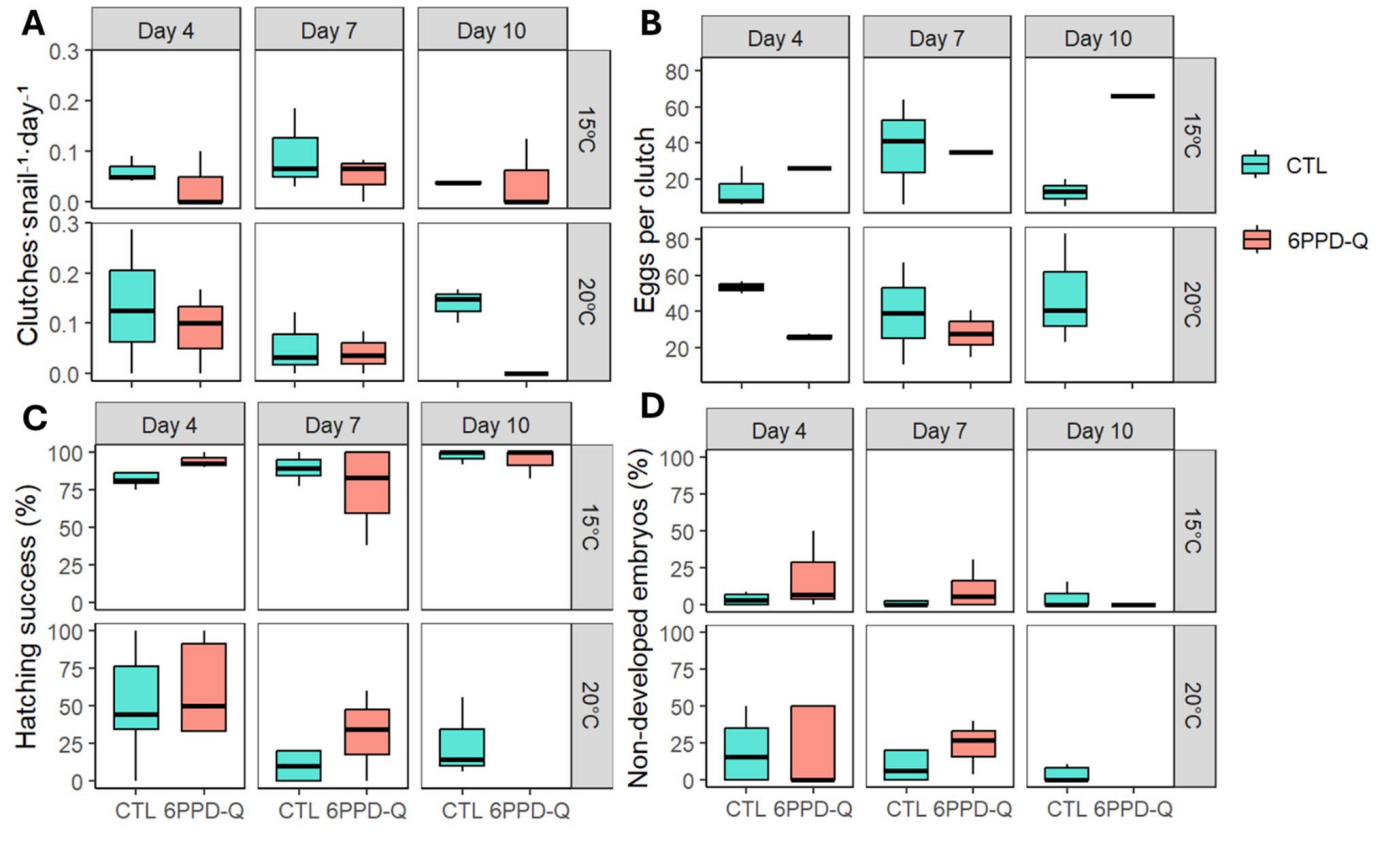
Reproductive and developmental endpoints plotted by treatment and temperature over time. (**A**) Number of clutches laid per snail alive per day. (**B**) Number of eggs per clutch. (**C**) Hatching success. (**D**) Percentage of non-developed embryos

For the number of clutches laid per snail alive during the experiment, a generalized linear model with a negative binomial distribution and log link was selected to account for overdispersion (Table S1B). The final model revealed a significant negative effect of 6PPD-Q in the number of clutches produced per snail alive (z = − 2.18, *p* = 0.029) (Fig. 3A).

**Fig. 4:**
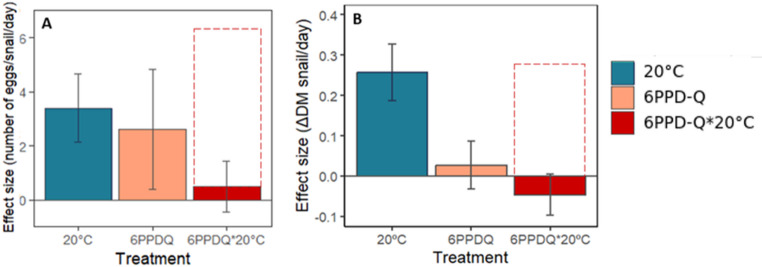
(**A**) Effect size of temperature and 6PPD-Q on the total number of eggs laid per snail alive per day; (**B**) Effect size of temperature and 6PPD-Q on the daily growth rate (measured as the change in dry mass) of the snails. Data are shown as mean ± s.e. The dashed line represents the expected additive effect under the Concentration Addition (CA) model. In both panels, the observed combined effects fall below this expectation, indicating non-additive interactions between temperature and 6PPD-Q

The number of eggs per clutch was analysed using generalized linear models with a negative binomial distribution and log link to account for overdispersion (Table S1C; ). Model selection based on AICc retained treatment (6PPD-Q), time, and their interaction in the final averaged structure, whereas temperature was not supported. The final model indicated a significant interaction between 6PPD-Q exposure and time (z = − 2.22, *p* = 0.026), showing that early clutches (days 0 to 7) laid under 6PPD-Q exposure contained fewer eggs compared to controls. However, this trend reversed in later clutches (days 7 to 10), resulting in a relative increase in egg number under contaminant exposure (Fig. 3B). No significant main effect of temperature was detected. Finally, we calculated the number of eggs laid per snail as a measure of reproductive fitness (i.e. the number of potential descendants per adult snail). Reproductive fitness was analysed using zero-inflated generalized linear mixed models with a Poisson distribution and log link, including unit (microcosm) as a random effect (Table S1D). Model selection identified temperature, time, and their interactions with 6PPD-Q as key predictors. The final model revealed a significant positive effect of temperature (z = 2.857, *p* = 0.004), indicating increased reproductive fitness at 20 °C. While the main effect of 6PPD-Q was not significant (*p* = 0.386), a significant negative interaction between temperature and 6PPD-Q was detected (z = − 2.076, *p* = 0.037), demonstrating that contaminant exposure reduced the positive thermal effect on reproduction. In addition, a significant Day × Treatment interaction (z = 2.887, *p* = 0.003) indicated that the temporal trajectory of reproductive output differed between treatments (Fig. 4A).

### Development

For hatching success, the final averaged beta-binomial GLMM indicated a significant effect of temperature (z = 5.24, *p* < 0.001), with a higher proportion of non-hatched embryos at 20 °C. A significant interaction between temperature and time was also detected (z = 2.96, *p* = 0.003), whereas no effects of 6PPD-Q exposure were supported (Table S1E; Fig. 3C).

For non-developed embryos, the final averaged binomial GLMM revealed a significant three-way interaction between treatment, temperature and time (z = 2.51, *p* = 0.012), indicating that the temporal pattern of developmental arrest depended jointly on contaminant exposure and temperature. No consistent independent main effects were detected (Table S1F; Fig. 3D).

### Growth

The final linear mixed-effects averaged model revealed a significant positive effect of temperature (t = 3.46, *p* < 0.001), indicating increased growth at 20 °C. However, a significant negative interaction between 6PPD-Q and temperature was detected (t = − 3.20, *p* = 0.0019). (Table S1G; Fig. 4B). This interaction indicates a negative effect, where the combined exposure to elevated temperature and 6PPD-Q led to reduced growth rates, below the control baseline.

### CN ratio

**Fig. 5:**
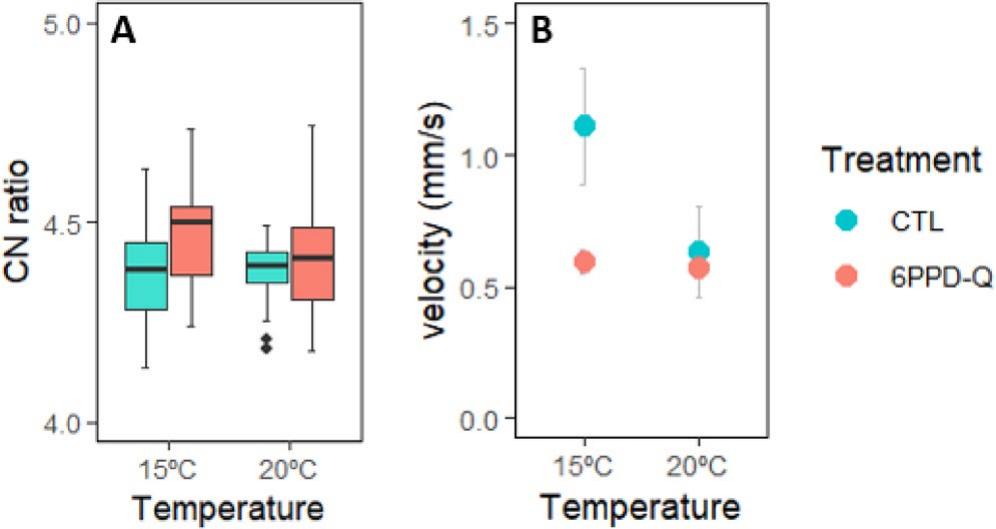
(**A**) CN ratio of the snail soft bodies plotted by the 6PPD-Q treatment and temperature; (**B**) Velocity of the snails plotted by the 6PPD-Q treatment and temperature. Data are shown as mean ± s.e

The final linear mixed-effects model selected (including unit as a random effect) showed no significant effects of 6PPD-Q (t = 1.84, *p* = 0.069) or temperature (t = 1.32, *p* = 0.19), indicating that elemental composition was not significantly altered after the 10 days of exposure. (Table S1H; Fig. 5A).

### Motility

The final linear model revealed significant negative effects of 6PPD-Q (t = − 2.57, *p* = 0.033) and Temperature (t = − 2.36, *p* = 0.046), indicating reduced velocity under contaminant exposure and at 20 °C. No significant interaction between treatment and temperature was detected (Table S1I; Fig. 5B).

## Discussion

This study reveals the toxicological impact of 6PPD-Q, an emerging contaminant linked to tire wear, on various life history traits of snails. As hypothesized, the sublethal effects observed on reproduction, development, growth, and motility reveal a broad spectrum of ecological risks associated with 6PPD-Q exposure at environmentally relevant concentrations, emphasizing the need for regulatory measures and safer alternatives. Our results also reveal that some of these effects became more pronounced at higher temperatures.

Regarding chemical exposure dynamics, the rapid decline in 6PPD-Q concentrations within each renewal cycle indicates that exposure occurred as repeated pulses rather than as a constant concentration. Degradation experiments confirmed the limited aqueous stability of the compound (Hu et al. 2023), with concentrations decreasing by approximately 57% at 15 °C and 83% at 20 °C within two days and falling below the LOD after each three‑day cycle. Consequently, the experimental design reproduced episodic exposure conditions similar to those occurring during stormwater‑driven runoff events, with each medium renewal reintroducing fresh pulses of 6PPD‑Q under climatic and microclimatic regimes characterized by intermittent rainfall and runoff. Although continuous flow-through systems would allow more stable exposure concentrations and facilitate toxicity threshold derivation (e.g., ECx values), pulsed exposure regimes may better reflect real-world contamination patterns for tire-derived contaminants. In natural situations, despite the tendency of 6PPD-Q to partition onto soil and organic matter due to its hydrophobic nature (log Kow = 4.3 ± 0.02) (Hu et al. 2023; Hua et al. 2024), a portion remains in the dissolved aqueous phase during storm events, generating repeated high-concentration exposures comparable to those simulated here.

These exposure dynamics clearly translated into measurable sublethal and long-term biological effects. Although mortality showed only a weak temporal trend and no treatment response, thus contributing little explanatory power, this absence of short‑term effects underscores the relevance of examining longer‑term or cumulative impacts. In line with this, the exposure to 10.45 µg L⁻¹ of 6PPD-Q had a significant negative impact on sublethal endpoints such as the reproductive output of the freshwater snail *Ampullaceana balthica*, specifically affecting both the number of clutches laid and the number of eggs per clutch. Compared with nonexposed snails, snails exposed to 6PPD-Q laid fewer clutches.

With respect to the number of eggs per clutch, the results revealed a more complex interaction. Initially, during the first week, 6PPD-Q exposure resulted in a lower number of eggs per clutch compared to nonexposed clutches. However, a significant interaction effect between 6PPD-Q and time was observed, with clutches laid later in the experiment (Days 8 to 10) in the contaminated aquaria resulting in an increase in the number of eggs per clutch. This temporal pattern may suggest a delayed compensatory response, where snails initially reduce egg production but later attempt to increase reproductive output as exposure continues. Similar responses have been observed in various organisms, where reproductive efforts are maximized following an initial period of stress to improve overall reproductive success (Minchella and Loverde 1981; Rollo and Hawryluk 1988). For example, Bi et al. (2025) observed that *Pomacea canaliculata* exposed to low concentrations of arsenite significantly increased egg production. This was interpreted as a hormetic response, where mild environmental stress stimulates reproductive output through physiological or endocrine mechanisms. Similarly, Liang et al. (2023) reported enhanced spawning and estradiol levels in *P. canaliculata* under low-level pollution, suggesting an adaptive strategy to ensure reproductive success under suboptimal conditions. Likewise, *Lymnaea stagnalis* exposed to cigarette butt leachate initially reduced clutch production, but reproductive output recovered during a post-exposure phase (Olah-Kovacs et al. 2025). These findings suggest that freshwater snails may exhibit plastic reproductive strategies that allow partial recovery from sublethal stress.

The analysis of overall reproductive fitness, measured as the number of eggs laid per snail per day, revealed a significant negative interaction between 6PPD-Q and temperature suggesting that the detrimental effects of the contaminant may be exacerbated at relatively high temperatures, potentially due to increased metabolic demands (Clarke and Fraser 2004; Cloyed et al. 2019; Scrine et al. 2017) or increased uptake of the contaminant (Guo et al. 2018; Heugens et al. 2001; Hooper et al. 2013b). While the temporary increase in egg production per clutch may reflect an adaptive reproductive response to stressful conditions, the overall reduction in reproductive fitness indicates that this compensatory strategy is insufficient.

The repeated pulse-recovery dynamic could explain the initial reduction in reproductive output, followed by a compensatory increase in egg production later in the experiment. However, despite this compensatory effort, the overall negative impact on reproductive fitness suggests that the snails were unable to fully recover from the stress induced by repetitive peak exposures. Over time, the exposure to 6PPD-Q reduces the reproductive capacity of snails, potentially threatening population sustainability in contaminated environments. This aligns with findings from other studies, where environmental stress or limited resources cause snails to reduce their reproductive efforts to survive. For example, in *Lymnaea stagnalis* reproduction was delayed under food limitation, diverting energy toward maintenance, reducing the number of eggs produced (Zonneveld and Kooijman 1993). Similar trade-offs between reproduction and survival have been observed in snails exposed to contaminants such as copper, which also reduced both reproductive output and growth (Gao et al. 2017; Khangarot and Das 2010; Real et al., 2024). Additionally, findings by (Wang and Liu 2023) show that under silver nanoparticle exposure, adult gastropods allocate more resources to combat oxidative stress rather than to growth or reproduction, further emphasizing the energy trade-offs under environmental stress.

In our study, growth rates were also negatively impacted by the contaminant, especially at relatively warm temperatures. Normally, higher temperatures promote growth by accelerating metabolic rates, but this positive effect was diminished by the contaminant. This suggests that 6PPD-Q may disrupt energy allocation, forcing snail detoxification over growth (Wang and Liu 2023). Motility was also significantly reduced in snails exposed to 6PPD-Q, with additional negative effects observed at relatively high temperatures. The reduction in motility could further decrease survival chances, as limited movement may impair foraging and escape from predators. The combined effects of 6PPD-Q and temperature on motility suggest that the contaminant induces physiological stress, limiting the capacity of the snails to perform essential behaviours. Reduced motility could further compound the negative effects on growth and reproduction, as limited movement restricts access to food and mates, ultimately diminishing the overall fitness of the population. The effects of 6PPD-Q at similar concentrations have also been documented in other invertebrates, with nematodes showing abnormal locomotion behaviours and neurodegeneration at 10 µg L⁻¹ (Hua et al. 2023), and *Daphnia pulex* exhibiting significant reductions in growth rates at exposure levels of 0.1 and 10 µg L⁻¹ (Shi et al., 2024).

While 6PPD-Q clearly affected reproduction, its effects on embryonic development were less pronounced. Exposure to 6PPD‑Q increased the proportion of eggs that failed to initiate development (non‑developed), and this effect became more evident over time, particularly in the clutches laid between days 4 and 7 at 20 °C. In this treatment–temperature combination, developmental failure increased fastest in the presence of the chemical. In these same treatments no clutches were produced in the final interval (days 7 to 10), indicating that adult snails had already been affected by 6PPD‑Q and that the impairment was strong enough to disrupt reproduction before the experiment ended.

In contrast, for embryos that did initiate development but failed to hatch (non‑hatched), no effect of 6PPD‑Q was detected. Instead, hatching failure increased primarily as a function of time and, especially, temperature, independently of chemical exposure. At 20 °C, the decline in hatching success was pronounced in both treatments, consistent with a temperature‑driven effect rather than a contaminant‑driven one. This may be due to the protective features of the egg masses and eggshells. Initially, 6PPD-Q must penetrate the gelatinous matrix surrounding the egg masses, which consists of proteins and polysaccharides that can limit the entry of external contaminants (Benkendorff et al. 2001). The degree of protection offered by this matrix varies depending on factors such as the chemical’s size, polarity, and lipophilicity (Arman 2023). For example, snail eggs within the matrix showed reduced sensitivity to cadmium compared to isolated eggs (Liu et al. 2013), although some contaminants (e.g., nanoparticles) can agglomerate and adhere to egg masses (Musee et al. 2010).

Once it passes through the gelatinous matrix, 6PPD-Q needs to cross the egg capsule membrane itself. Studies have shown that molecules such as raffinose (504.42 g/mol) and polyethylene glycol (500–600 g/mol) can pass through this membrane (Beadle 1969). Given its relatively small molecular weight of 298.4 g/mol, 6PPD-Q likely has the ability to permeate the egg capsule as well. The limited effect on hatching is therefore consistent with partial protection by the matrix and capsule that restricts diffusion, while not excluding some permeation. Quantifying internal embryonic exposure will be necessary to resolve these pathways. Despite these barriers, the reduced number of eggs laid and overall reproductive impairment suggest that the long-term developmental success of the population could be compromised (Gao et al. 2017; Khangarot and Das 2010), warranting further work on persistent or delayed sublethal effects after hatching. In addition to the direct exposure from contaminated water during rainfall events, 6PPD-Q can interact with organic matter, such as leaf litter and biofilms, which aquatic organisms may ingest. Tire wear particles in the water can also be accidentally ingested, releasing 6PPD-Q into the digestive systems of these organisms (Arman 2023). This raises concerns about the potential for bioaccumulation of 6PPD-Q when contaminated food is consumed. Although few studies have examined the bioavailability of 6PPD-Q, current evidence indicates that it is indeed biologically accessible: recent work with *Danio rerio* shows that the compound is readily taken up and rapidly metabolised, demonstrating clear internal exposure (Ren et al. 2026). Because bioavailability is a prerequisite for bioaccumulation, these findings highlight the need to better characterise how accessible 6PPD-Q is across species and exposure pathways.

Beyond demonstrating the sublethal effects of 6PPD‑Q, our results highlight several promising directions for future research. In particular, the strong temperature‑dependent responses we observed indicate that chemical risk assessments should integrate realistic warming scenarios. Moreover, because exposure in natural systems often occurs through pulses and through dietary pathways (e.g., biofilms, organic matter, tire wear particles), future work should evaluate how different exposure routes and episodic contamination events shape long‑term population outcomes. Finally, because growth, reproduction, development and motility emerged as the most sensitive endpoints, linking these responses to internal exposure, through uptake or tissue‑accumulation measurements, will be essential for clarifying the physiological mechanisms behind the observed energy‑allocation trade‑offs and for predicting population resilience under combined chemical and thermal stress.

## Conclusion

In summary, repetitive exposure to 6PPD-Q at environmentally relevant concentrations presents a serious threat to the freshwater snail *Ampullaceana balthica*, affecting key life-history traits of the parental generation, including reproduction, growth, and motility. In the second generation, developmental arrest increased under elevated temperature in the presence of 6PPD-Q, indicating that concurrent impacts across life stages may compromise long-term population viability. Overall, our study underscores the need for further investigation into the sublethal effects of 6PPD-Q on aquatic species and the broader ecological risks associated with this contaminant. The findings contribute to a better understanding of how 6PPD-Q impacts aquatic ecosystems, particularly in the context of climate change and increased contaminant levels. These significant impacts on aquatic life highlight the need for regulatory measures and the development of safer alternatives in the tire industry to address 6PPD-Q pollution.

### Data Availability

All raw data associated with this study are available in the CORA Data Repository at 10.34810/data1906.

## Supplementary Information

Below is the link to the electronic supplementary material.

Supplementary Material 1

## Data Availability

All raw data associated with this study are available in the CORA Data Repository at [https://doi.org/10.34810/data1906](https:/doi.org/10.34810/data1906) .
