# Supplementary material for: Crawling towards complex interactions: the impact of 6PPD-quinone and increased temperatures on the freshwater snail Ampullaceana balthica
Source: Ecotoxicology. 2026 Apr 24;35(5):97. doi: 10.1007/s10646-026-03080-1 (PMC13109286; doi:10.1007/s10646-026-03080-1)
Supplement: Supplementary file 1 — Supplementary Material 1 [file 10646_2026_3080_MOESM1_ESM.docx]

**Crawling towards complex interactions: the impact of 6PPD-quinone and increased temperatures on the freshwater snail *Ampullaceana balthica***

Núria de Castro-Català^1^*, Catalina Lizama, Jordi Serra^1^, Mira Čelić^2^, Isabel Cadena^2^, Mira Petrovic^2,3^, Isabel Muñoz^1^

^1^ Department of Evolutionary Biology, Ecology and Environmental Sciences, Universitat de Barcelona, Av. Diagonal, 643, 08028 Barcelona, Spain.

^2^ Catalan Institute for Water Research (ICRA- CERCA), Carrer Emili Grahit 101, Parc Científic I Tecnològic de la Universitat de Girona, 17003 Girona, Spain.

^3^ Catalan Institution for Research and Advanced Studies (ICREA); Barcelona, Spain.

**Supplementary material**

**Figure S1.** Extracted-ion chromatograms and calibration performance for 6PPD-quinone by UHPLC–Orbitrap–HRMS for multiple calibration levels together with the corresponding linear regression plot, illustrating stable chromatographic behavior (RT ~8.50–8.52 min) and excellent linearity across the calibration range used in the study (example fit shown in the plot; R² ≈ 0.9993). These data support the stated sequence QA/QC based on calibration performance and absence of apparent instrumental drift during analysis.


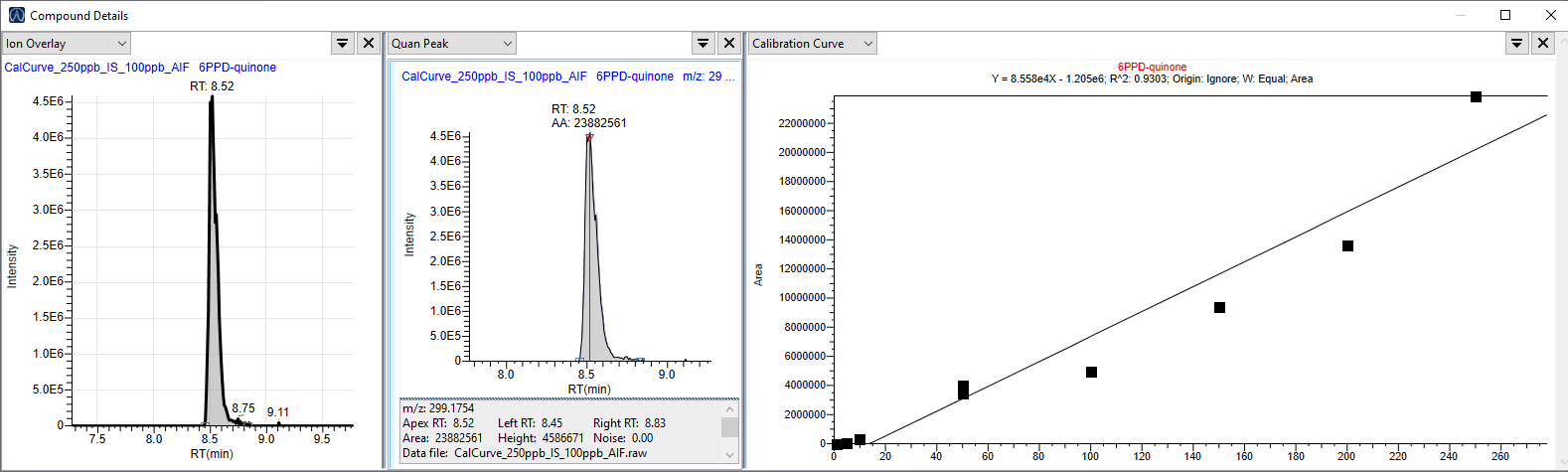

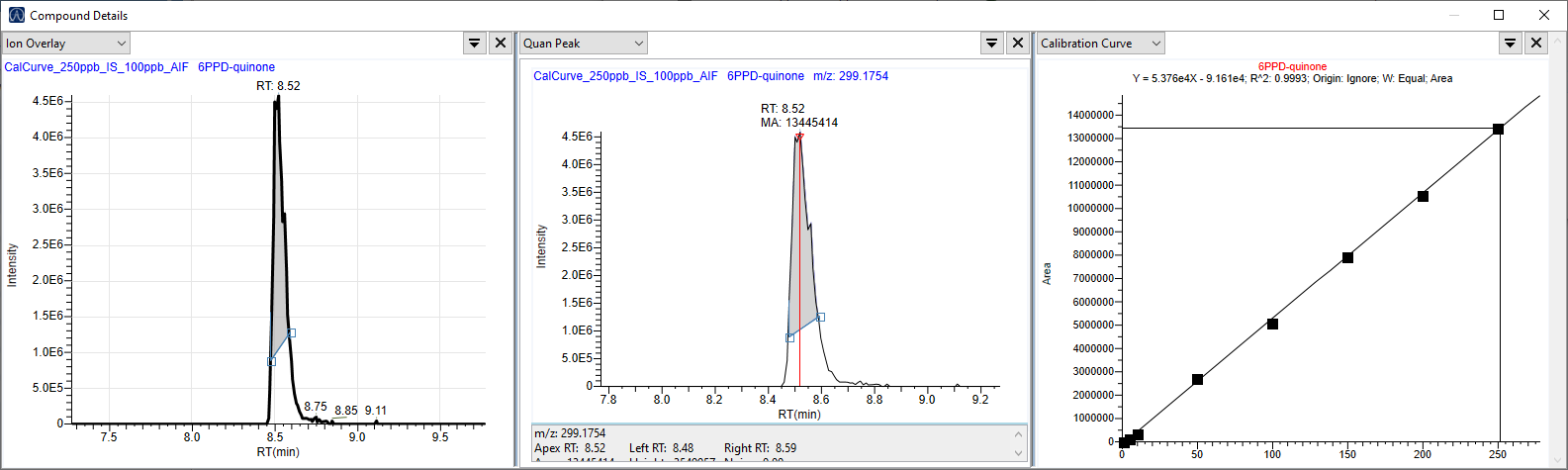

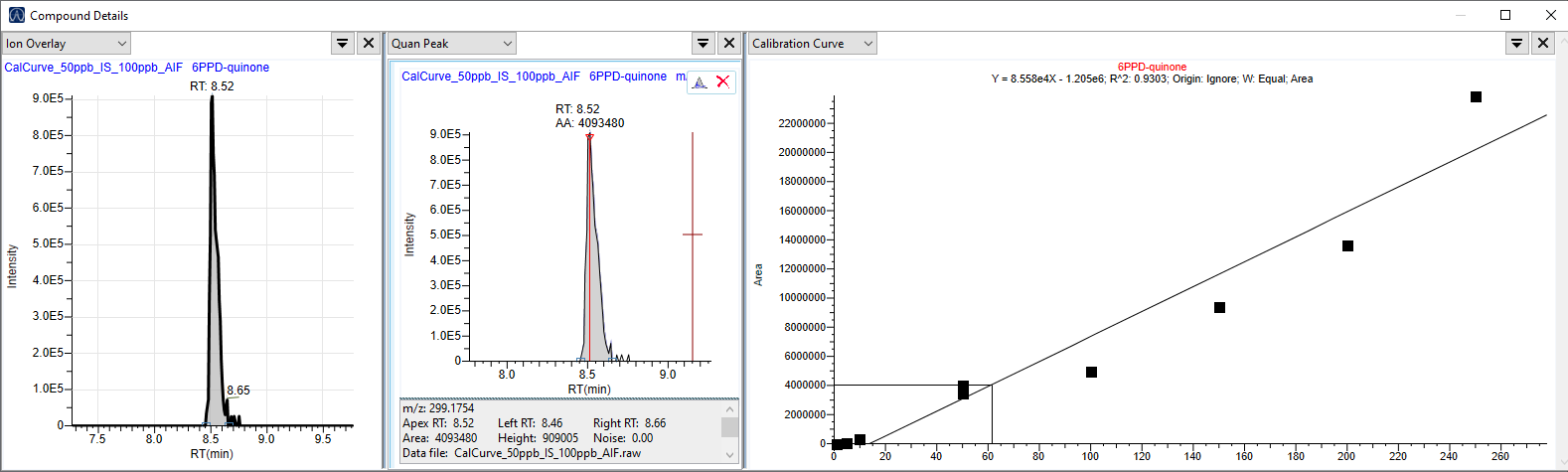

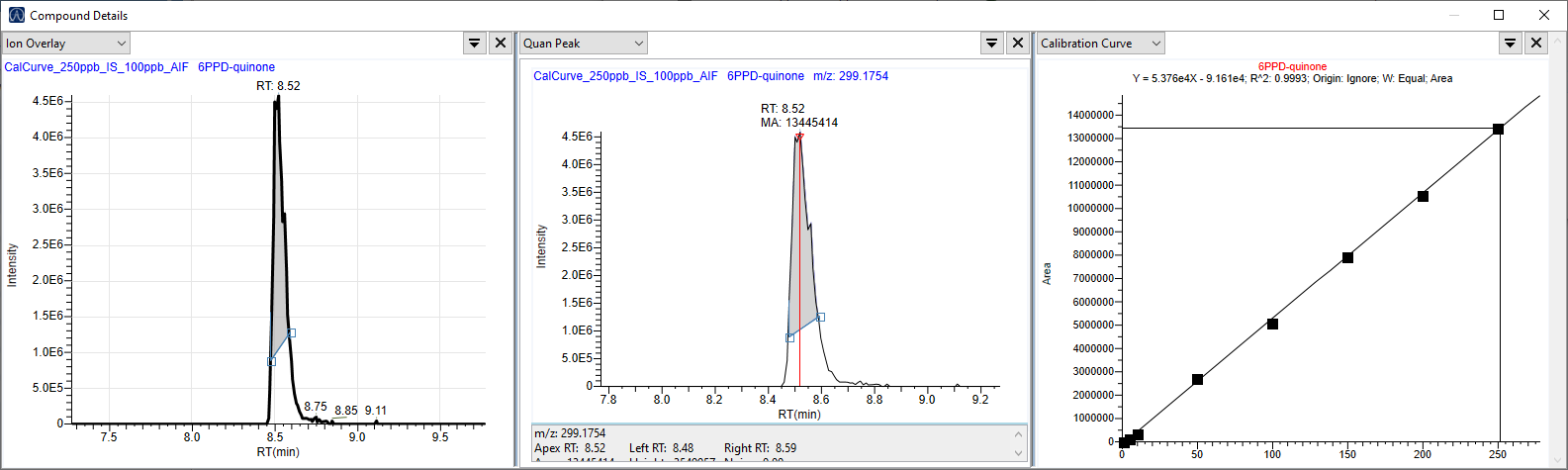

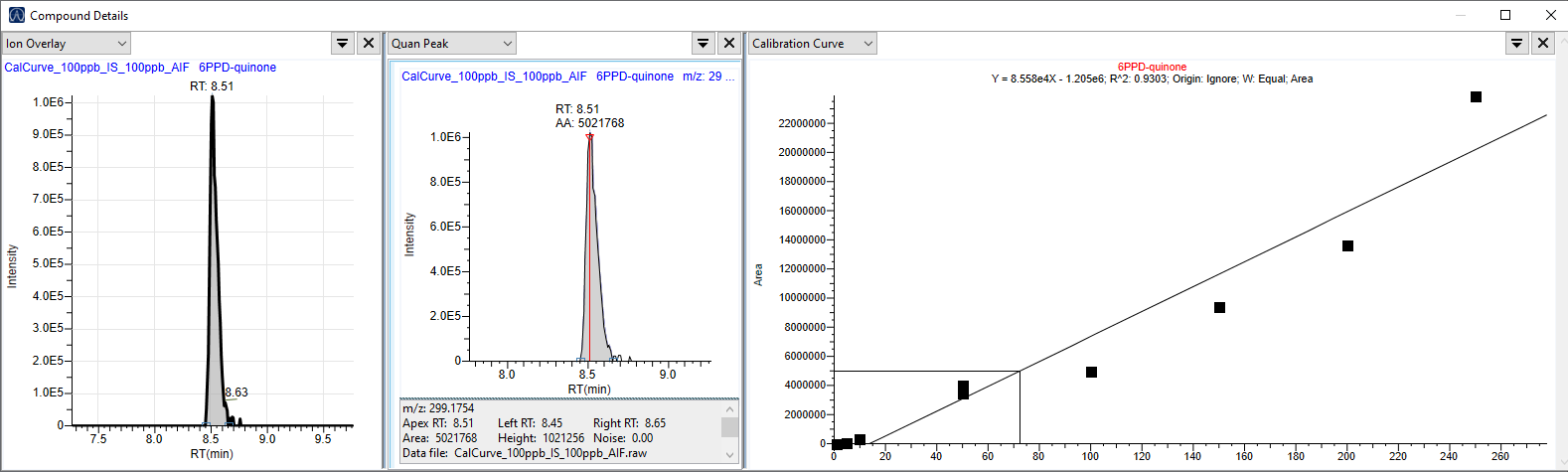

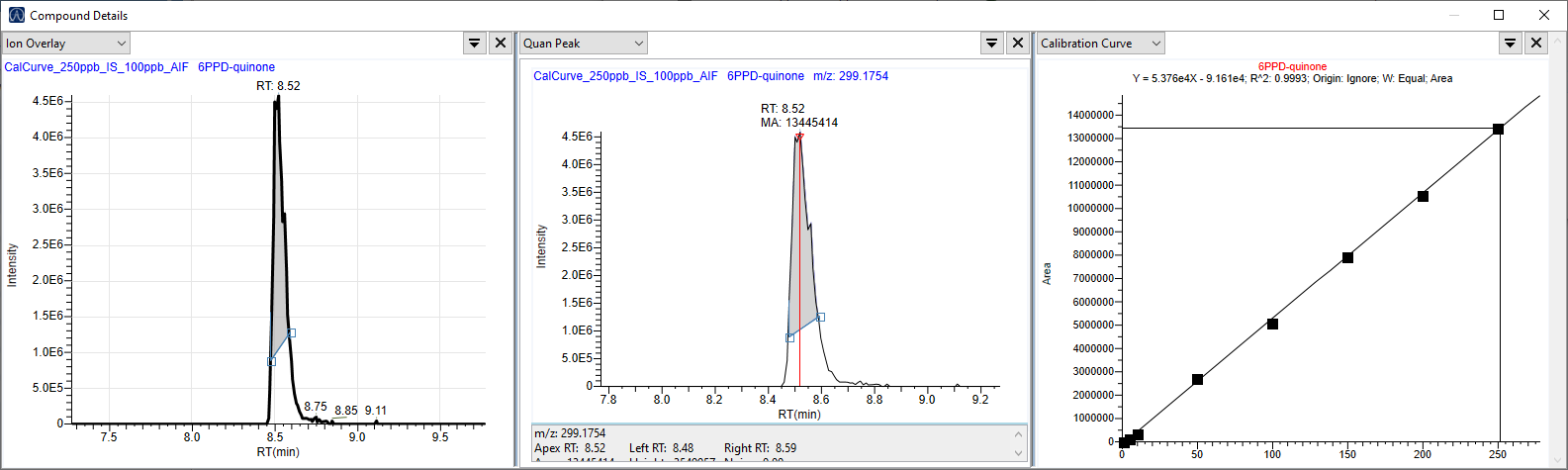

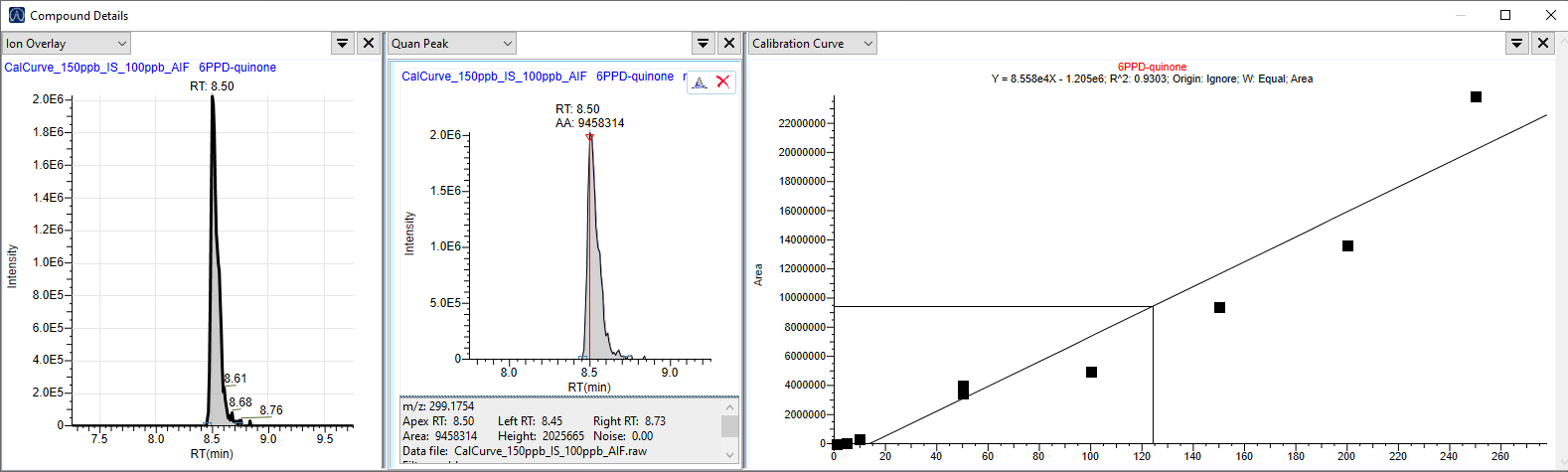

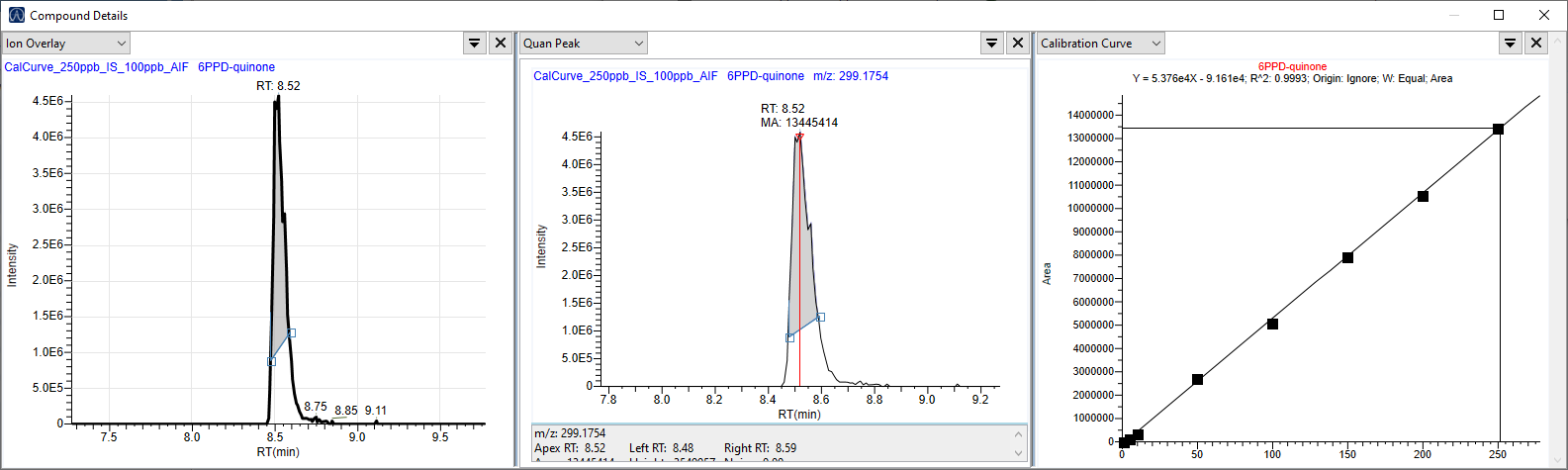

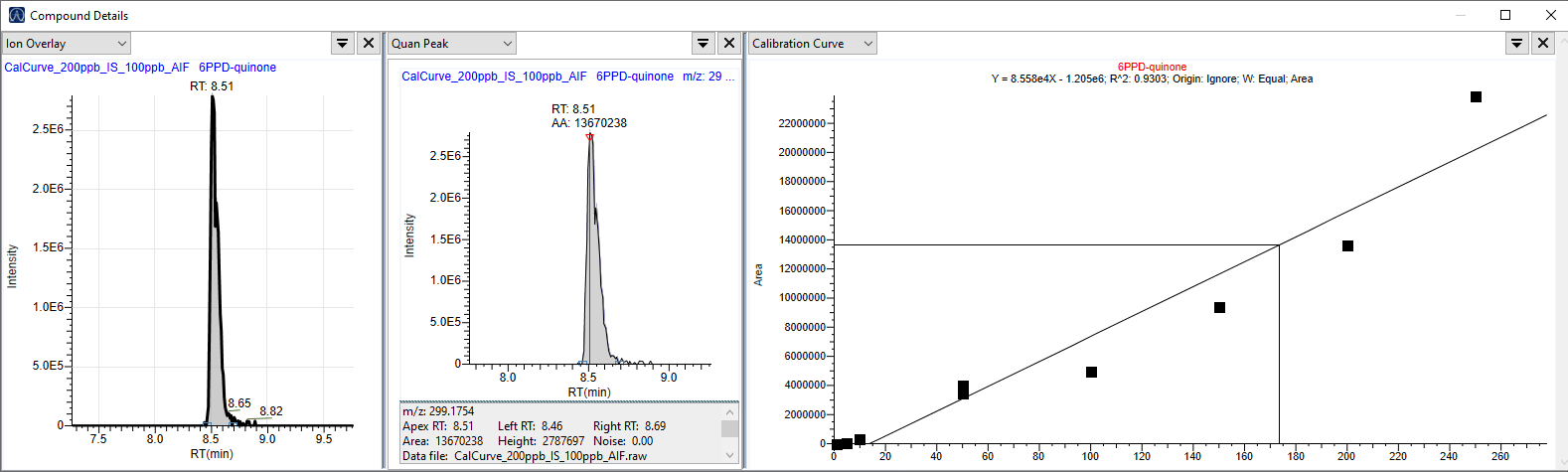

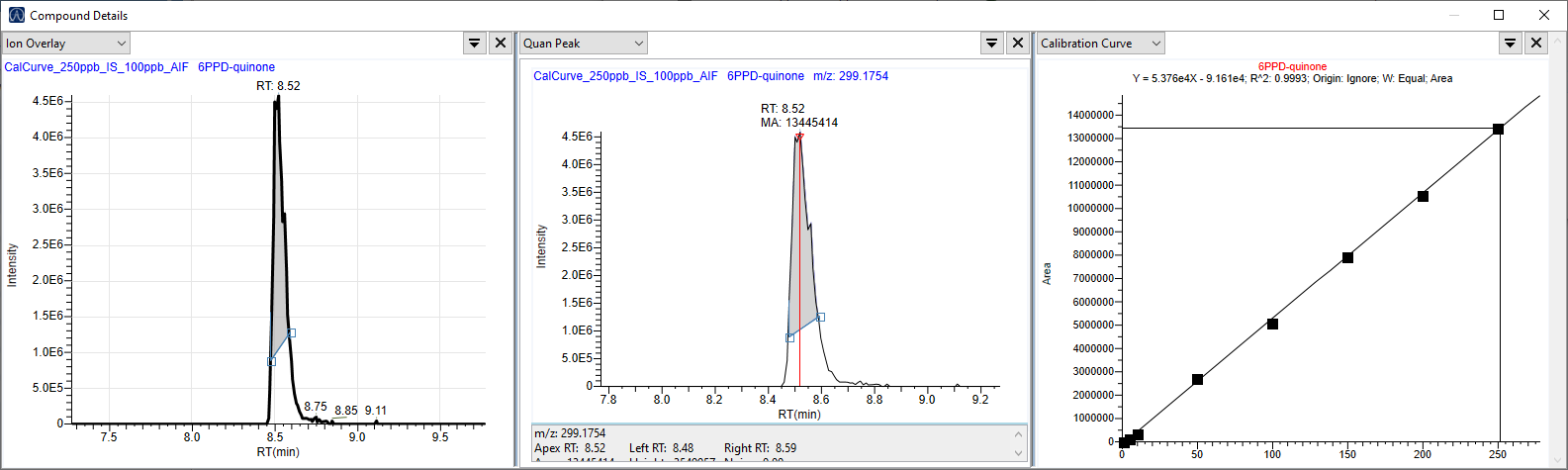


**Figure S2. Instrumental repeatability for 6PPD-quinone assessed by replicate injections (QC/recovery level).** Extracted-ion chromatograms for three replicate injections (n = 3) at 150 µg L⁻¹ (150 ppb) show highly consistent retention time (RT = 8.53 min) and peak area (≈ 1.22 × 10⁷; RSD ≈ 0.43%), demonstrating short-term instrumental reproducibility under the applied UHPLC–Orbitrap–HRMS conditions.


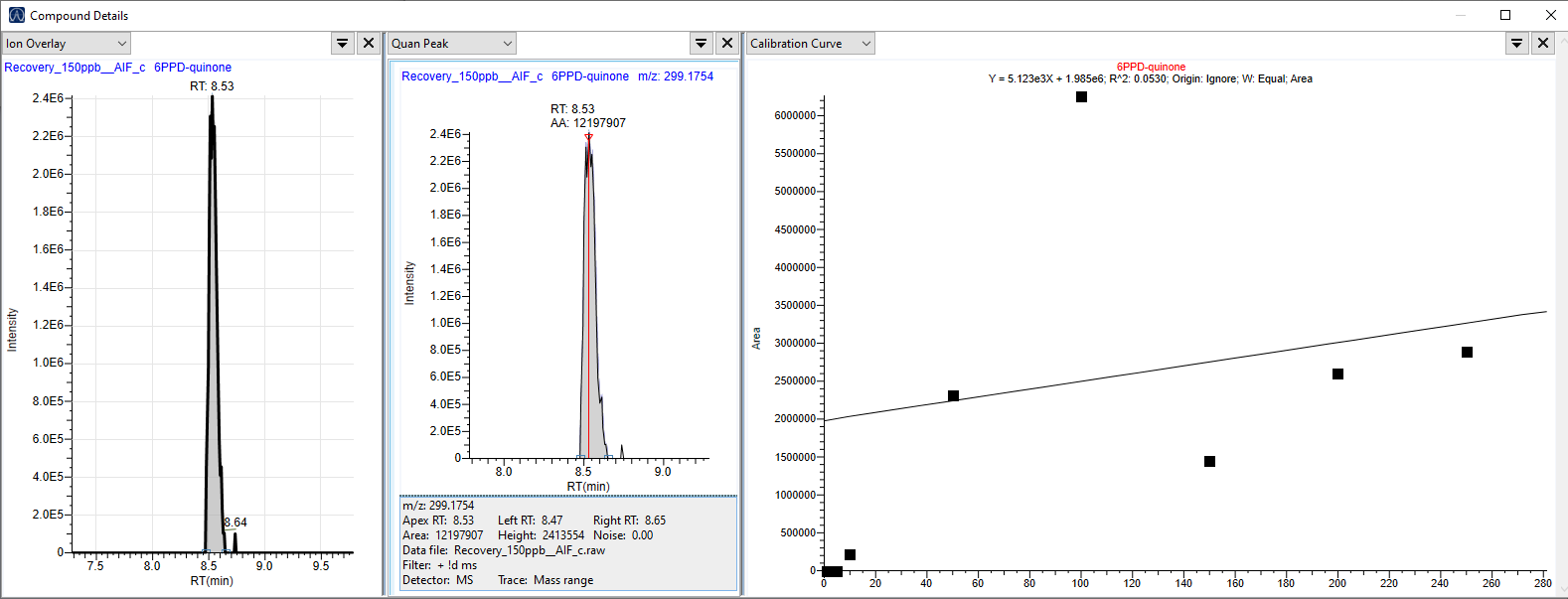

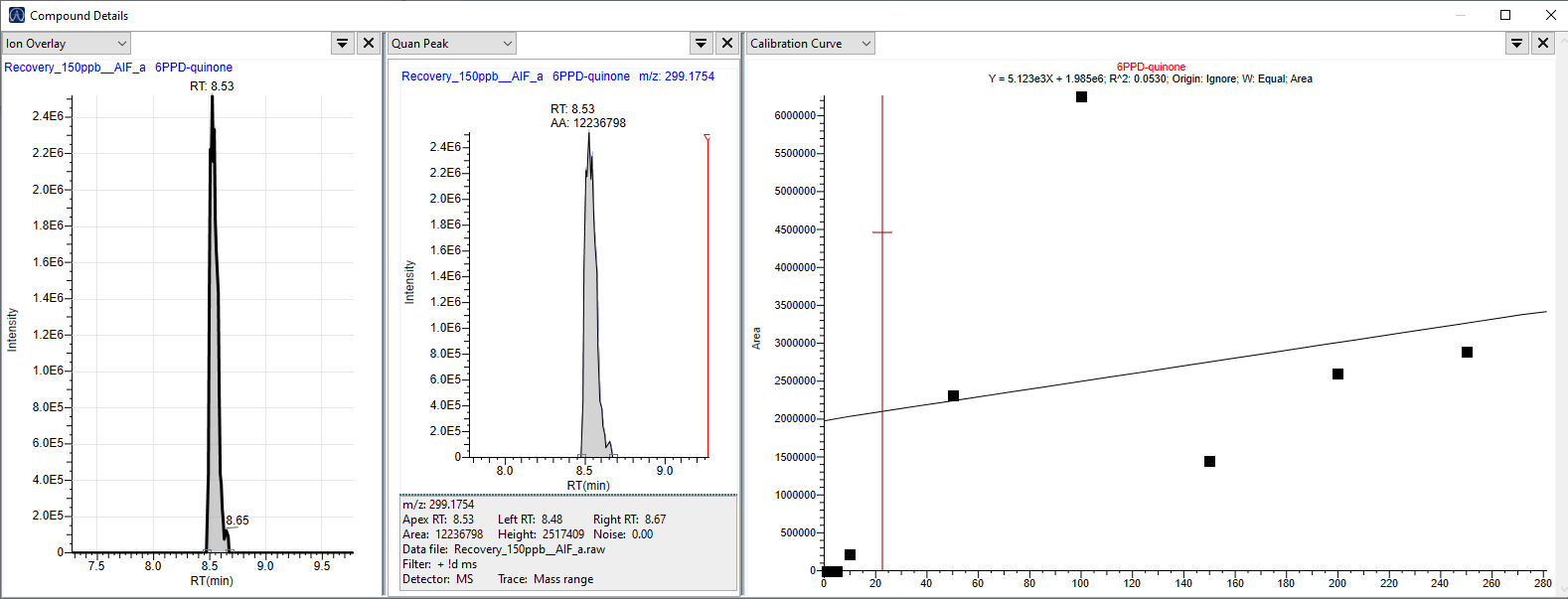

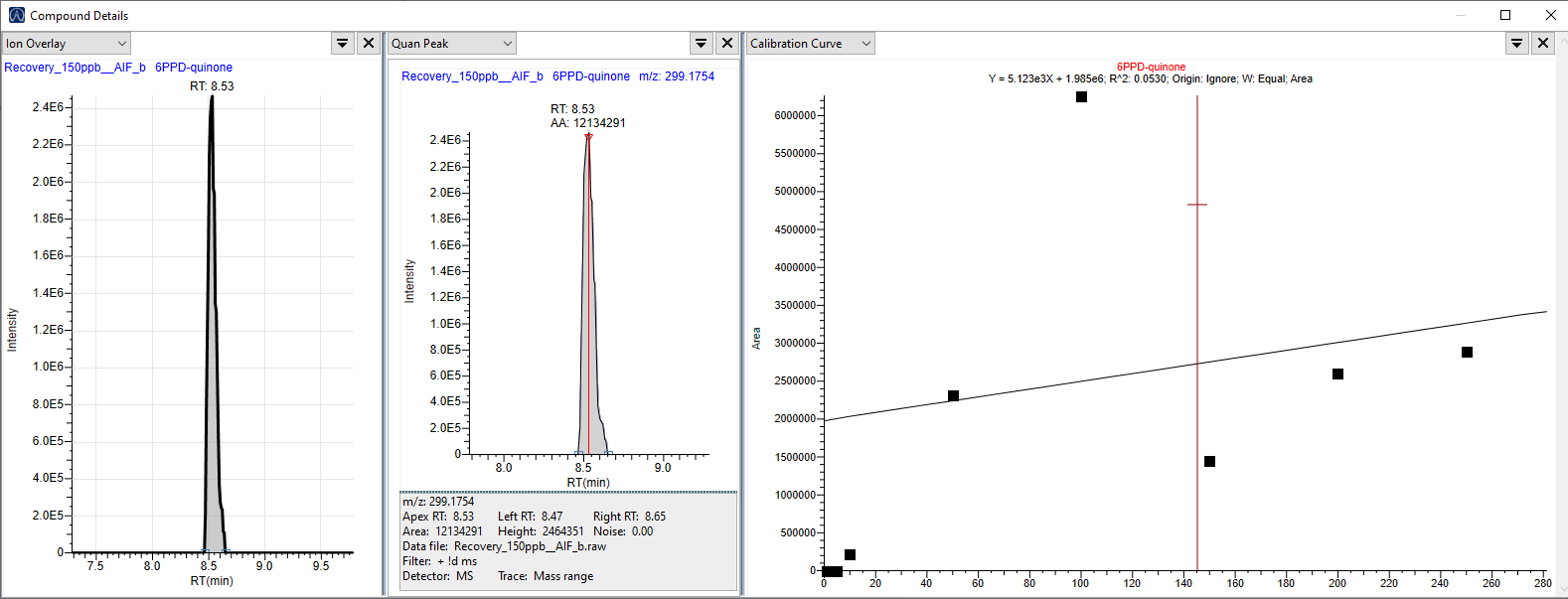


**Figure S3. Chromatograms for 6PPD-Q. A: Calibration standard from Calibration Curve at two concentration levels (low and high); B: Stock solutions of 6PPD-quinone used in the experiment; C: Recovery chromatogram of sample 4, which did not contain any added contaminants.**

**
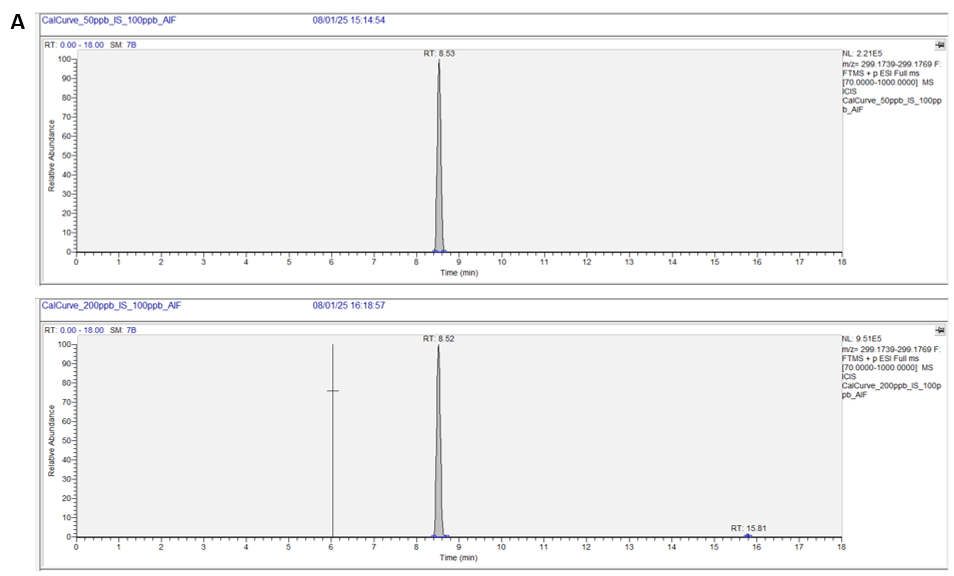
**

**
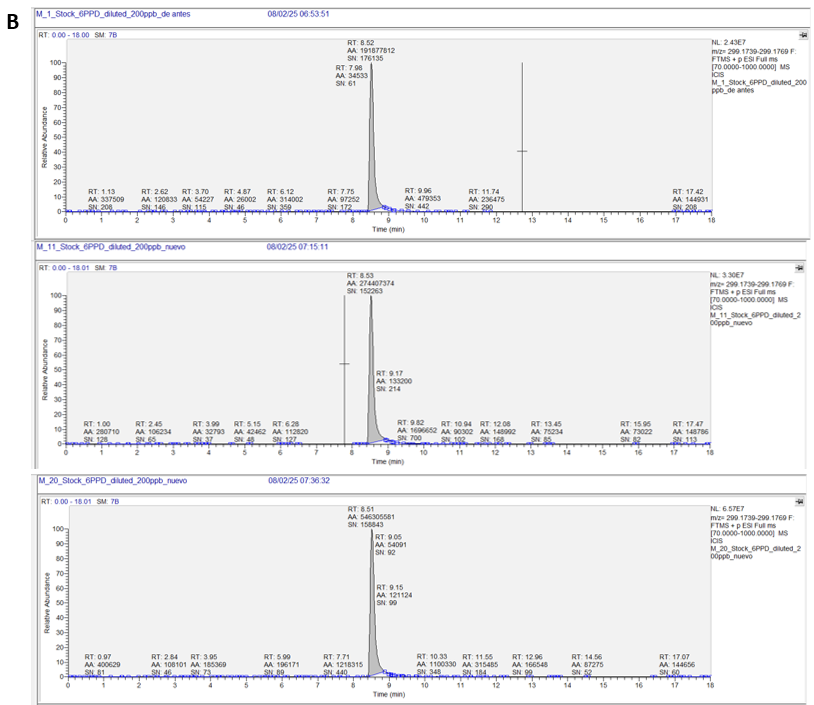
**

**
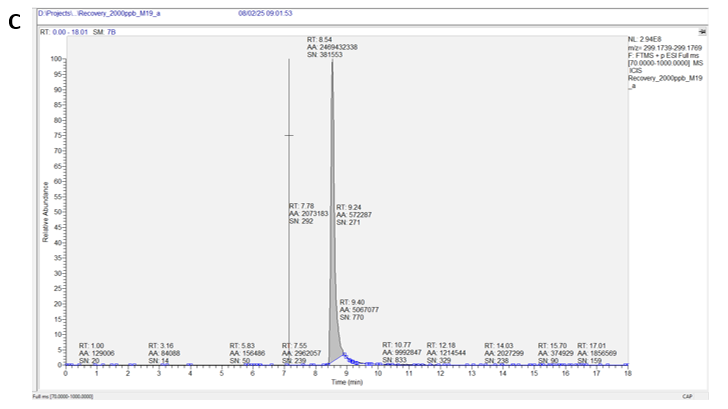
**

**Figure S4. Temperature-dependent degradation of 6PPD-quinone over 48 hours**

**
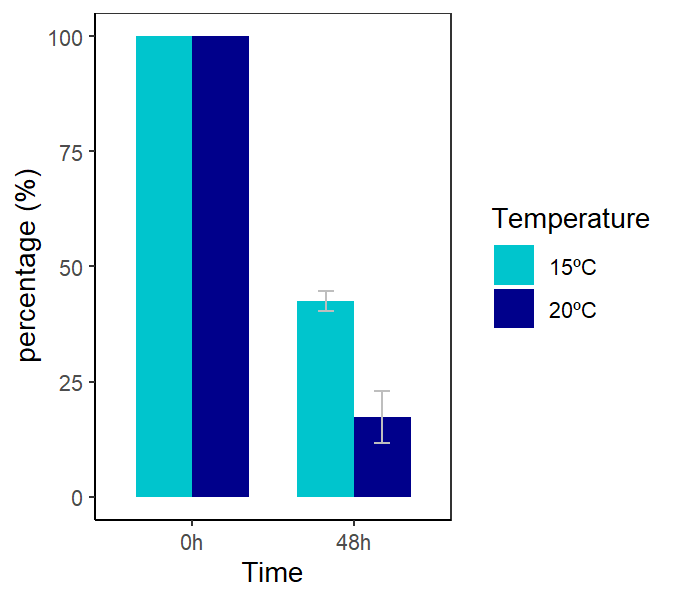
**

# **Table S1. Parameter estimates from AICc-based model selection and model averaging for GLMM, LMM and LM analyses.**

**A)**

| **Endpoint** | **Model (family; link)** | **Random effects** | **Offset** | **Fixed effect** | **Estimate** | **S.E.** | **Test** | **p-value** |
| --- | --- | --- | --- | --- | --- | --- | --- | --- |
| Mortality (deaths/day) | GLMM Poisson; log | Intercept (unit) | log(days) | (Intercept) | -0.7683 | 0.5145 | z=-1.484 | 0.1377 |
|  |  |  |  | Treatment (6PPD-Q) | 0.526 | 0.3303 | z=1.548 | 0.1216 |
|  |  |  |  | Day_c | 0.1157 | 0.058 | z=1.964 | 0.049* |

**R²:** *R²m=0.088; R²c=0.110*

Random intercept variance (S.D.): *0.031 (0.177)*

**B)**

| **Endpoint** | **Model (family; link)** | **Random effects** | **Offset** | **Fixed effect** | **Estimate** | **S.E.** | **Test** | **p-value** |
| --- | --- | --- | --- | --- | --- | --- | --- | --- |
| Clutches (per snail alive) | GLMM Negative Binomial; log | Intercept (unit) | log(snails alive) | (Intercept) | -0.22507 | 1.12728 | z=-0.20 | 0.8418 |
|  |  |  |  | Treatment (6PPD-Q) | -0.7358 | 0.3377 | z=-2.179 | 0.0294* |
|  |  |  |  |  |  |  |  |  |

**R²:** *R²m=0.068; R²c=0.068*

Random intercept variance (S.D.): *0.024 (0.156)*

**C)**

| **Endpoint** | **Model (family; link)** | **Random effects** | **Offset** | **Fixed effect** | **Estimate** | **S.E.** | **Test** | **p-value** |
| --- | --- | --- | --- | --- | --- | --- | --- | --- |
| Eggs per clutch (log) | Model-averaged (Gaussian base) | Intercept (unit) | — | (Intercept) | 1.109 | 0.0491 | z=22.59 | <0.001*** |
|  |  |  |  | Day_c | 0.0229 | 0.0180 | z=1.27 | 0.203 |
|  |  |  |  | Treatment (6PPD-Q) | -0.0349 | 0.0719 | z=0.49 | 0.628 |
|  |  |  |  | Day_c × Treatment | 0.0632 | 0.0310 | z=2.04 | 0.041* |
|  |  |  |  | Temperature (20°C) | 0.0850 | 0.0680 | z=1.25 | 0.212 |

**R²:** *R²m=0.197; R²c=n.a.*

Random intercept variance (S.D.): ~*0 (0)*

**D)**

| **Endpoint** | **Model (family; link)** | **Random effects** | **Offset** | **Fixed effect** | **Estimate** | **S.E.** | **Test** | **p-value** |
| --- | --- | --- | --- | --- | --- | --- | --- | --- |
| Reproductive fitness (eggs/snail/day) | Model averaged (ZI‑GLMM Poisson; log) | Intercept (unit) | — | (Intercept) | 0.777 | 0.204 | z=3.14 | 0.001** |
|  |  |  |  | Temperature (20°C) | 0.838 | 0.293 | z=2.857 | 0.004** |
|  |  |  |  | Treatment (6PPD-Q) | 0.296 | 0.342 | z=0.866 | 0.386 |
|  |  |  |  | Day_c | 0.035 | 0.019 | z=1.824 | 0.07 |
|  |  |  |  | Day_c × Treatment | 0.141 | 0.049 | z=2.887 | 0.003** |
|  |  |  |  | Temperature × Treatment | -0.850 | 0.409 | z=2.076 | 0.037* |
|  |  |  |  | Zero-inflation (Intercept) | -0.693 | 0.369 | z=1.877 | 0.061 |

**R²:** *R²m=0.061; R²c=0.083*

Random intercept variance (S.D.): 0.073 *(0.27)*

**E)**

| **Endpoint** | **Model (family; link)** | **Random effects** | **Offset** | **Fixed effect** | **Estimate** | **S.E.** | **Test** | **p-value** |
| --- | --- | --- | --- | --- | --- | --- | --- | --- |
| Hatching (non-hatched proportion) | GLMM Beta-binomial; logit | Intercept (unit) | — | (Intercept) | -1.766 | 0.318 | z=-5.56 | <0.001*** |
|  |  |  |  | Temperature (20°C) | 2.140 | 0.408 | z=5.24 | <0.001*** |
|  |  |  |  | Temperature × Day_c | 0.482 | 0.163 | z=2.96 | 0.0031** |

**R²:** *R²m=0.782; R²c=0.812*†*.*

Random intercept variance (S.D.): *0.05823 (0.2413)*

†R² values obtain with the binomial equivalent model.

**F)**

| **Endpoint** | **Model (family; link)** | **Random effects** | **Offset** | **Fixed effect** | **Estimate** | **S.E.** | **Test** | **p-value** |
| --- | --- | --- | --- | --- | --- | --- | --- | --- |
| Non-developed embryos | GLMM Binomial; logit | Intercept (unit) | — | (Intercept) | -3.3380 | 0.5794 | z=-5.76 | <0.001*** |
|  |  |  |  | Temperature (20°C) | 1.1645 | 0.6760 | z=1.72 | 0.085 |
|  |  |  |  | Day_c | -0.1355 | 0.2372 | z=-0.57 | 0.568 |
|  |  |  |  | Day_c × Temperature (20°C) | 0.2155 | 0.2590 | z=0.83 | 0.406 |
|  |  |  |  | Treatment (6PPD-Q) | 0.5754 | 0.9263 | z=0.62 | 0.535 |
|  |  |  |  | Day_c × Treatment (6PPD-Q) | -0.8494 | 0.4537 | z=-1.87 | 0.061 |
|  |  |  |  | Temperature (20°C) × Treatment (6PPD-Q) | 0.3357 | 1.6421 | z=0.20 | 0.838 |
|  |  |  |  | Day_c × Temperature (20°C) × Treatment (6PPD-Q) | 1.2862 | 0.5118 | z=2.51 | 0.012* |

**R²:** *R²m=0.442; R²c=0.900*

Random intercept variance (S.D.): *1.126 (1.061)*

**G)**

| **Endpoint** | **Model (family; link)** | **Random effects** | **Offset** | **Fixed effect** | **Estimate** | **S.E.** | **Test** | **p-value** |
| --- | --- | --- | --- | --- | --- | --- | --- | --- |
| Growth (log-growth) | LMM Gaussian; identity | Intercept (unit) | — | (Intercept) | 0.704 | 0.0419 | t=16.82(df=96) | <0.001*** |
|  |  |  |  | Temperature (20°C) | 0.213 | 0.0616 | t=3.46(df=96) | <0.001*** |
|  |  |  |  | Treatment × Temperature | -0.291 | 0.0911 | t=-3.20(df=96) | <0.001*** |

**R²:** *R²m=0.153; R²c=n.a.*

Random intercept variance (S.D.): ~*0 (0)*

**H)**

| **Endpoint** | **Model (family; link)** | **Random effects** | **Offset** | **Fixed effect** | **Estimate** | **S.E.** | **Test** | **p-value** |
| --- | --- | --- | --- | --- | --- | --- | --- | --- |
| CN ratio | LMM Gaussian; identity | Intercept (unit) | — | (Intercept) | 4.416 | 0.0221 | z=195.6 | <0.001*** |
|  |  |  |  | Treatment (6PPD-Q) | 0.0221 | 0.0289 | z=0.752 | 0.195 |
|  |  |  |  | Temperature (20°C) | -0.0419 | 0.0287 | z=1.441 | 0.682 |

**R²:** *R²m=0.029; R²c=0.032*

Random intercept variance (S.D.): *0.0001 (0.007)*

**I)**

| **Endpoint** | **Model (family; link)** | **Random effects** | **Offset** | **Fixed effect** | **Estimate** | **S.E.** | **Test** | **p-value** |
| --- | --- | --- | --- | --- | --- | --- | --- | --- |
| Motility (velocity) | LM Gaussian; identity | — | — | (Intercept) | 1.109 | 0.142 | t=7.83 (df=8) | <0.001*** |
|  |  |  |  | Treatment (6PPD-Q) | -0.515 | 0.200 | t=-2.57 (df=8) | 0.033* |
|  |  |  |  | Temperature (20°C) | -0.473 | 0.200 | t=-2.36 (df=8) | 0.046* |
|  |  |  |  | Treatment × Temperature | 0.453 | 0.284 | t=1.60 (df=8) | 0.149 |

**R²:** *R²=0.548; adj.R²=0.379*

p-values: *** <0.001; ** <0.01; * <0.05. R²m: R²marginal; and R²c: R²conditional.

# **Text S1. Statistical modelling workflow and diagnostics (R Markdown)**

# 1. Mortality

#1.1. Load the data

# Load data
mortality <- read.csv("mortality.csv", sep = ";", header = TRUE)
summary(mortality)

## replicate Treatment Temperature Day
## Min. :1.000 Length:47 Min. :15.00 Min. : 2.000
## 1st Qu.:1.000 Class :character 1st Qu.:15.00 1st Qu.: 3.000
## Median :2.000 Mode :character Median :15.00 Median : 7.000
## Mean :1.979 Mean :17.45 Mean : 5.787
## 3rd Qu.:3.000 3rd Qu.:20.00 3rd Qu.: 8.500
## Max. :3.000 Max. :20.00 Max. :10.000
##
## days deadf deaddac deaddac2
## Min. :2.000 Min. :1.00 Min. :0.000 Min. :0.000
## 1st Qu.:2.000 1st Qu.:2.00 1st Qu.:0.000 1st Qu.:0.000
## Median :3.000 Median :3.00 Median :2.000 Median :2.000
## Mean :2.511 Mean :3.69 Mean :2.106 Mean :2.064
## 3rd Qu.:3.000 3rd Qu.:4.00 3rd Qu.:3.000 3rd Qu.:3.000
## Max. :3.000 Max. :8.00 Max. :8.000 Max. :8.000
## NA's :18
## deadd dailydead logdailydead alive
## Min. :0.0000 Min. :0.0000 Min. :0.0000 Min. : 4.000
## 1st Qu.:0.0000 1st Qu.:0.0000 1st Qu.:0.0000 1st Qu.: 9.000
## Median :1.0000 Median :0.3333 Median :0.1249 Median :10.000
## Mean :0.9362 Mean :0.3652 Mean :0.1187 Mean : 9.872
## 3rd Qu.:1.0000 3rd Qu.:0.5000 3rd Qu.:0.1761 3rd Qu.:12.000
## Max. :6.0000 Max. :2.0000 Max. :0.4771 Max. :12.000
##

# Factors & basic cleaning
mortality$Treatment <- factor(mortality$Treatment, levels = c("CTL","6PPD-Q"))
mortality$Temperature <- factor(mortality$Temperature) # 15 / 20 as factor
mortality$Day_c <- scale(mortality$Day, center=TRUE, scale=FALSE)
mortality$unit <- interaction(mortality$Treatment, mortality$Temperature, mortality$replicate) # random effect for "unit"

summary(mortality)

## replicate Treatment Temperature Day days
## Min. :1.000 CTL :23 15:24 Min. : 2.000 Min. :2.000
## 1st Qu.:1.000 6PPD-Q:24 20:23 1st Qu.: 3.000 1st Qu.:2.000
## Median :2.000 Median : 7.000 Median :3.000
## Mean :1.979 Mean : 5.787 Mean :2.511
## 3rd Qu.:3.000 3rd Qu.: 8.500 3rd Qu.:3.000
## Max. :3.000 Max. :10.000 Max. :3.000
##
## deadf deaddac deaddac2 deadd
## Min. :1.00 Min. :0.000 Min. :0.000 Min. :0.0000
## 1st Qu.:2.00 1st Qu.:0.000 1st Qu.:0.000 1st Qu.:0.0000
## Median :3.00 Median :2.000 Median :2.000 Median :1.0000
## Mean :3.69 Mean :2.106 Mean :2.064 Mean :0.9362
## 3rd Qu.:4.00 3rd Qu.:3.000 3rd Qu.:3.000 3rd Qu.:1.0000
## Max. :8.00 Max. :8.000 Max. :8.000 Max. :6.0000
## NA's :18
## dailydead logdailydead alive Day_c.V1
## Min. :0.0000 Min. :0.0000 Min. : 4.000 Min. :-3.78723404255
## 1st Qu.:0.0000 1st Qu.:0.0000 1st Qu.: 9.000 1st Qu.:-2.78723404255
## Median :0.3333 Median :0.1249 Median :10.000 Median : 1.21276595745
## Mean :0.3652 Mean :0.1187 Mean : 9.872 Mean : 0.00000000000
## 3rd Qu.:0.5000 3rd Qu.:0.1761 3rd Qu.:12.000 3rd Qu.: 2.71276595745
## Max. :2.0000 Max. :0.4771 Max. :12.000 Max. : 4.21276595745
##
## unit
## CTL.15.1 : 4
## 6PPD-Q.15.1: 4
## CTL.20.1 : 4
## 6PPD-Q.20.1: 4
## CTL.15.2 : 4
## 6PPD-Q.15.2: 4
## (Other) :23

hist(mortality$dailydead,
 breaks = 20,
 col = "grey",
 main = "Daily deaths distribution",
 xlab = "dailydead")


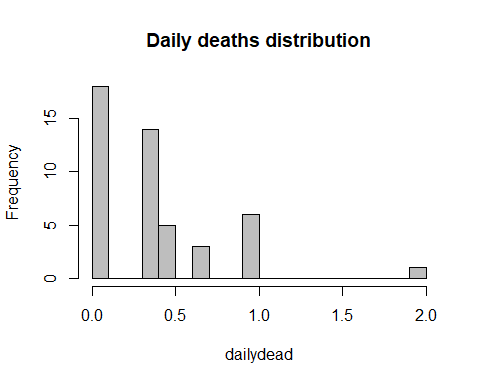


#1.2. LMM with random effects

mod_mortality_RE <- lmer(
 logdailydead ~ Treatment * Temperature*Day_c + (1 | unit),
 data = mortality,
 REML = FALSE
)
summary(mod_mortality_RE)

## Linear mixed model fit by maximum likelihood ['lmerMod']
## Formula: logdailydead ~ Treatment * Temperature * Day_c + (1 | unit)
## Data: mortality
##
## AIC BIC logLik -2*log(L) df.resid
## -60.0 -41.5 40.0 -80.0 37
##
## Scaled residuals:
## Min 1Q Median 3Q Max
## -1.5286 -0.6764 -0.2940 0.6004 2.1782
##
## Random effects:
## Groups Name Variance Std.Dev.
## unit (Intercept) 0.00000 0.0000
## Residual 0.01067 0.1033
## Number of obs: 47, groups: unit, 12
##
## Fixed effects:
## Estimate Std. Error t value
## (Intercept) 0.089677 0.029824 3.007
## Treatment6PPD-Q 0.065508 0.042178 1.553
## Temperature20 0.001339 0.043142 0.031
## Day_c 0.005228 0.009839 0.531
## Treatment6PPD-Q:Temperature20 -0.020902 0.060334 -0.346
## Treatment6PPD-Q:Day_c 0.017777 0.013914 1.278
## Temperature20:Day_c 0.010783 0.014022 0.769
## Treatment6PPD-Q:Temperature20:Day_c -0.039675 0.019754 -2.008
##
## Correlation of Fixed Effects:
## (Intr) Tr6PPD-Q Tmpr20 Day_c Tr6PPD-Q:T20 T6PPD-Q:D T20:D_
## Trtmn6PPD-Q -0.707
## Temperatr20 -0.691 0.489
## Day_c 0.012 -0.009 -0.008
## Tr6PPD-Q:T20 0.494 -0.699 -0.715 0.006
## Tr6PPD-Q:D_ -0.009 0.012 0.006 -0.707 -0.009
## Tmprtr20:D_ -0.009 0.006 -0.014 -0.702 0.010 0.496
## T6PPD-Q:T20: 0.006 -0.009 0.010 0.498 -0.001 -0.704 -0.710
## optimizer (nloptwrap) convergence code: 0 (OK)
## boundary (singular) fit: see help('isSingular')

tapply(mortality$logdailydead, mortality$unit, var)

## CTL.15.1 6PPD-Q.15.1 CTL.20.1 6PPD-Q.20.1 CTL.15.2 6PPD-Q.15.2
## 0.007987685 0.039160206 0.005621722 0.007987685 0.020288802 0.005621722
## CTL.20.2 6PPD-Q.20.2 CTL.15.3 6PPD-Q.15.3 CTL.20.3 6PPD-Q.20.3
## 0.005203229 0.020288802 0.011587055 0.005621722 0.012369684 0.022654765

boxplot(logdailydead ~ unit, data=mortality)


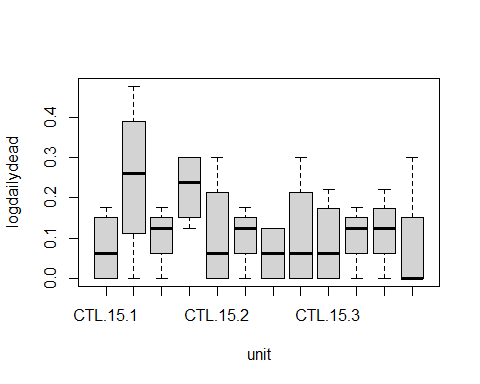


library(lme4); library(performance)

mod_mortRE_REML <- lmer(logdailydead ~ Treatment * Temperature*Day_c + (1 | unit),
 data = mortality, REML = TRUE)
VarCorr(mod_mortRE_REML) # mira la variància de (Intercept)|unit

## Groups Name Std.Dev.
## unit (Intercept) 0.021357
## Residual 0.111782

performance::icc(mod_mortRE_REML) # Intraclass Correlation Coefficient

## # Intraclass Correlation Coefficient
##
## Adjusted ICC: 0.035
## Unadjusted ICC: 0.029

isSingular(mod_mortRE_REML)

## [1] FALSE

#1.3. LM without random effects

mod_mortality_noRE <- lm(
 logdailydead ~ Treatment * Temperature*Day_c,
 data = mortality
)
summary(mod_mortality_noRE)

##
## Call:
## lm(formula = logdailydead ~ Treatment * Temperature * Day_c,
## data = mortality)
##
## Residuals:
## Min 1Q Median 3Q Max
## -0.15792 -0.06988 -0.03038 0.06202 0.22502
##
## Coefficients:
## Estimate Std. Error t value Pr(>|t|)
## (Intercept) 0.089677 0.032741 2.739 0.00924 **
## Treatment6PPD-Q 0.065508 0.046303 1.415 0.16507
## Temperature20 0.001339 0.047360 0.028 0.97759
## Day_c 0.005228 0.010801 0.484 0.63105
## Treatment6PPD-Q:Temperature20 -0.020902 0.066234 -0.316 0.75401
## Treatment6PPD-Q:Day_c 0.017777 0.015275 1.164 0.25158
## Temperature20:Day_c 0.010783 0.015394 0.701 0.48777
## Treatment6PPD-Q:Temperature20:Day_c -0.039675 0.021686 -1.830 0.07497 .
## ---
## Signif. codes: 0 '***' 0.001 '**' 0.01 '*' 0.05 '.' 0.1 ' ' 1
##
## Residual standard error: 0.1134 on 39 degrees of freedom
## Multiple R-squared: 0.2044, Adjusted R-squared: 0.06161
## F-statistic: 1.431 on 7 and 39 DF, p-value: 0.2207

#1.4. compare models

#Likelihood-ratio test (LMM vs LM)
anova(mod_mortality_RE, mod_mortality_noRE)

## Data: mortality
## Models:
## mod_mortality_noRE: logdailydead ~ Treatment * Temperature * Day_c
## mod_mortality_RE: logdailydead ~ Treatment * Temperature * Day_c + (1 | unit)
## npar AIC BIC logLik -2*log(L) Chisq Df Pr(>Chisq)
## mod_mortality_noRE 9 -62.004 -45.353 40.002 -80.004
## mod_mortality_RE 10 -60.004 -41.503 40.002 -80.004 0 1 1

# AICc comparison
AICc(mod_mortality_RE); AICc(mod_mortality_noRE)

## [1] -53.8931

## [1] -57.13935

# Select best by AICc
#mods_growth <- list(RE = mod_growth_RE, noRE = mod_growth_noRE)
#best_growth <- mods_growth[[ which.min(sapply(mods_growth, AICc)) ]]
#best_growth
#summary(best_growth)

#1.6. LMM with random effects model selection

# Dredge over the fixed-effects structure of the LMM
options(na.action = "na.fail")
dredge_mortality <- dredge(mod_mortality_RE, rank = "AICc")
dredge_mortality

## Global model call: lmer(formula = logdailydead ~ Treatment * Temperature * Day_c +
## (1 | unit), data = mortality, REML = FALSE)
## ---
## Model selection table
## (Int) Day_c Tmp Trt Day_c:Tmp Day_c:Trt Tmp:Trt Day_c:Tmp:Trt df
## 6 0.09078 0.009545 + 5
## 2 0.11870 0.009433 4
## 5 0.09115 + 4
## 1 0.11870 3
## 8 0.09484 0.009562 + + 6
## 22 0.09074 0.010540 + + 6
## 4 0.12230 0.009448 + 5
## 7 0.09485 + + 5
## 3 0.12190 + 4
## 16 0.09519 0.014120 + + + 7
## 12 0.12240 0.014120 + + 6
## 40 0.08984 0.009540 + + + 7
## 24 0.09484 0.010580 + + + 7
## 39 0.08948 + + + 6
## 48 0.09001 0.014120 + + + + 8
## 32 0.09518 0.015090 + + + + 8
## 56 0.08987 0.010540 + + + + 8
## 128 0.08968 0.005228 + + + + + + 10
## 64 0.09004 0.015070 + + + + + 9
## logLik AICc delta weight
## 6 37.564 -63.7 0.00 0.208
## 2 36.128 -63.3 0.36 0.174
## 5 35.938 -62.9 0.74 0.144
## 1 34.628 -62.7 0.97 0.128
## 8 37.600 -61.1 2.57 0.058
## 22 37.583 -61.1 2.60 0.057
## 4 36.153 -60.8 2.82 0.051
## 7 35.965 -60.5 3.20 0.042
## 3 34.647 -60.3 3.32 0.039
## 16 37.993 -59.1 4.55 0.021
## 12 36.543 -59.0 4.68 0.020
## 40 37.652 -58.4 5.23 0.015
## 24 37.619 -58.4 5.30 0.015
## 39 36.021 -57.9 5.72 0.012
## 48 38.050 -56.3 7.35 0.005
## 32 38.011 -56.2 7.43 0.005
## 56 37.670 -55.6 8.11 0.004
## 128 40.002 -53.9 9.77 0.002
## 64 38.067 -53.3 10.40 0.001
## Models ranked by AICc(x)
## Random terms (all models):
## 1 | unit

# Select models with ΔAICc < 2 (or up to 95% cumulative weight)
avgset_mortality <- subset(dredge_mortality, delta <= 2)
#if (nrow(avgset_mortality) == 0) {
# avgset_mortality <- dredge_mortality[cumsum(dredge_mortality$weight) <= 0.95, ]}

# Model averaging
avg_mortality <- model.avg(avgset_mortality)
summary(avg_mortality)

##
## Call:
## model.avg(object = avgset_mortality)
##
## Component model call:
## lmer(formula = logdailydead ~ <4 unique rhs>, data = mortality, REML =
## FALSE)
##
## Component models:
## df logLik AICc delta weight
## 12 5 37.56 -63.67 0.00 0.32
## 1 4 36.13 -63.30 0.36 0.27
## 2 4 35.94 -62.92 0.74 0.22
## (Null) 3 34.63 -62.70 0.97 0.20
##
## Term codes:
## Day_c Treatment
## 1 2
##
## Model-averaged coefficients:
## (full average)
## Estimate Std. Error Adjusted SE z value Pr(>|z|)
## (Intercept) 0.103755 0.024590 0.025081 4.137 3.52e-05 ***
## Day_c 0.005545 0.006173 0.006251 0.887 0.375
## Treatment6PPD-Q 0.029248 0.035957 0.036417 0.803 0.422
##
## (conditional average)
## Estimate Std. Error Adjusted SE z value Pr(>|z|)
## (Intercept) 0.103755 0.024590 0.025081 4.137 3.52e-05 ***
## Day_c 0.009494 0.005269 0.005424 1.751 0.080 .
## Treatment6PPD-Q 0.054365 0.032214 0.033159 1.640 0.101
## ---
## Signif. codes: 0 '***' 0.001 '**' 0.01 '*' 0.05 '.' 0.1 ' ' 1

# Sum of weights by term
sw_motility <- sw(avgset_mortality)
sw_motility

## Day_c Treatment
## Sum of weights: 0.58 0.54
## N containing models: 2 2

## 1.5. Diagnosis final model with random effectes (lmer)

#final model:
mod_avgmortality_RE <- lmer(
 logdailydead ~ Treatment + Day_c + (1 | unit),
 data = mortality,
 REML = FALSE
)
summary(mod_avgmortality_RE)

## Linear mixed model fit by maximum likelihood ['lmerMod']
## Formula: logdailydead ~ Treatment + Day_c + (1 | unit)
## Data: mortality
##
## AIC BIC logLik -2*log(L) df.resid
## -65.1 -55.9 37.6 -75.1 42
##
## Scaled residuals:
## Min 1Q Median 3Q Max
## -1.7062 -0.6177 -0.2948 0.6049 2.6788
##
## Random effects:
## Groups Name Variance Std.Dev.
## unit (Intercept) 0.00000 0.0000
## Residual 0.01184 0.1088
## Number of obs: 47, groups: unit, 12
##
## Fixed effects:
## Estimate Std. Error t value
## (Intercept) 0.090778 0.022689 4.001
## Treatment6PPD-Q 0.054662 0.031752 1.722
## Day_c 0.009545 0.005200 1.835
##
## Correlation of Fixed Effects:
## (Intr) T6PPD-
## Trtmn6PPD-Q -0.715
## Day_c -0.009 0.012
## optimizer (nloptwrap) convergence code: 0 (OK)
## boundary (singular) fit: see help('isSingular')

check_model(mod_avgmortality_RE)
MuMIn::AICc(mod_avgmortality_RE)

## [1] -63.66541

performance::r2(mod_avgmortality_RE)

## Random effect variances not available. Returned R2 does not account for random effects.

## # R2 for Mixed Models
##
## Conditional R2: NA
## Marginal R2: 0.120

res <- simulateResiduals(mod_avgmortality_RE)
plot(res)
testDispersion(res)

##
## DHARMa nonparametric dispersion test via sd of residuals fitted vs.
## simulated
##
## data: simulationOutput
## dispersion = 1.0194, p-value = 0.912
## alternative hypothesis: two.sided

testZeroInflation(res) #there is zero inflation

##
## DHARMa zero-inflation test via comparison to expected zeros with
## simulation under H0 = fitted model
##
## data: simulationOutput
## ratioObsSim = Inf, p-value < 2.2e-16
## alternative hypothesis: two.sided

testUniformity(res)

##
## Asymptotic one-sample Kolmogorov-Smirnov test
##
## data: simulationOutput$scaledResiduals
## D = 0.12264, p-value = 0.4795
## alternative hypothesis: two-sided

# 1.6. GLMER Poisson with offset (days)

mod_mort_poisRE <- glmer(deadd ~ Treatment * Temperature * Day_c +
 offset(log(days)) +
 (1 | unit),
 data = mortality,
 family = poisson(link = "log")
)

summary(mod_mort_poisRE)

## Generalized linear mixed model fit by maximum likelihood (Laplace
## Approximation) [glmerMod]
## Family: poisson ( log )
## Formula: deadd ~ Treatment * Temperature * Day_c + offset(log(days)) +
## (1 | unit)
## Data: mortality
##
## AIC BIC logLik -2*log(L) df.resid
## 126.8 143.4 -54.4 108.8 38
##
## Scaled residuals:
## Min 1Q Median 3Q Max
## -1.0470 -0.6725 -0.2839 0.5262 2.1703
##
## Random effects:
## Groups Name Variance Std.Dev.
## unit (Intercept) 0.01807 0.1344
## Number of obs: 47, groups: unit, 12
##
## Fixed effects:
## Estimate Std. Error z value Pr(>|z|)
## (Intercept) -1.35497 0.37808 -3.584 0.000339 ***
## Treatment6PPD-Q 0.49372 0.49357 1.000 0.317163
## Temperature20 -0.11323 0.58251 -0.194 0.845880
## Day_c 0.03639 0.11898 0.306 0.759694
## Treatment6PPD-Q:Temperature20 0.06356 0.72885 0.087 0.930504
## Treatment6PPD-Q:Day_c 0.13959 0.15159 0.921 0.357159
## Temperature20:Day_c 0.11753 0.17782 0.661 0.508640
## Treatment6PPD-Q:Temperature20:Day_c -0.34514 0.22289 -1.548 0.121513
## ---
## Signif. codes: 0 '***' 0.001 '**' 0.01 '*' 0.05 '.' 0.1 ' ' 1
##
## Correlation of Fixed Effects:
## (Intr) Tr6PPD-Q Tmpr20 Day_c Tr6PPD-Q:T20 T6PPD-Q:D T20:D_
## Trtmn6PPD-Q -0.753
## Temperatr20 -0.641 0.490
## Day_c -0.264 0.202 0.171
## Tr6PPD-Q:T20 0.511 -0.677 -0.799 -0.137
## Tr6PPD-Q:D_ 0.207 -0.390 -0.134 -0.785 0.264
## Tmprtr20:D_ 0.177 -0.135 -0.446 -0.669 0.356 0.525
## T6PPD-Q:T20: -0.141 0.265 0.355 0.534 -0.393 -0.680 -0.798

**Model selection**

# Dredge over the fixed-effects structure of the LMM
options(na.action = "na.fail")
dredge_mortality_pois <- dredge(mod_mort_poisRE, rank = "AICc")
dredge_mortality_pois

## Global model call: glmer(formula = deadd ~ Treatment * Temperature * Day_c + offset(log(days)) +
## (1 | unit), data = mortality, family = poisson(link = "log"))
## ---
## Model selection table
## (Int) Day_c Tmp Trt Day_c:Tmp Day_c:Trt Tmp:Trt Day_c:Tmp:Trt
## 2 -0.19640 0.13390
## 6 -0.46800 0.13520 +
## 130 -1.10300 0.07769
## 133 -1.30200 +
## 134 -1.37600 0.07881 +
## 129 -1.03300
## 4 -0.12510 0.13420 +
## 8 -0.39250 0.13550 + +
## 22 -0.48420 0.14720 + +
## 131 -0.96330 +
## 135 -1.22700 + +
## 132 -1.03000 0.07797 +
## 136 -1.29900 0.07910 + +
## 12 -0.19000 0.18070 + +
## 150 -1.39400 0.09210 + +
## 16 -0.45330 0.18080 + + +
## 40 -0.49370 0.13510 + + +
## 1 -0.11230
## 24 -0.40890 0.14810 + + +
## 5 -0.37700 +
## 140 -1.09700 0.12580 + +
## 144 -1.36200 0.12580 + + +
## 167 -1.33100 + + +
## 168 -1.39900 0.07873 + + +
## 152 -1.31700 0.09293 + + +
## 48 -0.55770 0.18070 + + + +
## 32 -0.46900 0.19260 + + + +
## 3 -0.04721 +
## 7 -0.30660 + +
## 56 -0.50850 0.14690 + + + +
## 176 -1.46400 0.12580 + + + +
## 160 -1.38000 0.13890 + + + +
## 184 -1.41500 0.09191 + + + +
## 39 -0.41510 + + +
## 64 -0.58660 0.19820 + + + + +
## 128 -0.45210 0.09462 + + + + + +
## 192 -1.49600 0.14470 + + + + +
## 256 -1.35500 0.03639 + + + + + +
## off(log(dys)) df logLik AICc delta weight
## 2 3 -56.855 120.3 0.00 0.146
## 6 4 -55.684 120.3 0.05 0.142
## 130 + 3 -57.550 121.7 1.39 0.073
## 133 + 3 -57.550 121.7 1.39 0.073
## 134 + 4 -56.363 121.7 1.41 0.072
## 129 + 2 -58.705 121.7 1.41 0.072
## 4 4 -56.771 122.5 2.23 0.048
## 8 5 -55.567 122.6 2.33 0.046
## 22 5 -55.669 122.8 2.53 0.041
## 131 + 3 -58.625 123.8 3.54 0.025
## 135 + 4 -57.437 123.8 3.56 0.025
## 132 + 4 -57.462 123.9 3.61 0.024
## 136 + 5 -56.241 123.9 3.68 0.023
## 12 5 -56.303 124.1 3.80 0.022
## 150 + 5 -56.344 124.2 3.88 0.021
## 16 6 -55.123 124.3 4.08 0.019
## 40 6 -55.423 124.9 4.68 0.014
## 1 2 -60.459 125.2 4.92 0.012
## 24 6 -55.550 125.2 4.93 0.012
## 5 3 -59.348 125.3 4.99 0.012
## 140 + 5 -56.987 125.4 5.17 0.011
## 144 + 6 -55.788 125.7 5.41 0.010
## 167 + 5 -57.287 126.0 5.77 0.008
## 168 + 6 -56.102 126.3 6.04 0.007
## 152 + 6 -56.221 126.5 6.27 0.006
## 48 7 -54.971 126.8 6.54 0.006
## 32 7 -55.107 127.1 6.82 0.005
## 3 3 -60.389 127.3 7.07 0.004
## 7 4 -59.246 127.4 7.18 0.004
## 56 7 -55.407 127.7 7.42 0.004
## 176 + 7 -55.642 128.2 7.89 0.003
## 160 + 7 -55.770 128.4 8.14 0.002
## 184 + 7 -56.084 129.0 8.77 0.002
## 39 5 -59.081 129.6 9.36 0.001
## 64 8 -54.941 129.7 9.40 0.001
## 128 9 -53.773 130.4 10.14 0.001
## 192 + 8 -55.609 131.0 10.74 0.001
## 256 + 9 -54.380 131.6 11.36 0.001
## Models ranked by AICc(x)
## Random terms (all models):
## 1 | unit

# Select models with ΔAICc <= 2 (or up to 95% cumulative weight)
avgset_mortality_pois <- subset(dredge_mortality_pois, delta <= 2)
#if (nrow(avgset_mortality_pois) == 0) {
# avgset_mortality_pois <- dredge_mortality_pois[cumsum(dredge_mortality_pois$weight) <= 0.95, ]}

# Model averaging
avg_mortality_pois <- model.avg(avgset_mortality_pois)
summary(avg_mortality_pois)

##
## Call:
## model.avg(object = avgset_mortality_pois)
##
## Component model call:
## glmer(formula = deadd ~ <6 unique rhs>, data = mortality, family =
## poisson(link = "log"))
##
## Component models:
## df logLik AICc delta weight
## 1 3 -56.86 120.27 0.00 0.25
## 12 4 -55.68 120.32 0.05 0.25
## 13 3 -57.55 121.66 1.39 0.13
## 23 3 -57.55 121.66 1.39 0.13
## 123 4 -56.36 121.68 1.41 0.12
## 3 2 -58.71 121.68 1.41 0.12
##
## Term codes:
## Day_c Treatment offset(log(days))
## 1 2 3
##
## Model-averaged coefficients:
## (full average)
## Estimate Std. Error Adjusted SE z value Pr(>|z|)
## (Intercept) -0.76831 0.51448 0.51757 1.484 0.138
## Day_c 0.08671 0.07072 0.07152 1.212 0.225
## Treatment6PPD-Q 0.26135 0.35122 0.35570 0.735 0.462
##
## (conditional average)
## Estimate Std. Error Adjusted SE z value Pr(>|z|)
## (Intercept) -0.76831 0.51448 0.51757 1.484 0.1377
## Day_c 0.11569 0.05761 0.05892 1.964 0.0496 *
## Treatment6PPD-Q 0.52597 0.33027 0.33977 1.548 0.1216
## ---
## Signif. codes: 0 '***' 0.001 '**' 0.01 '*' 0.05 '.' 0.1 ' ' 1

#1.7. Diagnostics GLMM Poisson distribution

mod_avgmortality_pois_RE <- glmer(deadd ~ Treatment + Day_c +
 offset(log(days)) +
 (1 | unit),
 data = mortality,
 family = poisson(link = "log")
)

summary(mod_avgmortality_pois_RE)

## Generalized linear mixed model fit by maximum likelihood (Laplace
## Approximation) [glmerMod]
## Family: poisson ( log )
## Formula: deadd ~ Treatment + Day_c + offset(log(days)) + (1 | unit)
## Data: mortality
##
## AIC BIC logLik -2*log(L) df.resid
## 120.7 128.1 -56.4 112.7 43
##
## Scaled residuals:
## Min 1Q Median 3Q Max
## -1.3101 -0.6372 -0.3049 0.3643 2.7232
##
## Random effects:
## Groups Name Variance Std.Dev.
## unit (Intercept) 0.03132 0.177
## Number of obs: 47, groups: unit, 12
##
## Fixed effects:
## Estimate Std. Error z value Pr(>|z|)
## (Intercept) -1.37621 0.27208 -5.058 4.23e-07 ***
## Treatment6PPD-Q 0.52941 0.33004 1.604 0.109
## Day_c 0.07881 0.05187 1.519 0.129
## ---
## Signif. codes: 0 '***' 0.001 '**' 0.01 '*' 0.05 '.' 0.1 ' ' 1
##
## Correlation of Fixed Effects:
## (Intr) T6PPD-
## Trtmn6PPD-Q -0.751
## Day_c -0.245 0.014

check_model(mod_avgmortality_pois_RE)
MuMIn::AICc(mod_avgmortality_pois_RE)

## [1] 121.6779

performance::r2(mod_avgmortality_pois_RE)

## # R2 for Mixed Models
##
## Conditional R2: 0.110
## Marginal R2: 0.088

res <- simulateResiduals(mod_avgmortality_pois_RE)
plot(res)
testDispersion(res)

##
## DHARMa nonparametric dispersion test via sd of residuals fitted vs.
## simulated
##
## data: simulationOutput
## dispersion = 1.0876, p-value = 0.664
## alternative hypothesis: two.sided

testZeroInflation(res) #there is zero inflation

##
## DHARMa zero-inflation test via comparison to expected zeros with
## simulation under H0 = fitted model
##
## data: simulationOutput
## ratioObsSim = 0.88132, p-value = 0.592
## alternative hypothesis: two.sided

testUniformity(res)

##
## Exact one-sample Kolmogorov-Smirnov test
##
## data: simulationOutput$scaledResiduals
## D = 0.066993, p-value = 0.9749
## alternative hypothesis: two-sided

# 2. ANALYSIS OF CLUTCHES

## 2.1 Load data

clutch <- read.csv("clutch.csv", sep = ";", header = TRUE)


summary (clutch)

## replicate Treatment Temperature clutches Day
## Min. :1 Length:36 Min. :15.0 Min. :0.000 Min. : 4
## 1st Qu.:1 Class :character 1st Qu.:15.0 1st Qu.:0.000 1st Qu.: 4
## Median :2 Mode :character Median :17.5 Median :1.000 Median : 7
## Mean :2 Mean :17.5 Mean :1.472 Mean : 7
## 3rd Qu.:3 3rd Qu.:20.0 3rd Qu.:2.000 3rd Qu.:10
## Max. :3 Max. :20.0 Max. :5.000 Max. :10
## snails.alive logclutches clutchsnaliveday logclutchsnalive
## Min. : 4.000 Min. :0.0000 Min. :0.00000 Min. :0.00000
## 1st Qu.: 8.000 1st Qu.:0.0000 1st Qu.:0.00000 1st Qu.:0.00000
## Median :10.000 Median :0.3010 Median :0.04167 Median :0.01773
## Mean : 9.139 Mean :0.3197 Mean :0.06441 Mean :0.02628
## 3rd Qu.:10.000 3rd Qu.:0.4771 3rd Qu.:0.10000 3rd Qu.:0.04139
## Max. :12.000 Max. :0.7782 Max. :0.28571 Max. :0.10914

clutch$replicate <- factor(clutch$replicate)
clutch$Treatment <- factor(clutch$Treatment, levels=c("CTL", "6PPD-Q"))
clutch$Temperature <- factor(clutch$Temperature)
clutch$Day_c <- scale(clutch$Day, center=TRUE, scale=FALSE)
clutch$unit <- interaction(clutch$Treatment, clutch$Temperature, clutch$replicate)
summary(clutch)

## replicate Treatment Temperature clutches Day
## 1:12 CTL :18 15:18 Min. :0.000 Min. : 4
## 2:12 6PPD-Q:18 20:18 1st Qu.:0.000 1st Qu.: 4
## 3:12 Median :1.000 Median : 7
## Mean :1.472 Mean : 7
## 3rd Qu.:2.000 3rd Qu.:10
## Max. :5.000 Max. :10
##
## snails.alive logclutches clutchsnaliveday logclutchsnalive
## Min. : 4.000 Min. :0.0000 Min. :0.00000 Min. :0.00000
## 1st Qu.: 8.000 1st Qu.:0.0000 1st Qu.:0.00000 1st Qu.:0.00000
## Median :10.000 Median :0.3010 Median :0.04167 Median :0.01773
## Mean : 9.139 Mean :0.3197 Mean :0.06441 Mean :0.02628
## 3rd Qu.:10.000 3rd Qu.:0.4771 3rd Qu.:0.10000 3rd Qu.:0.04139
## Max. :12.000 Max. :0.7782 Max. :0.28571 Max. :0.10914
##
## Day_c.V1 unit
## Min. :-3 CTL.15.1 : 3
## 1st Qu.:-3 6PPD-Q.15.1: 3
## Median : 0 CTL.20.1 : 3
## Mean : 0 6PPD-Q.20.1: 3
## 3rd Qu.: 3 CTL.15.2 : 3
## Max. : 3 6PPD-Q.15.2: 3
## (Other) :18

#Distribution clutches
hist(clutch$clutchsnaliveday,
 col = "grey",
 main = "Distribution of cluches/snail/day",
 xlab = "clutches/snail/day")


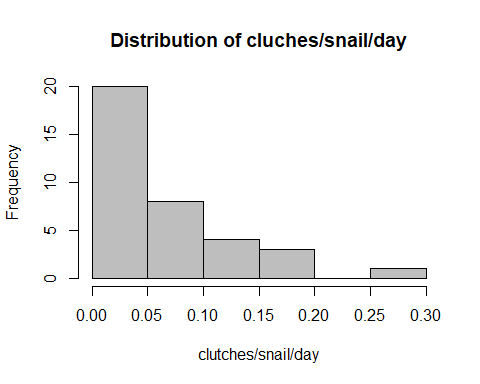


## 2.2. GLMM with random effects

#Poisson with offset (snails.alive)

mod_clutch_poisRE <- glmer(
 clutches ~ Treatment * Temperature * Day_c +
 offset(log(snails.alive)) +
 (1 | unit),
 data = clutch,
 family = poisson(link="log")
)
summary(mod_clutch_poisRE)

## Generalized linear mixed model fit by maximum likelihood (Laplace
## Approximation) [glmerMod]
## Family: poisson ( log )
## Formula:
## clutches ~ Treatment * Temperature * Day_c + offset(log(snails.alive)) +
## (1 | unit)
## Data: clutch
##
## AIC BIC logLik -2*log(L) df.resid
## 123.2 137.4 -52.6 105.2 27
##
## Scaled residuals:
## Min 1Q Median 3Q Max
## -1.4398 -0.8083 -0.3691 0.5964 2.7329
##
## Random effects:
## Groups Name Variance Std.Dev.
## unit (Intercept) 0.0243 0.1559
## Number of obs: 36, groups: unit, 12
##
## Fixed effects:
## Estimate Std. Error z value Pr(>|z|)
## (Intercept) -1.7998693 0.2774795 -6.486 8.79e-11 ***
## Treatment6PPD-Q -0.3705397 0.4437324 -0.835 0.4037
## Temperature20 0.4692547 0.3639979 1.289 0.1973
## Day_c 0.0001267 0.1057026 0.001 0.9990
## Treatment6PPD-Q:Temperature20 -1.0034663 0.7173969 -1.399 0.1619
## Treatment6PPD-Q:Day_c 0.1455546 0.1754971 0.829 0.4069
## Temperature20:Day_c 0.0923017 0.1384079 0.667 0.5048
## Treatment6PPD-Q:Temperature20:Day_c -0.5871897 0.2741031 -2.142 0.0322 *
## ---
## Signif. codes: 0 '***' 0.001 '**' 0.01 '*' 0.05 '.' 0.1 ' ' 1
##
## Correlation of Fixed Effects:
## (Intr) Tr6PPD-Q Tmpr20 Day_c Tr6PPD-Q:T20 T6PPD-Q:D T20:D_
## Trtmn6PPD-Q -0.607
## Temperatr20 -0.753 0.464
## Day_c 0.075 -0.048 -0.058
## Tr6PPD-Q:T20 0.380 -0.618 -0.507 0.029
## Tr6PPD-Q:D_ -0.047 -0.055 0.035 -0.602 0.034
## Tmprtr20:D_ -0.060 0.036 0.001 -0.764 0.000 0.460
## T6PPD-Q:T20: 0.029 0.035 0.000 0.386 0.314 -0.640 -0.505

## Overdispersion test (Poisson)

overdisp_fun <- function(model){
 rdf <- df.residual(model)
 rp <- residuals(model, type="pearson")
 sum(rp^2)/rdf
}

overdisp_fun(mod_clutch_poisRE)

## [1] 1.35039

#- If > 1.2 → *Negative Binomial*

## Full NB model with random effect

mod_clutch_RE <- glmmTMB(
 clutches ~ Treatment * Temperature * Day_c +
 offset(log(snails.alive)) +
 (1 | unit),
 family = nbinom2(),
 data = clutch
)
summary(mod_clutch_RE)

## Family: nbinom2 ( log )
## Formula:
## clutches ~ Treatment * Temperature * Day_c + offset(log(snails.alive)) +
## (1 | unit)
## Data: clutch
##
## AIC BIC logLik -2*log(L) df.resid
## 125.2 141.0 -52.6 105.2 26
##
## Random effects:
##
## Conditional model:
## Groups Name Variance Std.Dev.
## unit (Intercept) 0.02432 0.1559
## Number of obs: 36, groups: unit, 12
##
## Dispersion parameter for nbinom2 family (): 6.73e+06
##
## Conditional model:
## Estimate Std. Error z value Pr(>|z|)
## (Intercept) -1.7999002 0.2776649 -6.482 9.04e-11 ***
## Treatment6PPD-Q -0.3705427 0.4439341 -0.835 0.4039
## Temperature20 0.4692755 0.3642237 1.288 0.1976
## Day_c 0.0001219 0.1057659 0.001 0.9991
## Treatment6PPD-Q:Temperature20 -1.0034844 0.7177098 -1.398 0.1621
## Treatment6PPD-Q:Day_c 0.1455621 0.1755776 0.829 0.4071
## Temperature20:Day_c 0.0923107 0.1384981 0.667 0.5051
## Treatment6PPD-Q:Temperature20:Day_c -0.5872143 0.2742258 -2.141 0.0322 *
## ---
## Signif. codes: 0 '***' 0.001 '**' 0.01 '*' 0.05 '.' 0.1 ' ' 1

## NB model without random effect

mod_clutch_noRE <- glmmTMB(
 clutches ~ Treatment * Temperature * Day_c +
 offset(log(snails.alive)),
 family = nbinom2(),
 data = clutch
)
summary(mod_clutch_noRE)

## Family: nbinom2 ( log )
## Formula:
## clutches ~ Treatment * Temperature * Day_c + offset(log(snails.alive))
## Data: clutch
##
## AIC BIC logLik -2*log(L) df.resid
## 123.2 137.5 -52.6 105.2 27
##
##
## Dispersion parameter for nbinom2 family (): 449
##
## Conditional model:
## Estimate Std. Error z value Pr(>|z|)
## (Intercept) -1.792e+00 2.597e-01 -6.899 5.25e-12 ***
## Treatment6PPD-Q -3.688e-01 4.256e-01 -0.867 0.3861
## Temperature20 4.659e-01 3.405e-01 1.369 0.1711
## Day_c -3.298e-05 1.060e-01 0.000 0.9998
## Treatment6PPD-Q:Temperature20 -1.002e+00 6.947e-01 -1.443 0.1491
## Treatment6PPD-Q:Day_c 1.452e-01 1.758e-01 0.826 0.4090
## Temperature20:Day_c 9.184e-02 1.389e-01 0.661 0.5085
## Treatment6PPD-Q:Temperature20:Day_c -5.873e-01 2.746e-01 -2.139 0.0325 *
## ---
## Signif. codes: 0 '***' 0.001 '**' 0.01 '*' 0.05 '.' 0.1 ' ' 1

## Compare models (RE vs noRE)

AIC(mod_clutch_RE, mod_clutch_noRE)

## df AIC
## mod_clutch_RE 10 125.1931
## mod_clutch_noRE 9 123.2349

anova(mod_clutch_RE, mod_clutch_noRE)

## Data: clutch
## Models:
## mod_clutch_noRE: clutches ~ Treatment * Temperature * Day_c + offset(log(snails.alive)), zi=~0, disp=~1
## mod_clutch_RE: clutches ~ Treatment * Temperature * Day_c + offset(log(snails.alive)) + , zi=~0, disp=~1
## mod_clutch_RE: (1 | unit), zi=~0, disp=~1
## Df AIC BIC logLik deviance Chisq Chi Df Pr(>Chisq)
## mod_clutch_noRE 9 123.23 137.49 -52.617 105.23
## mod_clutch_RE 10 125.19 141.03 -52.597 105.19 0.0418 1 0.8379

# LRT COMPARISON
anova(mod_clutch_RE, mod_clutch_noRE)

## Data: clutch
## Models:
## mod_clutch_noRE: clutches ~ Treatment * Temperature * Day_c + offset(log(snails.alive)), zi=~0, disp=~1
## mod_clutch_RE: clutches ~ Treatment * Temperature * Day_c + offset(log(snails.alive)) + , zi=~0, disp=~1
## mod_clutch_RE: (1 | unit), zi=~0, disp=~1
## Df AIC BIC logLik deviance Chisq Chi Df Pr(>Chisq)
## mod_clutch_noRE 9 123.23 137.49 -52.617 105.23
## mod_clutch_RE 10 125.19 141.03 -52.597 105.19 0.0418 1 0.8379

# AICc COMPARISON
AICc(mod_clutch_RE); AICc(mod_clutch_noRE)

## [1] 133.9931

## [1] 130.158

# SELECT BEST WITH AICc
mods <- list(RE = mod_clutch_RE, noRE = mod_clutch_noRE)
best_model_clutch <- mods[[ which.min(sapply(mods, AICc)) ]]

summary(best_model_clutch)

## Family: nbinom2 ( log )
## Formula:
## clutches ~ Treatment * Temperature * Day_c + offset(log(snails.alive))
## Data: clutch
##
## AIC BIC logLik -2*log(L) df.resid
## 123.2 137.5 -52.6 105.2 27
##
##
## Dispersion parameter for nbinom2 family (): 449
##
## Conditional model:
## Estimate Std. Error z value Pr(>|z|)
## (Intercept) -1.792e+00 2.597e-01 -6.899 5.25e-12 ***
## Treatment6PPD-Q -3.688e-01 4.256e-01 -0.867 0.3861
## Temperature20 4.659e-01 3.405e-01 1.369 0.1711
## Day_c -3.298e-05 1.060e-01 0.000 0.9998
## Treatment6PPD-Q:Temperature20 -1.002e+00 6.947e-01 -1.443 0.1491
## Treatment6PPD-Q:Day_c 1.452e-01 1.758e-01 0.826 0.4090
## Temperature20:Day_c 9.184e-02 1.389e-01 0.661 0.5085
## Treatment6PPD-Q:Temperature20:Day_c -5.873e-01 2.746e-01 -2.139 0.0325 *
## ---
## Signif. codes: 0 '***' 0.001 '**' 0.01 '*' 0.05 '.' 0.1 ' ' 1

## 2.3. GLMM with random effects model selection

options(na.action = "na.fail")
dredge_clutch <- dredge(mod_clutch_RE, rank = "AICc")
dredge_clutch

## Global model call: glmmTMB(formula = clutches ~ Treatment * Temperature * Day_c +
## offset(log(snails.alive)) + (1 | unit), data = clutch, family = nbinom2(),
## ziformula = ~0, dispformula = ~1)
## ---
## Model selection table
## cnd((Int)) dsp((Int)) cnd(Day_c) cnd(Tmp) cnd(Trt) cnd(Day_c:Tmp)
## 5 0.6931 + +
## 133 -1.5400 + +
## 7 0.5982 + + +
## 6 0.6919 + -0.0319900 +
## 1 0.3452 +
## 129 -1.8870 +
## 135 -1.6660 + + +
## 134 -1.5420 + 0.0103900 +
## 22 0.6926 + 0.0139900 +
## 39 0.5108 + + +
## 131 -2.0040 + +
## 3 0.2585 + +
## 2 0.3397 + -0.0296600
## 8 0.5970 + -0.0314200 + +
## 130 -1.8870 + 0.0168700
## 167 -1.7860 + + +
## 150 -1.5460 + 0.0528300 +
## 136 -1.6650 + 0.0090950 + +
## 24 0.6000 + 0.0134600 + +
## 4 0.2537 + -0.0291100 +
## 132 -2.0030 + 0.0164500 +
## 40 0.5078 + -0.0323500 + +
## 16 0.6012 + 0.0020920 + + +
## 168 -1.7850 + 0.0097290 + +
## 152 -1.6720 + 0.0516800 + +
## 144 -1.6590 + 0.0531500 + + +
## 56 0.5107 + 0.0130600 + +
## 140 -2.0000 + 0.0565100 + +
## 32 0.6053 + 0.0478700 + + +
## 12 0.2526 + -0.0004274 + +
## 48 0.5109 + 0.0013510 + + +
## 184 -1.7840 + 0.0507600 + +
## 176 -1.7830 + 0.0544500 + + +
## 160 -1.6600 + 0.0927200 + + +
## 64 0.5050 + 0.0579800 + + +
## 256 -1.8000 + 0.0001214 + + +
## 128 0.5075 + -0.0337100 + + +
## 192 -1.7910 + 0.1051000 + + +
## cnd(Day_c:Trt) cnd(Tmp:Trt) cnd(Day_c:Tmp:Trt) cnd(off(log(snl.alv))) df
## 5 4
## 133 + 4
## 7 5
## 6 5
## 1 3
## 129 + 3
## 135 + 5
## 134 + 5
## 22 + 6
## 39 + 6
## 131 + 4
## 3 4
## 2 4
## 8 6
## 130 + 4
## 167 + + 6
## 150 + + 6
## 136 + 6
## 24 + 7
## 4 5
## 132 + 5
## 40 + 7
## 16 7
## 168 + + 7
## 152 + + 7
## 144 + 7
## 56 + + 8
## 140 + 6
## 32 + 8
## 12 6
## 48 + 8
## 184 + + + 8
## 176 + + 8
## 160 + + 8
## 64 + + 9
## 256 + + + + 10
## 128 + + + 10
## 192 + + + 9
## logLik AICc delta weight
## 5 -55.835 121.0 0.00 0.250
## 133 -56.458 122.2 1.25 0.134
## 7 -55.659 123.3 2.36 0.077
## 6 -55.702 123.4 2.44 0.074
## 1 -58.426 123.6 2.64 0.067
## 129 -58.460 123.7 2.71 0.065
## 135 -56.136 124.3 3.31 0.048
## 134 -56.446 124.9 3.93 0.035
## 22 -55.165 125.2 4.27 0.030
## 39 -55.397 125.7 4.73 0.023
## 131 -58.260 125.8 4.85 0.022
## 3 -58.294 125.9 4.92 0.021
## 2 -58.320 125.9 4.97 0.021
## 8 -55.527 125.9 4.99 0.021
## 130 -58.424 126.1 5.18 0.019
## 167 -55.670 126.2 5.28 0.018
## 150 -56.020 126.9 5.98 0.013
## 136 -56.125 127.1 6.19 0.011
## 24 -54.993 128.0 7.02 0.007
## 4 -58.193 128.4 7.43 0.006
## 132 -58.226 128.5 7.49 0.006
## 40 -55.255 128.5 7.55 0.006
## 16 -55.406 128.8 7.85 0.005
## 168 -55.658 129.3 8.36 0.004
## 152 -55.700 129.4 8.44 0.004
## 144 -55.932 129.9 8.90 0.003
## 56 -54.704 130.7 9.78 0.002
## 140 -58.058 131.0 10.05 0.002
## 32 -54.864 131.1 10.10 0.002
## 12 -58.112 131.1 10.16 0.002
## 48 -55.129 131.6 10.63 0.001
## 184 -55.237 131.8 10.85 0.001
## 176 -55.445 132.2 11.26 0.001
## 160 -55.523 132.4 11.42 0.001
## 64 -54.511 133.9 12.98 0.000
## 256 -52.597 134.0 13.03 0.000
## 128 -52.685 134.2 13.21 0.000
## 192 -54.964 134.9 13.89 0.000
## Models ranked by AICc(x)
## Random terms (all models):
## cond(1 | unit)

# Select models with delta-AICc <= 2 and average

avgset_clutch <- subset(dredge_clutch, delta <= 2)

# Model averaging
avg_clutch <-model.avg(avgset_clutch)

summary(avg_clutch)

##
## Call:
## model.avg(object = avgset_clutch)
##
## Component model call:
## glmmTMB(formula = clutches ~ <2 unique rhs>, data = clutch, family =
## nbinom2(), ziformula = ~0, dispformula = ~1)
##
## Component models:
## df logLik AICc delta weight
## 1 4 -55.84 120.96 0.00 0.65
## 12 4 -56.46 122.21 1.25 0.35
##
## Term codes:
## cond(Treatment) cond(offset(log(snails.alive)))
## 1 2
##
## Model-averaged coefficients:
## (full average)
## Estimate Std. Error Adjusted SE z value Pr(>|z|)
## cond((Int)) -0.08647 1.08244 1.08386 0.080 0.9364
## cond(Treatment6PPD-Q) -0.73582 0.32503 0.33775 2.179 0.0294 *
##
## (conditional average)
## Estimate Std. Error Adjusted SE z value Pr(>|z|)
## cond((Int)) -0.08647 1.08244 1.08386 0.080 0.9364
## cond(Treatment6PPD-Q) -0.73582 0.32503 0.33775 2.179 0.0294 *
## ---
## Signif. codes: 0 '***' 0.001 '**' 0.01 '*' 0.05 '.' 0.1 ' ' 1

# sum of weights
sw_clutch <- sw(avgset_clutch)
sw_clutch

## cond(Treatment) cond(offset(log(snails.alive)))
## Sum of weights: 1.00 0.35
## N containing models: 2 1

## 2.4. Diagnostics

mod_avg_clutch_RE <- glmmTMB(
 clutches ~ Treatment +Temperature+Day_c + offset(log(snails.alive)) +
 (1 | unit),
 family = nbinom2(),
 data = clutch
)
performance::r2(mod_avg_clutch_RE)

## [1] NA

insight::get_variance_random(mod_avg_clutch_RE) # must be > 0 to calculate R²c

## var.random
## 1.617607e-08

insight::get_variance_fixed(mod_avg_clutch_RE) # fixed

## var.fixed
## 0.1448891

insight::get_variance_residual(mod_avg_clutch_RE) # residual

## var.residual
## 1.976358

performance::r2_nakagawa(mod_avg_clutch_RE)

## # R2 for Mixed Models
##
## Conditional R2: 0.068
## Marginal R2: 0.068

res_clutch <- simulateResiduals(mod_avg_clutch_RE)
plot(res_clutch)


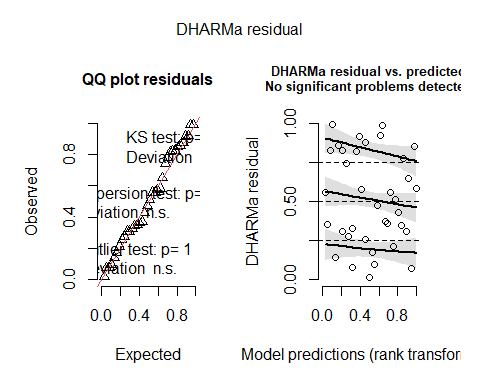


testDispersion(res_clutch)


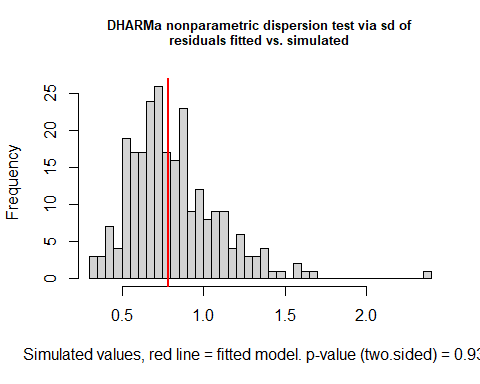


##
## DHARMa nonparametric dispersion test via sd of residuals fitted vs.
## simulated
##
## data: simulationOutput
## dispersion = 0.96122, p-value = 0.936
## alternative hypothesis: two.sided

testZeroInflation(res_clutch)


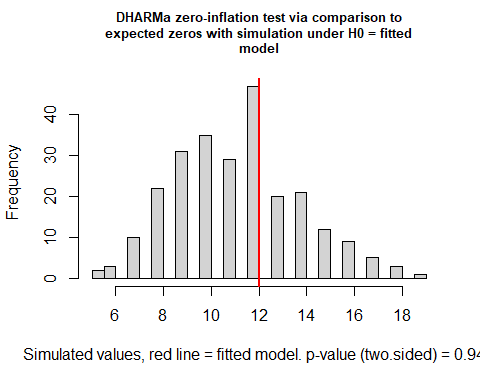


##
## DHARMa zero-inflation test via comparison to expected zeros with
## simulation under H0 = fitted model
##
## data: simulationOutput
## ratioObsSim = 1.0631, p-value = 0.944
## alternative hypothesis: two.sided

testUniformity(res_clutch) # KS test for uniformity


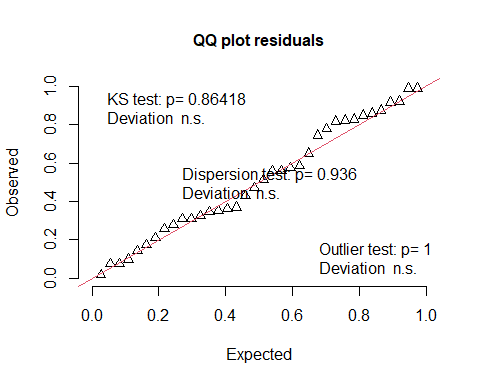


##
## Exact one-sample Kolmogorov-Smirnov test
##
## data: simulationOutput$scaledResiduals
## D = 0.095856, p-value = 0.8642
## alternative hypothesis: two-sided

# 3. ANALYSIS OF EGGS PER CLUTCH

## 3.1. Load data

eggs <- read.csv("eggs.csv", sep = ";", header = TRUE)
summary(eggs)

## replicate Treatment Temperature Day
## Min. :1.000 Length:54 Min. :15.00 Min. : 4.000
## 1st Qu.:1.000 Class :character 1st Qu.:15.00 1st Qu.: 4.000
## Median :2.000 Mode :character Median :20.00 Median : 7.000
## Mean :1.889 Mean :17.69 Mean : 6.778
## 3rd Qu.:2.750 3rd Qu.:20.00 3rd Qu.:10.000
## Max. :3.000 Max. :20.00 Max. :10.000
##
## eggsclutch logeggsclutch total.eggs
## Min. : 2.00 Min. :0.4771 Min. : 5.00
## 1st Qu.: 9.00 1st Qu.:1.0000 1st Qu.:11.25
## Median :13.00 Median :1.1461 Median :23.50
## Mean :14.02 Mean :1.1129 Mean :29.54
## 3rd Qu.:17.75 3rd Qu.:1.2729 3rd Qu.:41.00
## Max. :39.00 Max. :1.6021 Max. :88.00
## NA's :28

eggs$replicate <- factor(eggs$replicate)
eggs$Treatment <- factor(eggs$Treatment, levels = c("CTL","6PPD-Q"))
eggs$Temperature <- factor(eggs$Temperature)
eggs$unit <- interaction(eggs$Treatment, eggs$Temperature, eggs$replicate)
eggs$Day_c <- scale(eggs$Day, center=TRUE, scale=FALSE)

#Distribution clutches
hist(eggs$eggsclutch,
 col = "grey",
 main = "Distribution of eggs/clutch",
 xlab = "eggsclutch")


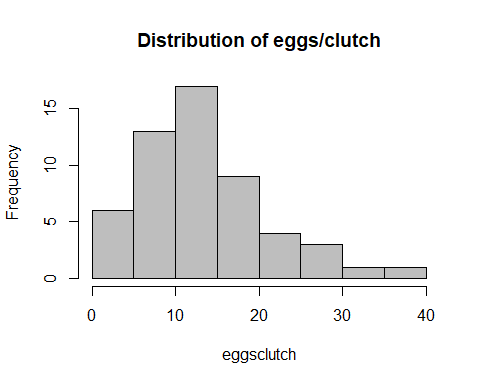


hist(eggs$logeggsclutch,
 col = "grey",
 main = "Distribution of eggs/clutch",
 xlab = "eggsclutch")


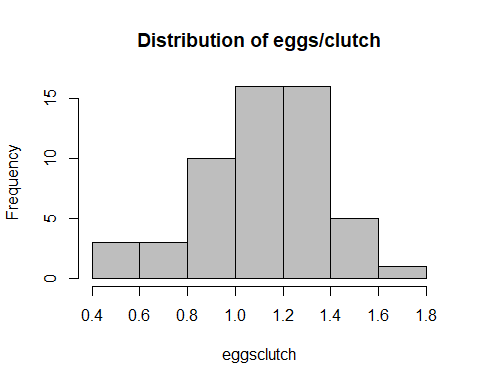


## 3.2. LMM with random effect (initial model)

mod_eggs_RE <- lmer(
 logeggsclutch ~ Treatment * Temperature * Day_c +
 (1 | unit),
 data = eggs,
 REML = FALSE
)
summary(mod_eggs_RE)

## Linear mixed model fit by maximum likelihood ['lmerMod']
## Formula: logeggsclutch ~ Treatment * Temperature * Day_c + (1 | unit)
## Data: eggs
##
## AIC BIC logLik -2*log(L) df.resid
## 11.5 31.4 4.3 -8.5 44
##
## Scaled residuals:
## Min 1Q Median 3Q Max
## -2.96507 -0.55823 0.05192 0.59317 2.07281
##
## Random effects:
## Groups Name Variance Std.Dev.
## unit (Intercept) 0.00000 0.0000
## Residual 0.05001 0.2236
## Number of obs: 54, groups: unit, 10
##
## Fixed effects:
## Estimate Std. Error t value
## (Intercept) 1.0926288 0.0577468 18.921
## Treatment6PPD-Q -0.0485233 0.0915526 -0.530
## Temperature20 0.0769957 0.0762038 1.010
## Day_c 0.0110771 0.0283110 0.391
## Treatment6PPD-Q:Temperature20 0.1318615 0.1587520 0.831
## Treatment6PPD-Q:Day_c 0.0624283 0.0415657 1.502
## Temperature20:Day_c -0.0004941 0.0339562 -0.015
## Treatment6PPD-Q:Temperature20:Day_c 0.0480619 0.0710143 0.677
##
## Correlation of Fixed Effects:
## (Intr) Tr6PPD-Q Tmpr20 Day_c Tr6PPD-Q:T20 T6PPD-Q:D T20:D_
## Trtmn6PPD-Q -0.631
## Temperatr20 -0.758 0.478
## Day_c -0.011 0.007 0.008
## Tr6PPD-Q:T20 0.364 -0.577 -0.480 -0.004
## Tr6PPD-Q:D_ 0.007 -0.059 -0.006 -0.681 0.034
## Tmprtr20:D_ 0.009 -0.006 -0.076 -0.834 0.036 0.568
## T6PPD-Q:T20: -0.004 0.034 0.036 0.399 0.399 -0.585 -0.478
## optimizer (nloptwrap) convergence code: 0 (OK)
## boundary (singular) fit: see help('isSingular')

## 3.3. LM without random effect

mod_eggs_noRE <- lm(
 logeggsclutch ~ Treatment * Temperature * Day_c,
 data = eggs
)
summary(mod_eggs_noRE)

##
## Call:
## lm(formula = logeggsclutch ~ Treatment * Temperature * Day_c,
## data = eggs)
##
## Residuals:
## Min 1Q Median 3Q Max
## -0.66311 -0.12484 0.01161 0.13266 0.46356
##
## Coefficients:
## Estimate Std. Error t value Pr(>|t|)
## (Intercept) 1.0926288 0.0625671 17.463 <2e-16 ***
## Treatment6PPD-Q -0.0485233 0.0991947 -0.489 0.627
## Temperature20 0.0769957 0.0825647 0.933 0.356
## Day_c 0.0110771 0.0306742 0.361 0.720
## Treatment6PPD-Q:Temperature20 0.1318615 0.1720034 0.767 0.447
## Treatment6PPD-Q:Day_c 0.0624283 0.0450353 1.386 0.172
## Temperature20:Day_c -0.0004941 0.0367906 -0.013 0.989
## Treatment6PPD-Q:Temperature20:Day_c 0.0480619 0.0769421 0.625 0.535
## ---
## Signif. codes: 0 '***' 0.001 '**' 0.01 '*' 0.05 '.' 0.1 ' ' 1
##
## Residual standard error: 0.2423 on 46 degrees of freedom
## Multiple R-squared: 0.2073, Adjusted R-squared: 0.08662
## F-statistic: 1.718 on 7 and 46 DF, p-value: 0.1283

## 3.4. Compare RE vs noRE

anova(mod_eggs_RE, mod_eggs_noRE)

## Data: eggs
## Models:
## mod_eggs_noRE: logeggsclutch ~ Treatment * Temperature * Day_c
## mod_eggs_RE: logeggsclutch ~ Treatment * Temperature * Day_c + (1 | unit)
## npar AIC BIC logLik -2*log(L) Chisq Df Pr(>Chisq)
## mod_eggs_noRE 9 9.4914 27.392 4.2543 -8.5086
## mod_eggs_RE 10 11.4914 31.381 4.2543 -8.5086 0 1 1

AICc(mod_eggs_RE)

## [1] 16.60769

AICc(mod_eggs_noRE)

## [1] 13.58232

# Select best model by AICc

mods_eggs <- list(RE = mod_eggs_RE, noRE = mod_eggs_noRE)
best_model_eggs <- mods_eggs[[ which.min(sapply(mods_eggs, AICc)) ]]
summary(best_model_eggs) #is best to keep the random effects to avoid pseudoreplication

##
## Call:
## lm(formula = logeggsclutch ~ Treatment * Temperature * Day_c,
## data = eggs)
##
## Residuals:
## Min 1Q Median 3Q Max
## -0.66311 -0.12484 0.01161 0.13266 0.46356
##
## Coefficients:
## Estimate Std. Error t value Pr(>|t|)
## (Intercept) 1.0926288 0.0625671 17.463 <2e-16 ***
## Treatment6PPD-Q -0.0485233 0.0991947 -0.489 0.627
## Temperature20 0.0769957 0.0825647 0.933 0.356
## Day_c 0.0110771 0.0306742 0.361 0.720
## Treatment6PPD-Q:Temperature20 0.1318615 0.1720034 0.767 0.447
## Treatment6PPD-Q:Day_c 0.0624283 0.0450353 1.386 0.172
## Temperature20:Day_c -0.0004941 0.0367906 -0.013 0.989
## Treatment6PPD-Q:Temperature20:Day_c 0.0480619 0.0769421 0.625 0.535
## ---
## Signif. codes: 0 '***' 0.001 '**' 0.01 '*' 0.05 '.' 0.1 ' ' 1
##
## Residual standard error: 0.2423 on 46 degrees of freedom
## Multiple R-squared: 0.2073, Adjusted R-squared: 0.08662
## F-statistic: 1.718 on 7 and 46 DF, p-value: 0.1283

## 3.5. LMM with random effects model selection

options(na.action = "na.fail")
dredge_eggs <- dredge(mod_eggs_RE, rank = "AICc")
dredge_eggs

## Global model call: lmer(formula = logeggsclutch ~ Treatment * Temperature * Day_c +
## (1 | unit), data = eggs, REML = FALSE)
## ---
## Model selection table
## (Int) Day_c Tmp Trt Day_c:Tmp Day_c:Trt Tmp:Trt Day_c:Tmp:Trt df logLik
## 2 1.113 0.031460 4 0.474
## 22 1.137 0.012310 + + 6 2.664
## 24 1.080 0.010280 + + + 7 3.824
## 4 1.077 0.032030 + 5 1.010
## 6 1.132 0.029400 + 5 0.801
## 1 1.111 3 -1.955
## 56 1.093 0.010730 + + + + 8 3.965
## 8 1.096 0.030250 + + 6 1.238
## 5 1.141 + 4 -1.341
## 32 1.079 0.006515 + + + + 8 3.842
## 12 1.076 0.039620 + + 6 1.107
## 3 1.080 + 4 -1.610
## 7 1.111 + + 5 -1.051
## 16 1.098 0.040190 + + + 7 1.410
## 40 1.092 0.029790 + + + 7 1.248
## 64 1.093 0.003438 + + + + + 9 4.026
## 39 1.093 + + + 6 -0.856
## 48 1.092 0.040040 + + + + 8 1.437
## 128 1.093 0.011080 + + + + + + 10 4.254
## AICc delta weight
## 2 7.9 0.00 0.208
## 22 8.5 0.59 0.155
## 24 8.8 0.92 0.131
## 4 9.2 1.36 0.105
## 6 9.6 1.78 0.085
## 1 10.4 2.52 0.059
## 56 11.3 3.40 0.038
## 8 11.3 3.44 0.037
## 5 11.5 3.63 0.034
## 32 11.5 3.65 0.034
## 12 11.6 3.70 0.033
## 3 12.0 4.17 0.026
## 7 13.4 5.48 0.013
## 16 13.6 5.75 0.012
## 40 13.9 6.07 0.010
## 64 14.0 6.17 0.010
## 39 15.5 7.63 0.005
## 48 16.3 8.46 0.003
## 128 16.6 8.74 0.003
## Models ranked by AICc(x)
## Random terms (all models):
## 1 | unit

avgset_eggs <- subset(dredge_eggs, delta <= 2)

# (Si no n'hi hagués cap o són massa pocs, usa el 95% del pes acumulat)
#if (nrow(avgset_eggs) == 0) {
 # avgset_eggs <- dredge_eggs[cumsum(dredge_eggs$weight) <= 0.95, ]}

# Model averaging
avg_eggs <- model.avg(avgset_eggs)

summary(avg_eggs)

##
## Call:
## model.avg(object = avgset_eggs)
##
## Component model call:
## lmer(formula = logeggsclutch ~ <5 unique rhs>, data = eggs, REML =
## FALSE)
##
## Component models:
## df logLik AICc delta weight
## 1 4 0.47 7.87 0.00 0.30
## 134 6 2.66 8.46 0.59 0.23
## 1234 7 3.82 8.79 0.92 0.19
## 12 5 1.01 9.23 1.36 0.15
## 13 5 0.80 9.65 1.78 0.12
##
## Term codes:
## Day_c Temperature Treatment Day_c:Treatment
## 1 2 3 4
##
## Model-averaged coefficients:
## (full average)
## Estimate Std. Error Adjusted SE z value Pr(>|z|)
## (Intercept) 1.10879 0.04814 0.04908 22.593 <2e-16 ***
## Day_c 0.02290 0.01767 0.01798 1.274 0.203
## Treatment6PPD-Q -0.01891 0.05451 0.05573 0.339 0.734
## Day_c:Treatment6PPD-Q 0.02642 0.03680 0.03706 0.713 0.476
## Temperature20 0.02937 0.05619 0.05686 0.517 0.605
##
## (conditional average)
## Estimate Std. Error Adjusted SE z value Pr(>|z|)
## (Intercept) 1.10879 0.04814 0.04908 22.593 <2e-16 ***
## Day_c 0.02290 0.01767 0.01798 1.274 0.2028
## Treatment6PPD-Q -0.03486 0.07015 0.07189 0.485 0.6278
## Day_c:Treatment6PPD-Q 0.06324 0.03020 0.03096 2.042 0.0411 *
## Temperature20 0.08498 0.06640 0.06803 1.249 0.2116
## ---
## Signif. codes: 0 '***' 0.001 '**' 0.01 '*' 0.05 '.' 0.1 ' ' 1

## 3.6. Diagnosis model with random effectes (lmer)

summary(clutch)

## replicate Treatment Temperature clutches Day
## 1:12 CTL :18 15:18 Min. :0.000 Min. : 4
## 2:12 6PPD-Q:18 20:18 1st Qu.:0.000 1st Qu.: 4
## 3:12 Median :1.000 Median : 7
## Mean :1.472 Mean : 7
## 3rd Qu.:2.000 3rd Qu.:10
## Max. :5.000 Max. :10
##
## snails.alive logclutches clutchsnaliveday logclutchsnalive
## Min. : 4.000 Min. :0.0000 Min. :0.00000 Min. :0.00000
## 1st Qu.: 8.000 1st Qu.:0.0000 1st Qu.:0.00000 1st Qu.:0.00000
## Median :10.000 Median :0.3010 Median :0.04167 Median :0.01773
## Mean : 9.139 Mean :0.3197 Mean :0.06441 Mean :0.02628
## 3rd Qu.:10.000 3rd Qu.:0.4771 3rd Qu.:0.10000 3rd Qu.:0.04139
## Max. :12.000 Max. :0.7782 Max. :0.28571 Max. :0.10914
##
## Day_c.V1 unit
## Min. :-3 CTL.15.1 : 3
## 1st Qu.:-3 6PPD-Q.15.1: 3
## Median : 0 CTL.20.1 : 3
## Mean : 0 6PPD-Q.20.1: 3
## 3rd Qu.: 3 CTL.15.2 : 3
## Max. : 3 6PPD-Q.15.2: 3
## (Other) :18

mod_avg_eggs_RE <- lmer(
 logeggsclutch ~ Treatment * Day_c + Temperature +
 (1 | unit),
 data = eggs,
 REML = FALSE
)
check_model(mod_avg_eggs_RE)
MuMIn::AICc(mod_avg_eggs_RE)

## [1] 8.78618

performance::r2(mod_avg_eggs_RE)

## Random effect variances not available. Returned R2 does not account for random effects.

## # R2 for Mixed Models
##
## Conditional R2: NA
## Marginal R2: 0.197

insight::get_variance_random(mod_avg_eggs_RE)

## NULL

insight::get_variance_fixed(mod_avg_eggs_RE)

## var.fixed
## 0.01250429

performance::r2_nakagawa(mod_avg_eggs_RE)

## Random effect variances not available. Returned R2 does not account for random effects.

## # R2 for Mixed Models
##
## Conditional R2: NA
## Marginal R2: 0.197

nlevels(eggs$unit); table(eggs$unit)

## [1] 12

##
## CTL.15.1 6PPD-Q.15.1 CTL.20.1 6PPD-Q.20.1 CTL.15.2 6PPD-Q.15.2
## 5 5 5 5 7 0
## CTL.20.2 6PPD-Q.20.2 CTL.15.3 6PPD-Q.15.3 CTL.20.3 6PPD-Q.20.3
## 10 3 3 5 6 0

lme4::VarCorr(mod_avg_eggs_RE)

## Groups Name Std.Dev.
## unit (Intercept) 0.00000
## Residual 0.22543

lme4::isSingular(mod_avg_eggs_RE, tol = 1e-5)

## [1] TRUE

insight::get_variance_random(mod_avg_eggs_RE)

## NULL

insight::get_variance_fixed(mod_avg_eggs_RE)

## var.fixed
## 0.01250429

mod_avg_eggs_LM <- lm(logeggsclutch ~ Treatment*Day_c + Temperature, data = eggs)
summary(mod_avg_eggs_LM) #estimates and significance very similar to the LMER

##
## Call:
## lm(formula = logeggsclutch ~ Treatment * Day_c + Temperature,
## data = eggs)
##
## Residuals:
## Min 1Q Median 3Q Max
## -0.67320 -0.13033 0.02427 0.12680 0.44100
##
## Coefficients:
## Estimate Std. Error t value Pr(>|t|)
## (Intercept) 1.07990 0.05565 19.407 <2e-16 ***
## Treatment6PPD-Q -0.01807 0.07142 -0.253 0.8013
## Day_c 0.01028 0.01652 0.622 0.5366
## Temperature20 0.09898 0.06746 1.467 0.1487
## Treatment6PPD-Q:Day_c 0.06949 0.03131 2.219 0.0311 *
## ---
## Signif. codes: 0 '***' 0.001 '**' 0.01 '*' 0.05 '.' 0.1 ' ' 1
##
## Residual standard error: 0.2366 on 49 degrees of freedom
## Multiple R-squared: 0.1945, Adjusted R-squared: 0.1288
## F-statistic: 2.958 on 4 and 49 DF, p-value: 0.02878

MuMIn::AICc(mod_avg_eggs_RE)

## [1] 8.78618

MuMIn::AICc(mod_avg_eggs_LM)

## [1] 6.138632

performance::r2(mod_avg_eggs_LM)

## # R2 for Linear Regression
## R2: 0.195
## adj. R2: 0.129

res <- simulateResiduals(mod_avg_eggs_RE)
plot(res)
testDispersion(res)

##
## DHARMa nonparametric dispersion test via sd of residuals fitted vs.
## simulated
##
## data: simulationOutput
## dispersion = 1.0155, p-value = 0.944
## alternative hypothesis: two.sided

testZeroInflation(res)

##
## DHARMa zero-inflation test via comparison to expected zeros with
## simulation under H0 = fitted model
##
## data: simulationOutput
## ratioObsSim = NaN, p-value = 1
## alternative hypothesis: two.sided

testUniformity(res)

##
## Asymptotic one-sample Kolmogorov-Smirnov test
##
## data: simulationOutput$scaledResiduals
## D = 0.071556, p-value = 0.945
## alternative hypothesis: two-sided

# 4. ANALYSIS OF TOTAL EGGS PER SNAIL PER DAY

## 4.1. Load data

eggssnail <- read.csv("eggssnail.csv", sep = ";", header = TRUE)
summary(eggssnail)

## replicate Treatment Temperature alive Day
## Min. :1 Length:36 Min. :15.0 Min. : 4.000 Min. : 4
## 1st Qu.:1 Class :character 1st Qu.:15.0 1st Qu.: 8.000 1st Qu.: 4
## Median :2 Mode :character Median :17.5 Median :10.000 Median : 7
## Mean :2 Mean :17.5 Mean : 9.139 Mean : 7
## 3rd Qu.:3 3rd Qu.:20.0 3rd Qu.:10.000 3rd Qu.:10
## Max. :3 Max. :20.0 Max. :12.000 Max. :10
## total.eggs days toteggssnailaliveday
## Min. : 0.00 Min. :2.000 Min. :0.0000
## 1st Qu.: 0.00 1st Qu.:2.000 1st Qu.:0.0000
## Median :13.50 Median :3.000 Median :0.5185
## Mean :21.03 Mean :2.667 Mean :0.8942
## 3rd Qu.:31.25 3rd Qu.:3.000 3rd Qu.:1.4139
## Max. :83.00 Max. :3.000 Max. :3.5714

eggssnail$replicate <- factor(eggssnail$replicate)
eggssnail$Treatment <- factor(eggssnail$Treatment, levels=c("CTL", "6PPD-Q"))
eggssnail$Temperature <- factor(eggssnail$Temperature)
eggssnail$unit <- interaction(eggssnail$Treatment, eggssnail$Temperature, eggssnail$replicate)
eggssnail$Day <- factor(eggssnail$Day)

 #Center Day if needed (optional)
eggssnail$Day_c <- scale(as.numeric(as.character(eggssnail$Day)), center = TRUE, scale = FALSE)
summary(eggssnail)

## replicate Treatment Temperature alive Day total.eggs
## 1:12 CTL :18 15:18 Min. : 4.000 4 :12 Min. : 0.00
## 2:12 6PPD-Q:18 20:18 1st Qu.: 8.000 7 :12 1st Qu.: 0.00
## 3:12 Median :10.000 10:12 Median :13.50
## Mean : 9.139 Mean :21.03
## 3rd Qu.:10.000 3rd Qu.:31.25
## Max. :12.000 Max. :83.00
##
## days toteggssnailaliveday unit Day_c.V1
## Min. :2.000 Min. :0.0000 CTL.15.1 : 3 Min. :-3
## 1st Qu.:2.000 1st Qu.:0.0000 6PPD-Q.15.1: 3 1st Qu.:-3
## Median :3.000 Median :0.5185 CTL.20.1 : 3 Median : 0
## Mean :2.667 Mean :0.8942 6PPD-Q.20.1: 3 Mean : 0
## 3rd Qu.:3.000 3rd Qu.:1.4139 CTL.15.2 : 3 3rd Qu.: 3
## Max. :3.000 Max. :3.5714 6PPD-Q.15.2: 3 Max. : 3
## (Other) :18

hist(eggssnail$toteggssnailaliveday,
 col = "grey",
 main = "Distribution of eggs/snail/day",
 xlab = "toteggssnailaliveday")


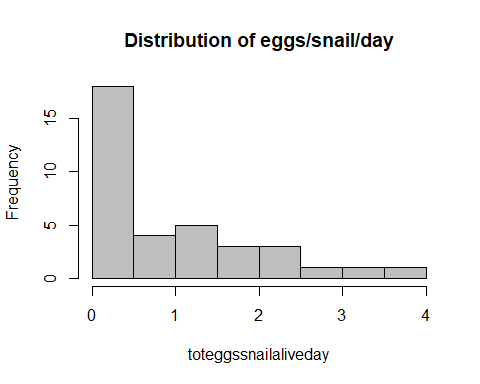


## 4.2. GLMM Poisson with random effect (initial model)

names(eggssnail)

## [1] "replicate" "Treatment" "Temperature"
## [4] "alive" "Day" "total.eggs"
## [7] "days" "toteggssnailaliveday" "unit"
## [10] "Day_c"

mod_eggssnail_pois_RE <- glmer(total.eggs ~ Treatment * Temperature * Day_c +
 offset(log(alive)) + offset(log(days)) +
 (1 | unit),
 data = eggssnail,
 family = poisson(link = "log")
)
summary(mod_eggssnail_pois_RE)

## Generalized linear mixed model fit by maximum likelihood (Laplace
## Approximation) [glmerMod]
## Family: poisson ( log )
## Formula: total.eggs ~ Treatment * Temperature * Day_c + offset(log(alive)) +
## offset(log(days)) + (1 | unit)
## Data: eggssnail
##
## AIC BIC logLik -2*log(L) df.resid
## 645.8 660.0 -313.9 627.8 27
##
## Scaled residuals:
## Min 1Q Median 3Q Max
## -5.1685 -2.4051 -0.6626 1.1421 7.8061
##
## Random effects:
## Groups Name Variance Std.Dev.
## unit (Intercept) 2.09 1.446
## Number of obs: 36, groups: unit, 12
##
## Fixed effects:
## Estimate Std. Error z value Pr(>|z|)
## (Intercept) -0.29095 0.83827 -0.347 0.7285
## Treatment6PPD-Q -1.29621 1.22288 -1.060 0.2892
## Temperature20 0.74085 1.18437 0.626 0.5316
## Day_c -0.04171 0.03062 -1.362 0.1732
## Treatment6PPD-Q:Temperature20 -1.00095 1.71677 -0.583 0.5599
## Treatment6PPD-Q:Day_c 0.24269 0.05096 4.762 1.92e-06 ***
## Temperature20:Day_c 0.08447 0.03851 2.193 0.0283 *
## Treatment6PPD-Q:Temperature20:Day_c -0.59920 0.07314 -8.193 2.55e-16 ***
## ---
## Signif. codes: 0 '***' 0.001 '**' 0.01 '*' 0.05 '.' 0.1 ' ' 1
##
## Correlation of Fixed Effects:
## (Intr) Tr6PPD-Q Tmpr20 Day_c Tr6PPD-Q:T20 T6PPD-Q:D T20:D_
## Trtmn6PPD-Q -0.685
## Temperatr20 -0.708 0.485
## Day_c 0.002 -0.001 -0.001
## Tr6PPD-Q:T20 0.488 -0.701 -0.690 0.001
## Tr6PPD-Q:D_ -0.001 -0.023 0.001 -0.601 0.016
## Tmprtr20:D_ -0.001 0.001 -0.003 -0.795 0.002 0.478
## T6PPD-Q:T20: 0.001 0.016 0.002 0.419 0.012 -0.697 -0.527

performance::r2(mod_eggssnail_pois_RE)

## # R2 for Mixed Models
##
## Conditional R2: 0.983
## Marginal R2: 0.345

## 4.3. GLM without random effect

mod_eggsnail_pois_noRE <- glm(
 total.eggs ~ Treatment * Temperature * Day_c +
 offset(log(alive)) + offset(log(days)),
 data = eggssnail,
 family = poisson(link = "log")
)
summary(mod_eggsnail_pois_noRE)

##
## Call:
## glm(formula = total.eggs ~ Treatment * Temperature * Day_c +
## offset(log(alive)) + offset(log(days)), family = poisson(link = "log"),
## data = eggssnail)
##
## Coefficients:
## Estimate Std. Error z value Pr(>|z|)
## (Intercept) -0.21799 0.07256 -3.004 0.00266 **
## Treatment6PPD-Q -0.34889 0.12094 -2.885 0.00392 **
## Temperature20 0.68018 0.09137 7.444 9.76e-14 ***
## Day_c -0.04264 0.03074 -1.387 0.16546
## Treatment6PPD-Q:Temperature20 -1.00818 0.17780 -5.670 1.43e-08 ***
## Treatment6PPD-Q:Day_c 0.24688 0.05108 4.833 1.35e-06 ***
## Temperature20:Day_c 0.08420 0.03862 2.180 0.02924 *
## Treatment6PPD-Q:Temperature20:Day_c -0.61666 0.07337 -8.404 < 2e-16 ***
## ---
## Signif. codes: 0 '***' 0.001 '**' 0.01 '*' 0.05 '.' 0.1 ' ' 1
##
## (Dispersion parameter for poisson family taken to be 1)
##
## Null deviance: 959.73 on 35 degrees of freedom
## Residual deviance: 712.38 on 28 degrees of freedom
## AIC: 848.62
##
## Number of Fisher Scoring iterations: 6

mod_eggssnail_noRE <- lm( toteggssnailaliveday ~ Treatment * Temperature * Day_c, data = eggssnail ) summary(mod_eggssnail_noRE)

## 4.4. Compare RE vs noRE (LRT + AICc)

anova(mod_eggssnail_pois_RE, mod_eggsnail_pois_noRE)

## Data: eggssnail
## Models:
## mod_eggsnail_pois_noRE: total.eggs ~ Treatment * Temperature * Day_c + offset(log(alive)) + offset(log(days))
## mod_eggssnail_pois_RE: total.eggs ~ Treatment * Temperature * Day_c + offset(log(alive)) + offset(log(days)) + (1 | unit)
## npar AIC BIC logLik -2*log(L) Chisq Df
## mod_eggsnail_pois_noRE 8 848.62 861.29 -416.31 832.62
## mod_eggssnail_pois_RE 9 645.77 660.03 -313.89 627.77 204.84 1
## Pr(>Chisq)
## mod_eggsnail_pois_noRE
## mod_eggssnail_pois_RE < 2.2e-16 ***
## ---
## Signif. codes: 0 '***' 0.001 '**' 0.01 '*' 0.05 '.' 0.1 ' ' 1

MuMIn::AICc(mod_eggsnail_pois_noRE)

## [1] 853.9524

MuMIn::AICc(mod_eggssnail_pois_RE)

## [1] 652.6977

## 4.5. GLMM with random effects model selection

options(na.action = "na.fail")
dredge_eggssnail <- dredge(mod_eggssnail_pois_RE, rank = "AICc")
dredge_eggssnail

## Global model call: glmer(formula = total.eggs ~ Treatment * Temperature * Day_c +
## offset(log(alive)) + offset(log(days)) + (1 | unit), data = eggssnail,
## family = poisson(link = "log"))
## ---
## Model selection table
## (Int) Day_c Tmp Trt Day_c:Tmp Day_c:Trt Tmp:Trt Day_c:Tmp:Trt
## 512 -0.29090 -0.041710 + + + + + +
## 256 0.67740 0.026460 + + + + + +
## 384 2.00500 -0.076060 + + + + + +
## 128 2.97300 -0.007895 + + + + + +
## 416 -0.10420 0.074620 + + + +
## 389 0.08296 +
## 400 -0.09632 0.050120 + + +
## 396 -0.93980 0.050320 + +
## 385 -0.77010
## 391 -0.08273 + +
## 390 0.08320 -0.001930 +
## 432 -0.31040 0.050180 + + + +
## 448 -0.32340 0.074800 + + + + +
## 387 -0.93210 +
## 386 -0.76990 -0.001866
## 406 0.08097 0.011900 + +
## 144 0.87390 0.113200 + + +
## 160 0.86640 0.135500 + + + +
## 140 0.03232 0.113400 + +
## 423 -0.29400 + + +
## 392 -0.08255 -0.001943 + +
## 388 -0.93210 -0.001875 +
## 134 1.05100 0.064420 +
## 408 -0.08782 0.011910 + + +
## 130 0.19980 0.064490
## 176 0.65870 0.113300 + + + +
## 150 1.04900 0.076810 + +
## 192 0.64670 0.135600 + + + + +
## 424 -0.29360 -0.001916 + + +
## 136 0.88690 0.064410 + +
## 132 0.03925 0.064480 +
## 440 -0.29660 0.011920 + + + +
## 152 0.88200 0.076820 + + +
## 168 0.67480 0.064440 + + +
## 184 0.67210 0.076830 + + + +
## 288 2.14700 0.028670 + + + +
## 133 1.04700 +
## 278 2.28700 -0.029410 + +
## 129 0.19320
## 272 2.15200 -0.004525 + + +
## 320 1.97900 0.028800 + + + + +
## 262 2.29000 -0.049820 +
## 268 1.29000 -0.004525 + +
## 135 0.87900 + +
## 258 1.42600 -0.049820
## 131 0.02902 +
## 280 2.15900 -0.029410 + + +
## 32 3.11600 0.089540 + + + +
## 304 1.99400 -0.004525 + + + +
## 264 2.16200 -0.049820 + +
## 5 3.25900 +
## 167 0.67420 + + +
## 22 3.25500 0.035510 + +
## 260 1.30000 -0.049820 +
## 16 3.12100 0.058660 + + +
## 1 2.39500
## 6 3.25800 0.016520 +
## 312 2.00100 -0.029410 + + + +
## 12 2.25900 0.058660 + +
## 64 2.94900 0.089660 + + + + +
## 2 2.39400 0.016520
## 296 2.00400 -0.049820 + + +
## 7 3.13100 + +
## 24 3.12800 0.035510 + + +
## 3 2.26900 +
## 8 3.13000 0.016520 + +
## 48 2.96300 0.058660 + + + +
## 4 2.26800 0.016520 +
## 261 2.27800 +
## 39 2.97300 + + +
## 56 2.97000 0.035510 + + + +
## 257 1.41400
## 40 2.97300 0.016520 + + +
## 263 2.15000 + +
## 259 1.28800 +
## 295 1.99200 + + +
## off(log(alv)) off(log(dys)) df logLik AICc delta weight
## 512 + + 9 -313.887 652.7 0.00 0.983
## 256 + 9 -317.950 660.8 8.13 0.017
## 384 + 9 -330.856 686.6 33.94 0.000
## 128 9 -335.397 695.7 43.02 0.000
## 416 + + 7 -349.634 717.3 64.57 0.000
## 389 + + 3 -355.300 717.4 64.65 0.000
## 400 + + 6 -351.278 717.5 64.76 0.000
## 396 + + 5 -352.911 717.8 65.13 0.000
## 385 + + 2 -356.933 718.2 65.53 0.000
## 391 + + 4 -355.231 719.8 67.05 0.000
## 390 + + 4 -355.292 719.9 67.18 0.000
## 432 + + 7 -351.146 720.3 67.59 0.000
## 448 + + 8 -349.494 720.3 67.62 0.000
## 387 + + 3 -356.882 720.5 67.82 0.000
## 386 + + 3 -356.926 720.6 67.90 0.000
## 406 + + 5 -354.381 720.8 68.06 0.000
## 144 + 6 -353.106 721.1 68.41 0.000
## 160 + 7 -351.653 721.3 68.61 0.000
## 140 + 5 -354.733 721.5 68.77 0.000
## 423 + + 5 -355.102 722.2 69.51 0.000
## 392 + + 5 -355.223 722.4 69.75 0.000
## 388 + + 4 -356.875 723.0 70.34 0.000
## 134 + 4 -356.908 723.1 70.41 0.000
## 408 + + 6 -354.309 723.5 70.82 0.000
## 130 + 3 -358.536 723.8 71.12 0.000
## 176 + 7 -352.972 723.9 71.25 0.000
## 150 + 5 -356.124 724.2 71.55 0.000
## 192 + 8 -351.513 724.4 71.66 0.000
## 424 + + 6 -355.094 725.1 72.39 0.000
## 136 + 5 -356.840 725.7 72.98 0.000
## 132 + 4 -358.486 726.3 73.56 0.000
## 440 + + 7 -354.183 726.4 73.67 0.000
## 152 + 6 -356.054 727.0 74.31 0.000
## 168 + 6 -356.710 728.3 75.62 0.000
## 184 + 7 -355.926 729.9 77.15 0.000
## 288 + 7 -359.690 737.4 84.68 0.000
## 133 + 3 -366.232 739.2 86.52 0.000
## 278 + 5 -363.811 739.6 86.92 0.000
## 129 + 2 -367.877 740.1 87.42 0.000
## 272 + 6 -362.622 740.1 87.44 0.000
## 320 + 8 -359.601 740.5 87.84 0.000
## 262 + 4 -365.756 740.8 88.10 0.000
## 268 + 5 -364.439 740.9 88.18 0.000
## 135 + 4 -366.160 741.6 88.91 0.000
## 258 + 3 -367.560 741.9 89.17 0.000
## 131 + 3 -367.824 742.4 89.70 0.000
## 280 + 6 -363.766 742.4 89.73 0.000
## 32 7 -362.234 742.5 89.77 0.000
## 304 + 7 -362.543 743.1 90.39 0.000
## 264 + 5 -365.711 743.4 90.72 0.000
## 5 3 -368.500 743.8 91.05 0.000
## 167 + 5 -366.038 744.1 91.38 0.000
## 22 5 -366.072 744.1 91.45 0.000
## 260 + 4 -367.528 744.3 91.65 0.000
## 16 6 -364.962 744.8 92.12 0.000
## 1 2 -370.304 745.0 92.27 0.000
## 6 4 -367.881 745.1 92.35 0.000
## 312 + 7 -363.687 745.4 92.68 0.000
## 12 5 -366.779 745.6 92.86 0.000
## 64 8 -362.146 745.6 92.93 0.000
## 2 3 -369.685 746.1 93.42 0.000
## 296 + 6 -365.632 746.2 93.46 0.000
## 7 4 -368.455 746.2 93.50 0.000
## 24 6 -366.026 746.9 94.25 0.000
## 3 3 -370.272 747.3 94.60 0.000
## 8 5 -367.836 747.7 94.97 0.000
## 48 7 -364.883 747.8 95.07 0.000
## 4 4 -369.652 748.6 95.90 0.000
## 261 + 3 -370.973 748.7 96.00 0.000
## 39 5 -368.376 748.8 96.05 0.000
## 56 7 -365.947 749.9 97.20 0.000
## 257 + 2 -372.777 749.9 97.22 0.000
## 40 6 -367.757 750.4 97.71 0.000
## 263 + 4 -370.928 751.1 98.45 0.000
## 259 + 3 -372.745 752.2 99.54 0.000
## 295 + 5 -370.849 753.7 101.00 0.000
## Models ranked by AICc(x)
## Random terms (all models):
## 1 | unit

# averaging (if ΔAICc <= 2)

#avgset_eggssnail <- subset(dredge_eggssnail, delta <= 2) #only one model


#avg_eggssnail <- model.avg(avgset_eggssnail, fit = TRUE)

top_mods_eggssnail <- get.models(dredge_eggssnail, subset = delta <= 2)
top_mods_eggssnail

## $`512`
## Generalized linear mixed model fit by maximum likelihood (Laplace
## Approximation) [glmerMod]
## Family: poisson ( log )
## Formula:
## total.eggs ~ Day_c + Temperature + Treatment + (1 | unit) + Day_c:Temperature +
## Day_c:Treatment + Temperature:Treatment + Day_c:Temperature:Treatment +
## offset(log(alive)) + offset(log(days))
## Data: eggssnail
## AIC BIC logLik -2*log(L) df.resid
## 645.7746 660.0263 -313.8873 627.7746 27
## Random effects:
## Groups Name Std.Dev.
## unit (Intercept) 1.446
## Number of obs: 36, groups: unit, 12
## Fixed Effects:
## (Intercept) Day_c
## -0.29095 -0.04171
## Temperature20 Treatment6PPD-Q
## 0.74085 -1.29621
## Day_c:Temperature20 Day_c:Treatment6PPD-Q
## 0.08447 0.24269
## Temperature20:Treatment6PPD-Q Day_c:Temperature20:Treatment6PPD-Q
## -1.00095 -0.59920
##
## attr(,"rank")
## function (x)
## do.call("rank", list(x))
## <environment: 0x000001e94efa2040>
## attr(,"call")
## AICc(x)
## attr(,"class")
## [1] "function" "rankFunction"
## attr(,"beta")
## [1] "none"

summary(top_mods_eggssnail[[1]])

## Generalized linear mixed model fit by maximum likelihood (Laplace
## Approximation) [glmerMod]
## Family: poisson ( log )
## Formula:
## total.eggs ~ Day_c + Temperature + Treatment + (1 | unit) + Day_c:Temperature +
## Day_c:Treatment + Temperature:Treatment + Day_c:Temperature:Treatment +
## offset(log(alive)) + offset(log(days))
## Data: eggssnail
##
## AIC BIC logLik -2*log(L) df.resid
## 645.8 660.0 -313.9 627.8 27
##
## Scaled residuals:
## Min 1Q Median 3Q Max
## -5.1685 -2.4051 -0.6626 1.1421 7.8061
##
## Random effects:
## Groups Name Variance Std.Dev.
## unit (Intercept) 2.09 1.446
## Number of obs: 36, groups: unit, 12
##
## Fixed effects:
## Estimate Std. Error z value Pr(>|z|)
## (Intercept) -0.29095 0.83827 -0.347 0.7285
## Day_c -0.04171 0.03062 -1.362 0.1732
## Temperature20 0.74085 1.18437 0.626 0.5316
## Treatment6PPD-Q -1.29621 1.22288 -1.060 0.2892
## Day_c:Temperature20 0.08447 0.03851 2.193 0.0283 *
## Day_c:Treatment6PPD-Q 0.24269 0.05096 4.762 1.92e-06 ***
## Temperature20:Treatment6PPD-Q -1.00095 1.71678 -0.583 0.5599
## Day_c:Temperature20:Treatment6PPD-Q -0.59920 0.07314 -8.193 2.55e-16 ***
## ---
## Signif. codes: 0 '***' 0.001 '**' 0.01 '*' 0.05 '.' 0.1 ' ' 1
##
## Correlation of Fixed Effects:
## (Intr) Day_c Tmpr20 T6PPD- Dy_:T20 D_:T6P T20:T6
## Day_c 0.002
## Temperatr20 -0.708 -0.001
## Trtmn6PPD-Q -0.685 -0.001 0.485
## Dy_c:Tmpr20 -0.001 -0.795 -0.003 0.001
## Dy_:T6PPD-Q -0.001 -0.601 0.001 -0.023 0.478
## T20:T6PPD-Q 0.488 0.001 -0.690 -0.701 0.002 0.016
## D_:T20:T6PP 0.001 0.419 0.002 0.016 -0.527 -0.697 0.012

## 4.6. Diagnosis model with random effectes (lmer)

summary(eggssnail)

## replicate Treatment Temperature alive Day total.eggs
## 1:12 CTL :18 15:18 Min. : 4.000 4 :12 Min. : 0.00
## 2:12 6PPD-Q:18 20:18 1st Qu.: 8.000 7 :12 1st Qu.: 0.00
## 3:12 Median :10.000 10:12 Median :13.50
## Mean : 9.139 Mean :21.03
## 3rd Qu.:10.000 3rd Qu.:31.25
## Max. :12.000 Max. :83.00
##
## days toteggssnailaliveday unit Day_c.V1
## Min. :2.000 Min. :0.0000 CTL.15.1 : 3 Min. :-3
## 1st Qu.:2.000 1st Qu.:0.0000 6PPD-Q.15.1: 3 1st Qu.:-3
## Median :3.000 Median :0.5185 CTL.20.1 : 3 Median : 0
## Mean :2.667 Mean :0.8942 6PPD-Q.20.1: 3 Mean : 0
## 3rd Qu.:3.000 3rd Qu.:1.4139 CTL.15.2 : 3 3rd Qu.: 3
## Max. :3.000 Max. :3.5714 6PPD-Q.15.2: 3 Max. : 3
## (Other) :18

mod_avg_eggssnail_RE <- glmer(total.eggs ~ Treatment * Temperature * Day_c +
 offset(log(alive)) + offset(log(days)) +
 (1 | unit),
 data = eggssnail,
 family = poisson(link = "log")
)
check_model(mod_avg_eggssnail_RE )

MuMIn::AICc(mod_avg_eggssnail_RE)

## [1] 652.6977

performance::r2(mod_avg_eggssnail_RE) # Marginal and conditional R2

## # R2 for Mixed Models
##
## Conditional R2: 0.983
## Marginal R2: 0.345

insight::get_variance_random(mod_avg_eggssnail_RE)

## var.random
## 2.089775

insight::get_variance_fixed(mod_avg_eggssnail_RE)

## var.fixed
## 1.129048

performance::r2_nakagawa(mod_avg_eggssnail_RE)

## # R2 for Mixed Models
##
## Conditional R2: 0.983
## Marginal R2: 0.345

nlevels(eggssnail$unit); table(eggssnail$unit)

## [1] 12

##
## CTL.15.1 6PPD-Q.15.1 CTL.20.1 6PPD-Q.20.1 CTL.15.2 6PPD-Q.15.2
## 3 3 3 3 3 3
## CTL.20.2 6PPD-Q.20.2 CTL.15.3 6PPD-Q.15.3 CTL.20.3 6PPD-Q.20.3
## 3 3 3 3 3 3

lme4::VarCorr(mod_avg_eggssnail_RE)

## Groups Name Std.Dev.
## unit (Intercept) 1.4456

lme4::isSingular(mod_avg_eggssnail_RE, tol = 1e-5)

## [1] FALSE

mod_avg_eggssnail_LM <- lm(toteggssnailaliveday ~ Treatment * Temperature, data = eggssnail)
summary(mod_avg_eggssnail_LM) #estimates and significance very similar to the LMER

##
## Call:
## lm(formula = toteggssnailaliveday ~ Treatment * Temperature,
## data = eggssnail)
##
## Residuals:
## Min 1Q Median 3Q Max
## -1.6334 -0.5866 -0.2891 0.5909 2.1574
##
## Coefficients:
## Estimate Std. Error t value Pr(>|t|)
## (Intercept) 0.7929 0.3180 2.493 0.0180 *
## Treatment6PPD-Q -0.2003 0.4498 -0.445 0.6591
## Temperature20 0.8405 0.4498 1.869 0.0708 .
## Treatment6PPD-Q:Temperature20 -0.8754 0.6361 -1.376 0.1783
## ---
## Signif. codes: 0 '***' 0.001 '**' 0.01 '*' 0.05 '.' 0.1 ' ' 1
##
## Residual standard error: 0.9541 on 32 degrees of freedom
## Multiple R-squared: 0.1903, Adjusted R-squared: 0.1144
## F-statistic: 2.508 on 3 and 32 DF, p-value: 0.07653

MuMIn::AICc(mod_avg_eggssnail_RE)

## [1] 652.6977

MuMIn::AICc(mod_avg_eggssnail_LM)

## [1] 106.5395

performance::r2(mod_avg_eggssnail_LM)

## # R2 for Linear Regression
## R2: 0.190
## adj. R2: 0.114

res <- simulateResiduals(mod_avg_eggssnail_RE )
plot(res)
testDispersion(res)

##
## DHARMa nonparametric dispersion test via sd of residuals fitted vs.
## simulated
##
## data: simulationOutput
## dispersion = 0.022647, p-value = 0.328
## alternative hypothesis: two.sided

testZeroInflation(res) #there is inflation

##
## DHARMa zero-inflation test via comparison to expected zeros with
## simulation under H0 = fitted model
##
## data: simulationOutput
## ratioObsSim = 3.9012, p-value < 2.2e-16
## alternative hypothesis: two.sided

testUniformity(res)

##
## Exact one-sample Kolmogorov-Smirnov test
##
## data: simulationOutput$scaledResiduals
## D = 0.20943, p-value = 0.07299
## alternative hypothesis: two-sided

# Zero inflation Poisson
mod_eggsnail_ziPoisson_RE <- glmmTMB(
 total.eggs ~ Treatment * Temperature * Day_c +
 offset(log(alive)) + offset(log(days)) +
 (1 | unit),
 ziformula = ~ 1,
 family = poisson(), data = eggssnail
)

summary(mod_eggsnail_ziPoisson_RE)

## Family: poisson ( log )
## Formula:
## total.eggs ~ Treatment * Temperature * Day_c + offset(log(alive)) +
## offset(log(days)) + (1 | unit)
## Zero inflation: ~1
## Data: eggssnail
##
## AIC BIC logLik -2*log(L) df.resid
## 426.7 442.6 -203.4 406.7 26
##
## Random effects:
##
## Conditional model:
## Groups Name Variance Std.Dev.
## unit (Intercept) 0.07589 0.2755
## Number of obs: 36, groups: unit, 12
##
## Conditional model:
## Estimate Std. Error z value Pr(>|z|)
## (Intercept) -0.25999 0.17632 -1.475 0.140340
## Treatment6PPD-Q 0.39806 0.28092 1.417 0.156486
## Temperature20 0.98256 0.24475 4.015 5.95e-05 ***
## Day_c -0.04188 0.03074 -1.362 0.173086
## Treatment6PPD-Q:Temperature20 -1.05529 0.40518 -2.604 0.009201 **
## Treatment6PPD-Q:Day_c 0.25295 0.06511 3.885 0.000102 ***
## Temperature20:Day_c 0.02191 0.03886 0.564 0.572909
## Treatment6PPD-Q:Temperature20:Day_c -0.30580 0.09447 -3.237 0.001208 **
## ---
## Signif. codes: 0 '***' 0.001 '**' 0.01 '*' 0.05 '.' 0.1 ' ' 1
##
## Zero-inflation model:
## Estimate Std. Error z value Pr(>|z|)
## (Intercept) -0.6932 0.3536 -1.96 0.0499 *
## ---
## Signif. codes: 0 '***' 0.001 '**' 0.01 '*' 0.05 '.' 0.1 ' ' 1

performance::r2(mod_eggsnail_ziPoisson_RE)

## # R2 for Mixed Models
##
## Conditional R2: 0.083
## Marginal R2: 0.061

# Diagnòstics DHARMa
res_zip <- simulateResiduals(mod_eggsnail_ziPoisson_RE)
plot(res_zip)


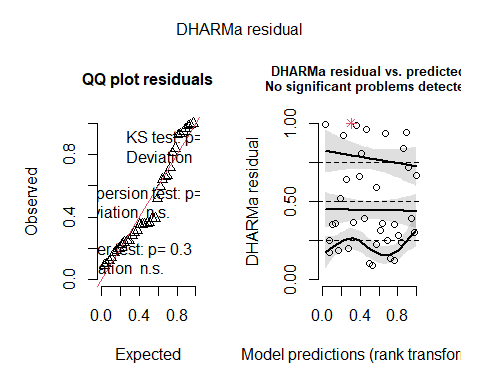


testDispersion(res_zip)


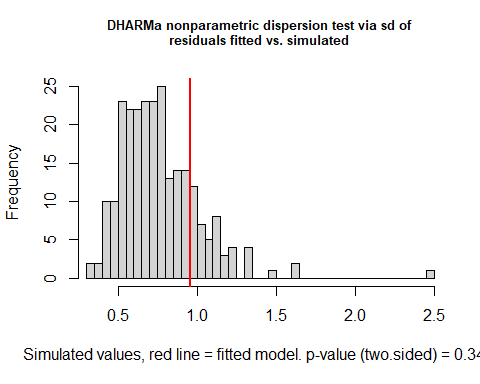


##
## DHARMa nonparametric dispersion test via sd of residuals fitted vs.
## simulated
##
## data: simulationOutput
## dispersion = 1.2569, p-value = 0.344
## alternative hypothesis: two.sided

testZeroInflation(res_zip)


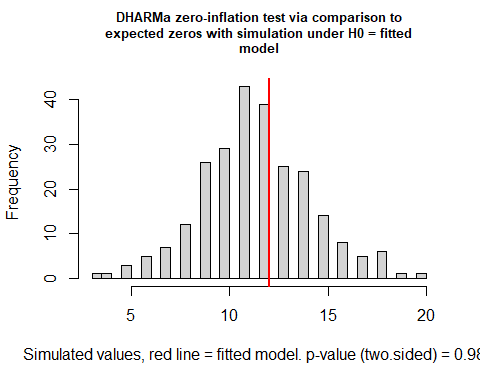


##
## DHARMa zero-inflation test via comparison to expected zeros with
## simulation under H0 = fitted model
##
## data: simulationOutput
## ratioObsSim = 1.037, p-value = 0.984
## alternative hypothesis: two.sided

testUniformity(res_zip)


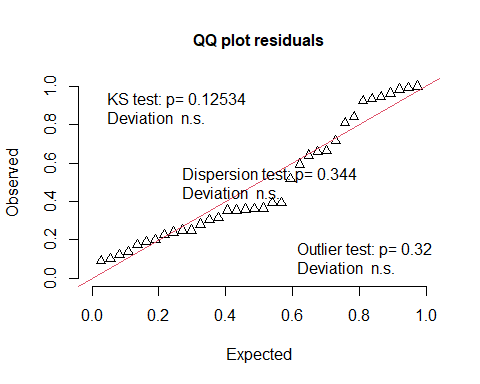


##
## Exact one-sample Kolmogorov-Smirnov test
##
## data: simulationOutput$scaledResiduals
## D = 0.19133, p-value = 0.1253
## alternative hypothesis: two-sided

## 4.7. GLMM zero inflation poisson with random effects model selection

options(na.action = "na.fail")
dredge_eggssnail_ziP <- dredge(mod_eggsnail_ziPoisson_RE, rank = "AICc")
dredge_eggssnail_ziP

## Global model call: glmmTMB(formula = total.eggs ~ Treatment * Temperature * Day_c +
## offset(log(alive)) + offset(log(days)) + (1 | unit), data = eggssnail,
## family = poisson(), ziformula = ~1, dispformula = ~1)
## ---
## Model selection table
## cnd((Int)) zi((Int)) dsp((Int)) cnd(Day_c) cnd(Tmp) cnd(Trt) cnd(Day_c:Tmp)
## 184 0.70950 -0.6931 + 0.034510 + +
## 152 0.87090 -0.6931 + 0.035400 + +
## 256 0.71030 -0.6931 + 0.026280 + + +
## 192 0.70930 -0.6931 + 0.046920 + + +
## 150 1.19500 -0.6931 + 0.036080 +
## 56 3.00600 -0.6931 + -0.009712 + +
## 160 0.87260 -0.6931 + 0.044900 + + +
## 24 3.15000 -0.6931 + -0.009094 + +
## 132 0.89430 -0.6932 + 0.057750 +
## 3 3.15100 -0.6931 + +
## 1 3.37400 -0.6931 +
## 130 1.18100 -0.6932 + 0.056240
## 168 0.70350 -0.6931 + 0.056360 + +
## 39 3.00000 -0.6931 + + +
## 22 3.41200 -0.6931 + -0.008727 +
## 64 3.00700 -0.6931 + 0.009010 + + +
## 140 0.89230 -0.6931 + 0.073820 + +
## 32 3.15300 -0.6931 + 0.006409 + + +
## 4 3.15100 -0.6931 + 0.011940 +
## 128 3.00900 -0.6931 + -0.007896 + + +
## 136 0.90040 -0.6932 + 0.057690 + +
## 2 3.37600 -0.6931 + 0.010230
## 7 3.18400 -0.6931 + + +
## 5 3.40900 -0.6931 + +
## 512 -0.26000 -0.6932 + -0.041880 + + +
## 176 0.70060 -0.6931 + 0.076430 + + +
## 134 1.19000 -0.6932 + 0.056180 +
## 40 3.00000 -0.6931 + 0.010680 + +
## 12 3.14800 -0.6931 + 0.033150 + +
## 144 0.90210 -0.6931 + 0.073800 + + +
## 8 3.17900 -0.6931 + 0.011650 + +
## 6 3.40600 -0.6931 + 0.009990 +
## 48 2.99800 -0.6931 + 0.036490 + + +
## 387 -0.08903 -0.6932 + +
## 440 -0.26130 -0.6931 + -0.028070 + +
## 423 -0.26750 -0.6931 + + +
## 384 2.04300 -0.6931 + -0.076060 + + +
## 385 0.20340 -0.6931 +
## 16 3.18300 -0.6931 + 0.033170 + + +
## 408 -0.08789 -0.6931 + -0.027150 + +
## 388 -0.08837 -0.6932 + -0.007638 +
## 391 -0.06969 -0.6932 + + +
## 131 0.89030 -0.6932 + +
## 312 2.03800 -0.6931 + -0.073210 + +
## 167 0.70000 -0.6932 + + +
## 386 0.20270 -0.6932 + -0.009004
## 129 1.16700 -0.6932 +
## 424 -0.26620 -0.6932 + -0.009164 + +
## 406 0.23170 -0.6931 + -0.026480 +
## 389 0.22450 -0.6932 + +
## 448 -0.26230 -0.6932 + -0.009542 + + +
## 280 2.19200 -0.6931 + -0.072490 + +
## 396 -0.09122 -0.6932 + 0.013330 + +
## 260 2.16900 -0.6931 + -0.054020 +
## 416 -0.08591 -0.6931 + -0.011770 + + +
## 258 2.39800 -0.6932 + -0.055690
## 392 -0.06604 -0.6932 + -0.007836 + +
## 320 2.03800 -0.6931 + -0.047660 + + +
## 296 2.03300 -0.6931 + -0.055390 + +
## 135 0.91790 -0.6932 + + +
## 278 2.44900 -0.6931 + -0.072050 +
## 268 2.16600 -0.6931 + -0.027690 + +
## 432 -0.27010 -0.6931 + 0.015700 + + +
## 133 1.19700 -0.6932 + +
## 390 0.22720 -0.6932 + -0.009153 +
## 288 2.19500 -0.6932 + -0.050750 + + +
## 304 2.03000 -0.6931 + -0.024130 + + +
## 264 2.21400 -0.6931 + -0.054430 + +
## 262 2.44400 -0.6931 + -0.056020 +
## 400 -0.06468 -0.6932 + 0.013280 + + +
## 272 2.21700 -0.6931 + -0.027630 + + +
## 259 2.16100 -0.6931 + +
## 257 2.39600 -0.6931 +
## 295 2.02300 -0.6931 + + +
## 263 2.18400 -0.6931 + + +
## 261 2.42000 -0.6931 + +
## cnd(Day_c:Trt) cnd(Tmp:Trt) cnd(Day_c:Tmp:Trt) cnd(off(log(alv)))
## 184 + + +
## 152 + +
## 256 + + + +
## 192 + + +
## 150 + +
## 56 + +
## 160 + +
## 24 +
## 132 +
## 3
## 1
## 130 +
## 168 + +
## 39 +
## 22 +
## 64 + +
## 140 +
## 32 +
## 4
## 128 + + +
## 136 +
## 2
## 7
## 5
## 512 + + + +
## 176 + +
## 134 +
## 40 +
## 12
## 144 +
## 8
## 6
## 48 +
## 387 +
## 440 + + +
## 423 + +
## 384 + + +
## 385 +
## 16
## 408 + +
## 388 +
## 391 +
## 131 +
## 312 + +
## 167 + +
## 386 +
## 129 +
## 424 + +
## 406 + +
## 389 +
## 448 + + +
## 280 +
## 396 +
## 260
## 416 + +
## 258
## 392 +
## 320 + +
## 296 +
## 135 +
## 278 +
## 268
## 432 + +
## 133 +
## 390 +
## 288 +
## 304 +
## 264
## 262
## 400 +
## 272
## 259
## 257
## 295 +
## 263
## 261
## cnd(off(log(dys))) df logLik AICc delta weight
## 184 8 -203.290 427.9 0.00 0.261
## 152 7 -205.273 428.5 0.63 0.190
## 256 10 -200.724 430.2 2.33 0.081
## 192 9 -203.121 431.2 3.25 0.051
## 150 6 -208.150 431.2 3.28 0.051
## 56 8 -205.080 431.5 3.58 0.044
## 160 8 -205.171 431.7 3.76 0.040
## 24 7 -207.047 432.1 4.18 0.032
## 132 5 -210.048 432.1 4.18 0.032
## 3 4 -211.708 432.7 4.79 0.024
## 1 3 -213.133 433.0 5.10 0.020
## 130 4 -212.030 433.3 5.44 0.017
## 168 7 -207.733 433.5 5.55 0.016
## 39 6 -209.375 433.6 5.73 0.015
## 22 6 -209.485 433.9 5.95 0.013
## 64 9 -204.698 434.3 6.41 0.011
## 140 6 -209.730 434.4 6.44 0.010
## 32 8 -206.776 434.9 6.97 0.008
## 4 5 -211.456 434.9 7.00 0.008
## 128 10 -203.091 435.0 7.07 0.008
## 136 6 -210.046 435.0 7.08 0.008
## 2 4 -212.947 435.2 7.27 0.007
## 7 5 -211.655 435.3 7.40 0.006
## 5 4 -213.089 435.5 7.55 0.006
## 512 + 10 -203.358 435.5 7.60 0.006
## 176 8 -207.242 435.8 7.90 0.005
## 134 5 -212.027 436.1 8.14 0.004
## 40 7 -209.172 436.3 8.43 0.004
## 12 6 -210.901 436.7 8.79 0.003
## 144 7 -209.725 437.5 9.54 0.002
## 8 6 -211.415 437.7 9.81 0.002
## 6 5 -212.912 437.8 9.91 0.002
## 48 8 -208.365 438.1 10.15 0.002
## 387 + 4 -214.429 438.1 10.23 0.002
## 440 + 8 -208.951 439.2 11.32 0.001
## 423 + 6 -212.177 439.3 11.34 0.001
## 384 + 10 -205.317 439.4 11.52 0.001
## 385 + 3 -216.427 439.6 11.69 0.001
## 16 7 -210.842 439.7 11.77 0.001
## 408 + 7 -211.000 440.0 12.09 0.001
## 388 + 5 -214.330 440.7 12.75 0.000
## 391 + 5 -214.413 440.8 12.91 0.000
## 131 4 -215.940 441.2 13.26 0.000
## 312 + 8 -210.007 441.3 13.43 0.000
## 167 6 -213.365 441.6 13.71 0.000
## 386 + 4 -216.291 441.9 13.96 0.000
## 129 3 -217.620 442.0 14.08 0.000
## 424 + 7 -212.035 442.1 14.16 0.000
## 406 + 6 -213.606 442.1 14.19 0.000
## 389 + 4 -216.415 442.1 14.21 0.000
## 448 + 9 -208.602 442.1 14.21 0.000
## 280 + 7 -212.195 442.4 14.48 0.000
## 396 + 6 -213.828 442.6 14.64 0.000
## 260 + 5 -215.331 442.7 14.75 0.000
## 416 + 8 -210.753 442.8 14.93 0.000
## 258 + 4 -216.867 443.0 15.11 0.000
## 392 + 6 -214.309 443.5 15.60 0.000
## 320 + 9 -209.350 443.6 15.71 0.000
## 296 + 7 -212.880 443.8 15.85 0.000
## 135 5 -215.910 443.8 15.91 0.000
## 278 + 6 -214.463 443.8 15.91 0.000
## 268 + 6 -214.536 444.0 16.06 0.000
## 432 + 8 -211.333 444.0 16.09 0.000
## 133 4 -217.596 444.5 16.57 0.000
## 390 + 5 -216.273 444.5 16.63 0.000
## 288 + 8 -211.701 444.7 16.82 0.000
## 304 + 8 -211.778 444.9 16.98 0.000
## 264 + 6 -215.230 445.4 17.44 0.000
## 262 + 5 -216.785 445.6 17.66 0.000
## 400 + 7 -213.798 445.6 17.68 0.000
## 272 + 7 -214.403 446.8 18.89 0.000
## 259 + 4 -220.247 449.8 21.87 0.000
## 257 + 3 -222.112 451.0 23.06 0.000
## 295 + 6 -218.086 451.1 23.16 0.000
## 263 + 5 -220.217 452.4 24.52 0.000
## 261 + 4 -222.087 453.5 25.55 0.000
## Models ranked by AICc(x)
## Random terms (all models):
## cond(1 | unit)

# averaging (if ΔAICc <= 2)

avgset_eggssnail_ziP <- subset(dredge_eggssnail_ziP, delta <= 2) #only one model


#avg_eggssnail_ziP <- model.avg(avgset_eggssnail_ziP, fit = TRUE)
#summary(avg_eggssnail_ziP)

top_mods_eggssnail_ziP <- get.models(avgset_eggssnail_ziP, subset = delta <= 2)
summary(top_mods_eggssnail_ziP[[1]])

## Family: poisson ( log )
## Formula:
## total.eggs ~ Day_c + Temperature + Treatment + (1 | unit) + Day_c:Treatment +
## Temperature:Treatment + offset(log(alive))
## Zero inflation: ~1
## Data: eggssnail
##
## AIC BIC logLik -2*log(L) df.resid
## 422.6 435.2 -203.3 406.6 28
##
## Random effects:
##
## Conditional model:
## Groups Name Variance Std.Dev.
## unit (Intercept) 0.07271 0.2696
## Number of obs: 36, groups: unit, 12
##
## Conditional model:
## Estimate Std. Error z value Pr(>|z|)
## (Intercept) 0.70945 0.17326 4.095 4.23e-05 ***
## Day_c 0.03451 0.01831 1.885 0.0594 .
## Temperature20 0.97386 0.24024 4.054 5.04e-05 ***
## Treatment6PPD-Q 0.47553 0.27435 1.733 0.0830 .
## Day_c:Treatment6PPD-Q 0.13743 0.04615 2.978 0.0029 **
## Temperature20:Treatment6PPD-Q -0.84945 0.39151 -2.170 0.0300 *
## ---
## Signif. codes: 0 '***' 0.001 '**' 0.01 '*' 0.05 '.' 0.1 ' ' 1
##
## Zero-inflation model:
## Estimate Std. Error z value Pr(>|z|)
## (Intercept) -0.6931 0.3536 -1.96 0.0499 *
## ---
## Signif. codes: 0 '***' 0.001 '**' 0.01 '*' 0.05 '.' 0.1 ' ' 1

#final model zero inflation Poisson
mod_eggsnail_ziPoisson_RE <- glmmTMB(
 total.eggs ~ Treatment * Temperature * Day_c +
 offset(log(alive)) + offset(log(days)) +
 (1 | unit),
 ziformula = ~ 1, # component de zeros constant
 family = poisson(), data = eggssnail
)
summary(mod_eggsnail_ziPoisson_RE)

## Family: poisson ( log )
## Formula:
## total.eggs ~ Treatment * Temperature * Day_c + offset(log(alive)) +
## offset(log(days)) + (1 | unit)
## Zero inflation: ~1
## Data: eggssnail
##
## AIC BIC logLik -2*log(L) df.resid
## 426.7 442.6 -203.4 406.7 26
##
## Random effects:
##
## Conditional model:
## Groups Name Variance Std.Dev.
## unit (Intercept) 0.07589 0.2755
## Number of obs: 36, groups: unit, 12
##
## Conditional model:
## Estimate Std. Error z value Pr(>|z|)
## (Intercept) -0.25999 0.17632 -1.475 0.140340
## Treatment6PPD-Q 0.39806 0.28092 1.417 0.156486
## Temperature20 0.98256 0.24475 4.015 5.95e-05 ***
## Day_c -0.04188 0.03074 -1.362 0.173086
## Treatment6PPD-Q:Temperature20 -1.05529 0.40518 -2.604 0.009201 **
## Treatment6PPD-Q:Day_c 0.25295 0.06511 3.885 0.000102 ***
## Temperature20:Day_c 0.02191 0.03886 0.564 0.572909
## Treatment6PPD-Q:Temperature20:Day_c -0.30580 0.09447 -3.237 0.001208 **
## ---
## Signif. codes: 0 '***' 0.001 '**' 0.01 '*' 0.05 '.' 0.1 ' ' 1
##
## Zero-inflation model:
## Estimate Std. Error z value Pr(>|z|)
## (Intercept) -0.6932 0.3536 -1.96 0.0499 *
## ---
## Signif. codes: 0 '***' 0.001 '**' 0.01 '*' 0.05 '.' 0.1 ' ' 1

# Diagnòstics DHARMa
res_P_zi <- simulateResiduals(mod_eggsnail_ziPoisson_RE)
plot(res_P_zi)


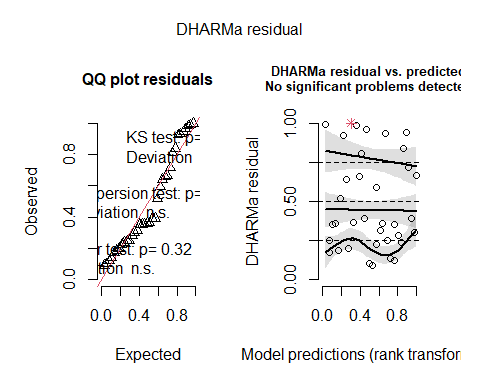


testDispersion(res_P_zi)


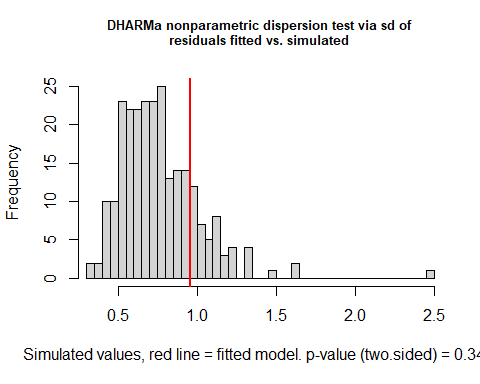


##
## DHARMa nonparametric dispersion test via sd of residuals fitted vs.
## simulated
##
## data: simulationOutput
## dispersion = 1.2569, p-value = 0.344
## alternative hypothesis: two.sided

testZeroInflation(res_P_zi) # hauria de millorar vs Poisson/LMM


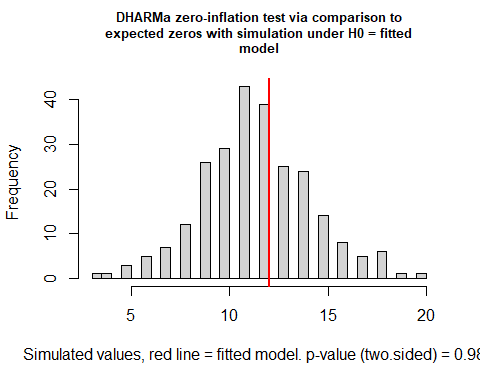


##
## DHARMa zero-inflation test via comparison to expected zeros with
## simulation under H0 = fitted model
##
## data: simulationOutput
## ratioObsSim = 1.037, p-value = 0.984
## alternative hypothesis: two.sided

testUniformity(res_P_zi)


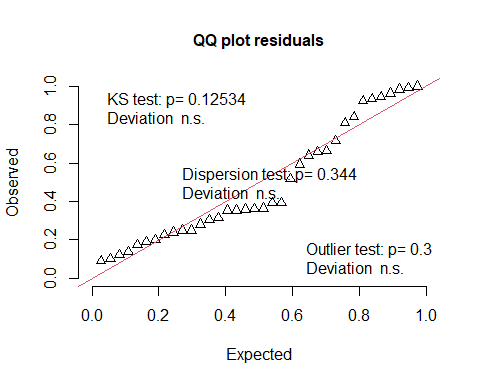


##
## Exact one-sample Kolmogorov-Smirnov test
##
## data: simulationOutput$scaledResiduals
## D = 0.19133, p-value = 0.1253
## alternative hypothesis: two-sided

# Zero-inflated Poisson (ZIP) non random effects
mod_eggsnail_ziPoisson_noRE <- glmmTMB(
 total.eggs ~ Treatment * Temperature * Day_c +
 offset(log(alive)) + offset(log(days)),
 ziformula = ~ 1, # component de zeros constant
 family = poisson(),
 data = eggssnail
)

summary(mod_eggsnail_ziPoisson_noRE)

## Family: poisson ( log )
## Formula:
## total.eggs ~ Treatment * Temperature * Day_c + offset(log(alive)) +
## offset(log(days))
## Zero inflation: ~1
## Data: eggssnail
##
## AIC BIC logLik -2*log(L) df.resid
## 450.2 464.5 -216.1 432.2 27
##
##
## Conditional model:
## Estimate Std. Error z value Pr(>|z|)
## (Intercept) -0.217995 0.072562 -3.004 0.002662 **
## Treatment6PPD-Q 0.363285 0.122674 2.961 0.003063 **
## Temperature20 0.902878 0.091373 9.881 < 2e-16 ***
## Day_c -0.042636 0.030740 -1.387 0.165455
## Treatment6PPD-Q:Temperature20 -0.954642 0.189710 -5.032 4.85e-07 ***
## Treatment6PPD-Q:Day_c 0.253712 0.055530 4.569 4.90e-06 ***
## Temperature20:Day_c 0.008436 0.038658 0.218 0.827256
## Treatment6PPD-Q:Temperature20:Day_c -0.292889 0.088056 -3.326 0.000881 ***
## ---
## Signif. codes: 0 '***' 0.001 '**' 0.01 '*' 0.05 '.' 0.1 ' ' 1
##
## Zero-inflation model:
## Estimate Std. Error z value Pr(>|z|)
## (Intercept) -0.6931 0.3536 -1.96 0.0499 *
## ---
## Signif. codes: 0 '***' 0.001 '**' 0.01 '*' 0.05 '.' 0.1 ' ' 1

# Comparison
MuMIn::AICc(mod_eggsnail_ziPoisson_RE )

## [1] 435.517

MuMIn::AICc(mod_eggsnail_ziPoisson_noRE)

## [1] 457.1665

# 5. ANALYSIS OF DEVELOPMENT

## 5.1. Load data

development <- read.csv("development.csv", sep = ";", header = TRUE)


summary (development)

## replicate Treatment Temperature clutch
## Min. :1.000 Length:54 Min. :15.00 Min. : 2.10
## 1st Qu.:1.000 Class :character 1st Qu.:15.00 1st Qu.: 4.20
## Median :2.000 Mode :character Median :20.00 Median : 7.10
## Mean :1.889 Mean :17.69 Mean : 6.82
## 3rd Qu.:2.750 3rd Qu.:20.00 3rd Qu.: 9.45
## Max. :3.000 Max. :20.00 Max. :10.40
## Day eggs nonhatched nondeveloped
## Min. : 2.00 Min. : 2.00 Min. : 0.000 Min. :0.000
## 1st Qu.: 4.00 1st Qu.: 9.00 1st Qu.: 0.250 1st Qu.:0.000
## Median : 7.00 Median :13.00 Median : 2.000 Median :0.000
## Mean : 6.63 Mean :13.43 Mean : 5.296 Mean :1.259
## 3rd Qu.: 9.25 3rd Qu.:17.00 3rd Qu.: 8.750 3rd Qu.:1.000
## Max. :10.00 Max. :33.00 Max. :23.000 Max. :9.000

development$replicate <- factor(development$replicate)
development$Treatment <- factor(development$Treatment, levels = c("CTL","6PPD-Q"))
development$Temperature <- factor(development$Temperature)
development$clutch <- factor(development$clutch)
development$Day_c <- scale(development$Day, center = TRUE, scale = FALSE)
development$unit <- interaction(development$Treatment, development$Temperature, development$replicate)
summary(development)

## replicate Treatment Temperature clutch Day eggs
## 1:20 CTL :35 15:25 7.1 : 9 Min. : 2.00 Min. : 2.00
## 2:20 6PPD-Q:19 20:29 4.1 : 8 1st Qu.: 4.00 1st Qu.: 9.00
## 3:14 10.1 : 7 Median : 7.00 Median :13.00
## 4.2 : 6 Mean : 6.63 Mean :13.43
## 7.2 : 6 3rd Qu.: 9.25 3rd Qu.:17.00
## 4.3 : 4 Max. :10.00 Max. :33.00
## (Other):14
## nonhatched nondeveloped Day_c.V1 unit
## Min. : 0.000 Min. :0.000 Min. :-4.62962962963 CTL.20.2 :10
## 1st Qu.: 0.250 1st Qu.:0.000 1st Qu.:-2.62962962963 CTL.15.2 : 7
## Median : 2.000 Median :0.000 Median : 0.37037037037 6PPD-Q.20.1: 6
## Mean : 5.296 Mean :1.259 Mean : 0.00000000000 CTL.20.3 : 6
## 3rd Qu.: 8.750 3rd Qu.:1.000 3rd Qu.: 2.62037037037 CTL.15.1 : 5
## Max. :23.000 Max. :9.000 Max. : 3.37037037037 6PPD-Q.15.1: 5
## (Other) :15

hist(development$nonhatched,
 col = "grey",
 main = "Non hatched distribution",
 xlab = "nonhatched")


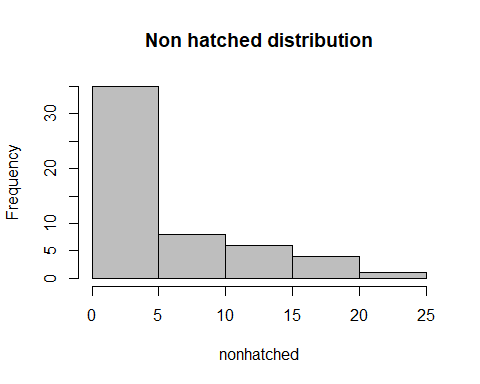


# 5.2. GLMM Binomial for non-hatched

##

mod_nonhatched_RE <- glmmTMB(
 cbind(nonhatched, eggs - nonhatched) ~
 Treatment * Temperature * Day_c +
 (1 | unit),
 family = binomial(link = "logit"),
 data = development
)

summary(mod_nonhatched_RE)

## Family: binomial ( logit )
## Formula:
## cbind(nonhatched, eggs - nonhatched) ~ Treatment * Temperature *
## Day_c + (1 | unit)
## Data: development
##
## AIC BIC logLik -2*log(L) df.resid
## 289.6 307.5 -135.8 271.6 45
##
## Random effects:
##
## Conditional model:
## Groups Name Variance Std.Dev.
## unit (Intercept) 0.5973 0.7729
## Number of obs: 54, groups: unit, 10
##
## Conditional model:
## Estimate Std. Error z value Pr(>|z|)
## (Intercept) -2.2723 0.5252 -4.326 1.52e-05 ***
## Treatment6PPD-Q 0.3532 0.8229 0.429 0.667765
## Temperature20 2.6418 0.7043 3.751 0.000176 ***
## Day_c -0.2691 0.1344 -2.003 0.045194 *
## Treatment6PPD-Q:Temperature20 0.0358 1.1312 0.032 0.974753
## Treatment6PPD-Q:Day_c -0.2696 0.2596 -1.038 0.299127
## Temperature20:Day_c 0.5716 0.1479 3.865 0.000111 ***
## Treatment6PPD-Q:Temperature20:Day_c 0.5097 0.2967 1.718 0.085813 .
## ---
## Signif. codes: 0 '***' 0.001 '**' 0.01 '*' 0.05 '.' 0.1 ' ' 1

# 5.3. GLM no random effects for non-hatched

##

mod_nonhatched_noRE <- glmmTMB(
 cbind(nonhatched, eggs - nonhatched) ~
 Treatment * Temperature * Day_c ,
 family = binomial(link = "logit"),
 data = development
)

summary(mod_nonhatched_noRE)

## Family: binomial ( logit )
## Formula:
## cbind(nonhatched, eggs - nonhatched) ~ Treatment * Temperature * Day_c
## Data: development
##
## AIC BIC logLik -2*log(L) df.resid
## 307.9 323.8 -145.9 291.9 46
##
##
## Conditional model:
## Estimate Std. Error z value Pr(>|z|)
## (Intercept) -2.21436 0.25281 -8.759 < 2e-16 ***
## Treatment6PPD-Q 0.45629 0.37417 1.219 0.222669
## Temperature20 2.85558 0.28398 10.055 < 2e-16 ***
## Day_c -0.27694 0.13217 -2.095 0.036137 *
## Treatment6PPD-Q:Temperature20 -0.47757 0.47081 -1.014 0.310414
## Treatment6PPD-Q:Day_c 0.15878 0.16939 0.937 0.348564
## Temperature20:Day_c 0.52571 0.14204 3.701 0.000215 ***
## Treatment6PPD-Q:Temperature20:Day_c 0.09576 0.21704 0.441 0.659079
## ---
## Signif. codes: 0 '***' 0.001 '**' 0.01 '*' 0.05 '.' 0.1 ' ' 1

# 5.3. GLMM with random effects model selection for non-hatched

library(MuMIn)

options(na.action = "na.fail")
dd <- dredge(mod_nonhatched_RE, rank = "AICc")
dd

## Global model call: glmmTMB(formula = cbind(nonhatched, eggs - nonhatched) ~ Treatment *
## Temperature * Day_c + (1 | unit), data = development, family = binomial(link = "logit"),
## ziformula = ~0, dispformula = ~1)
## ---
## Model selection table
## cnd((Int)) dsp((Int)) cnd(Day_c) cnd(Tmp) cnd(Trt) cnd(Day_c:Tmp)
## 12 -2.1390 + -0.3505 + +
## 16 -2.2410 + -0.3524 + + +
## 32 -2.2790 + -0.3913 + + +
## 48 -2.3040 + -0.3532 + + +
## 128 -2.2720 + -0.2691 + + +
## 64 -2.3260 + -0.3922 + + +
## 4 -2.3340 + 0.1909 +
## 8 -2.2980 + 0.1898 + +
## 40 -2.3570 + 0.1867 + +
## 24 -2.2980 + 0.1905 + +
## 56 -2.3610 + 0.1911 + +
## 3 -2.1680 + +
## 7 -2.0740 + + +
## 39 -2.2390 + + +
## 2 -0.9907 + 0.1849
## 6 -0.9705 + 0.1848 +
## 22 -0.9830 + 0.2029 +
## 1 -0.9303 +
## 5 -0.8382 + +
## cnd(Day_c:Trt) cnd(Tmp:Trt) cnd(Day_c:Tmp:Trt) df logLik AICc delta
## 12 5 -138.033 287.3 0.00
## 16 6 -137.893 289.6 2.26
## 32 + 7 -137.426 291.3 3.97
## 48 + 7 -137.849 292.1 4.82
## 128 + + + 9 -135.824 293.7 6.42
## 64 + + 8 -137.400 294.0 6.68
## 4 4 -155.414 319.6 32.33
## 8 5 -155.382 322.0 34.70
## 40 + 6 -155.302 324.4 37.07
## 24 + 6 -155.381 324.6 37.23
## 56 + + 7 -155.286 327.0 39.69
## 3 3 -165.333 337.1 49.83
## 7 4 -165.134 339.1 51.77
## 39 + 5 -164.575 340.4 53.08
## 2 3 -167.033 340.5 53.23
## 6 4 -167.031 342.9 55.56
## 22 + 5 -166.777 344.8 57.49
## 1 2 -175.166 354.6 67.25
## 5 3 -175.133 356.7 69.43
## weight
## 12 0.615
## 16 0.199
## 32 0.084
## 48 0.055
## 128 0.025
## 64 0.022
## 4 0.000
## 8 0.000
## 40 0.000
## 24 0.000
## 56 0.000
## 3 0.000
## 7 0.000
## 39 0.000
## 2 0.000
## 6 0.000
## 22 0.000
## 1 0.000
## 5 0.000
## Models ranked by AICc(x)
## Random terms (all models):
## cond(1 | unit)

top_mods_nonhatch <- get.models(dd, subset = delta <= 2)
top_mods_nonhatch

## $`12`
## Formula: cbind(nonhatched, eggs - nonhatched) ~ Day_c + Temperature +
## (1 | unit) + Day_c:Temperature
## Data: development
## AIC BIC logLik -2*log(L) df.resid
## 286.0665 296.0114 -138.0332 276.0665 49
## Random-effects (co)variances:
##
## Conditional model:
## Groups Name Std.Dev.
## unit (Intercept) 0.6489
##
## Number of obs: 54 / Conditional model: unit, 10
##
## Fixed Effects:
##
## Conditional model:
## (Intercept) Day_c Temperature20
## -2.1389 -0.3505 2.5548
## Day_c:Temperature20
## 0.6945
##
## attr(,"rank")
## function (x)
## do.call("rank", list(x))
## <environment: 0x000001e94f08ecf0>
## attr(,"call")
## AICc(x)
## attr(,"class")
## [1] "function" "rankFunction"
## attr(,"beta")
## [1] "none"

summary(top_mods_nonhatch[[1]]) #only one model

## Family: binomial ( logit )
## Formula: cbind(nonhatched, eggs - nonhatched) ~ Day_c + Temperature +
## (1 | unit) + Day_c:Temperature
## Data: development
##
## AIC BIC logLik -2*log(L) df.resid
## 286.1 296.0 -138.0 276.1 49
##
## Random effects:
##
## Conditional model:
## Groups Name Variance Std.Dev.
## unit (Intercept) 0.421 0.6489
## Number of obs: 54, groups: unit, 10
##
## Conditional model:
## Estimate Std. Error z value Pr(>|z|)
## (Intercept) -2.1389 0.3586 -5.965 2.45e-09 ***
## Day_c -0.3505 0.1136 -3.086 0.00203 **
## Temperature20 2.5548 0.4765 5.362 8.24e-08 ***
## Day_c:Temperature20 0.6945 0.1300 5.341 9.26e-08 ***
## ---
## Signif. codes: 0 '***' 0.001 '**' 0.01 '*' 0.05 '.' 0.1 ' ' 1

#avg_mod_nonhatch <- model.avg(top_mods_nonhatch)
#summary(avg_mod_nonhatch)

## 5.3. Diagnostics final model

mod_nonhatched_final <- glmmTMB(
 cbind(nonhatched, eggs - nonhatched) ~
 Temperature * Day_c +
 (1 | unit),
 family = binomial(link = "logit"),
 data = development
)

summary(mod_nonhatched_final)

## Family: binomial ( logit )
## Formula: cbind(nonhatched, eggs - nonhatched) ~ Temperature * Day_c +
## (1 | unit)
## Data: development
##
## AIC BIC logLik -2*log(L) df.resid
## 286.1 296.0 -138.0 276.1 49
##
## Random effects:
##
## Conditional model:
## Groups Name Variance Std.Dev.
## unit (Intercept) 0.421 0.6489
## Number of obs: 54, groups: unit, 10
##
## Conditional model:
## Estimate Std. Error z value Pr(>|z|)
## (Intercept) -2.1389 0.3586 -5.965 2.45e-09 ***
## Temperature20 2.5548 0.4765 5.362 8.24e-08 ***
## Day_c -0.3505 0.1136 -3.086 0.00203 **
## Temperature20:Day_c 0.6945 0.1300 5.341 9.26e-08 ***
## ---
## Signif. codes: 0 '***' 0.001 '**' 0.01 '*' 0.05 '.' 0.1 ' ' 1

AIC(mod_nonhatched_final)

## [1] 286.0665

MuMIn::AICc(mod_nonhatched_final)

## [1] 287.3165

performance::r2(mod_nonhatched_final) # Marginal and conditional R2

## # R2 for Mixed Models
##
## Conditional R2: 0.919
## Marginal R2: 0.781

res_nh <- DHARMa::simulateResiduals(mod_nonhatched_final, plot = FALSE)
plot(res_nh) # simulated residual diagnostics


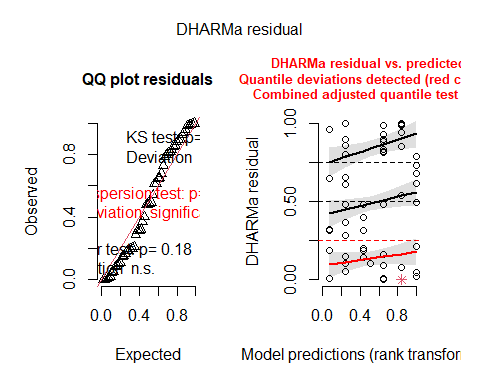


testDispersion(res_nh) # dispersion test


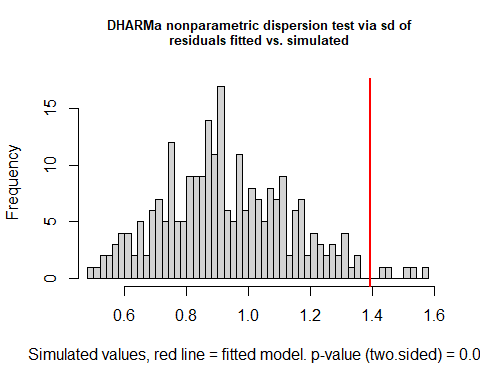


##
## DHARMa nonparametric dispersion test via sd of residuals fitted vs.
## simulated
##
## data: simulationOutput
## dispersion = 1.4981, p-value = 0.04
## alternative hypothesis: two.sided

testZeroInflation(res_nh) # zero-inflation test (less critical in binomial, but informative)


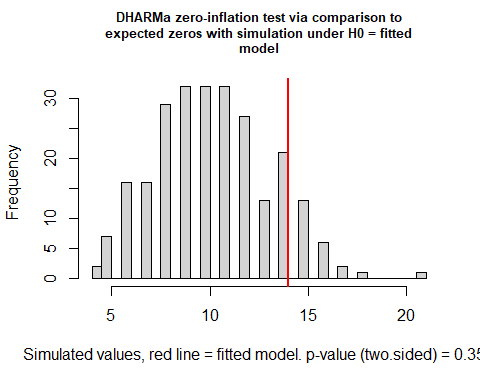


##
## DHARMa zero-inflation test via comparison to expected zeros with
## simulation under H0 = fitted model
##
## data: simulationOutput
## ratioObsSim = 1.3493, p-value = 0.352
## alternative hypothesis: two.sided

testUniformity(res_nh) # KS test for uniformity


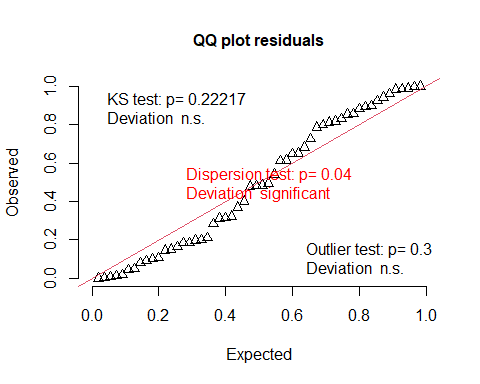


##
## Exact one-sample Kolmogorov-Smirnov test
##
## data: simulationOutput$scaledResiduals
## D = 0.13952, p-value = 0.2222
## alternative hypothesis: two-sided

## 5.4. Betabinomial GLMM:

mod_nh_bb <- glmmTMB(
 cbind(nonhatched, eggs - nonhatched) ~
 Temperature * Day_c +
 (1 | unit),
 family = betabinomial(link = "logit"),
 data = development
)
summary(mod_nh_bb)

## Family: betabinomial ( logit )
## Formula: cbind(nonhatched, eggs - nonhatched) ~ Temperature * Day_c +
## (1 | unit)
## Data: development
##
## AIC BIC logLik -2*log(L) df.resid
## 232.5 244.4 -110.2 220.5 48
##
## Random effects:
##
## Conditional model:
## Groups Name Variance Std.Dev.
## unit (Intercept) 0.05823 0.2413
## Number of obs: 54, groups: unit, 10
##
## Dispersion parameter for betabinomial family (): 3.48
##
## Conditional model:
## Estimate Std. Error z value Pr(>|z|)
## (Intercept) -1.7660 0.3178 -5.556 2.76e-08 ***
## Temperature20 2.1403 0.4084 5.240 1.60e-07 ***
## Day_c -0.1880 0.1321 -1.423 0.1548
## Temperature20:Day_c 0.4824 0.1631 2.958 0.0031 **
## ---
## Signif. codes: 0 '***' 0.001 '**' 0.01 '*' 0.05 '.' 0.1 ' ' 1

performance::r2(mod_nh_bb)

## # R2 for Mixed Models
##
## Conditional R2: 0.812
## Marginal R2: 0.782

##5.5. Diagnostics final model

res_bb <- DHARMa::simulateResiduals(mod_nh_bb, plot = FALSE)
plot(res_bb) # simulated residual diagnostics


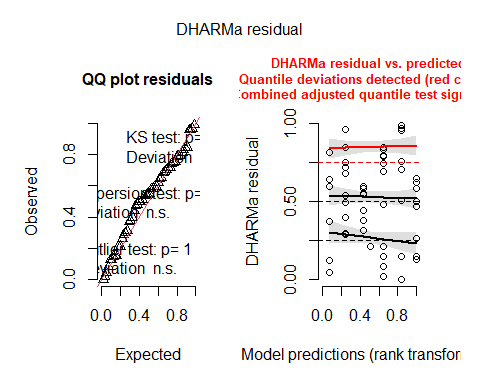


testDispersion(res_bb) # dispersion test


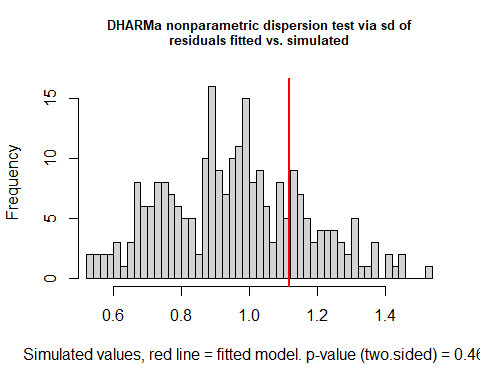


##
## DHARMa nonparametric dispersion test via sd of residuals fitted vs.
## simulated
##
## data: simulationOutput
## dispersion = 1.1693, p-value = 0.464
## alternative hypothesis: two.sided

testZeroInflation(res_bb) # zero-inflation test (less critical in binomial, but informative)


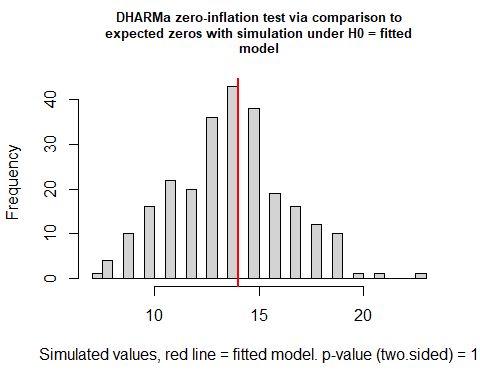


##
## DHARMa zero-inflation test via comparison to expected zeros with
## simulation under H0 = fitted model
##
## data: simulationOutput
## ratioObsSim = 1.0124, p-value = 1
## alternative hypothesis: two.sided

testUniformity(res_bb)


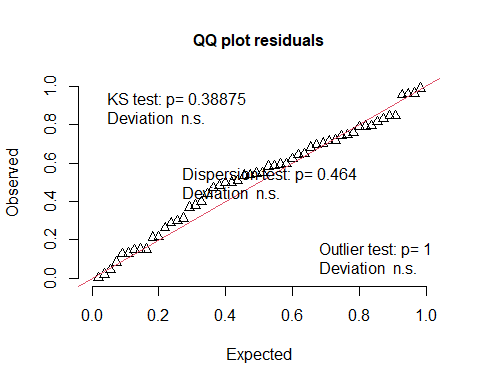


##
## Exact one-sample Kolmogorov-Smirnov test
##
## data: simulationOutput$scaledResiduals
## D = 0.11988, p-value = 0.3888
## alternative hypothesis: two-sided

performance::r2(mod_nh_bb) # Marginal and conditional R2

## # R2 for Mixed Models
##
## Conditional R2: 0.812
## Marginal R2: 0.782

# KS test for uniformity

# 5.5. GLMM Binomial for non-developed

hist(development$nondeveloped,
 col = "grey",
 main = "Non developed distribution",
 xlab = "nondeveloped")


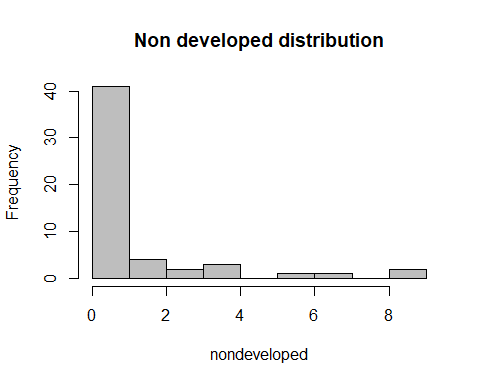


mod_nondeveloped_RE <- glmmTMB(
 cbind(nondeveloped, eggs - nondeveloped) ~
 Treatment * Temperature * Day_c +
 (1 | unit),
 family = binomial(link = "logit"),
 data = development
)

summary(mod_nondeveloped_RE)

## Family: binomial ( logit )
## Formula:
## cbind(nondeveloped, eggs - nondeveloped) ~ Treatment * Temperature *
## Day_c + (1 | unit)
## Data: development
##
## AIC BIC logLik -2*log(L) df.resid
## 242.8 260.7 -112.4 224.8 45
##
## Random effects:
##
## Conditional model:
## Groups Name Variance Std.Dev.
## unit (Intercept) 1.126 1.061
## Number of obs: 54, groups: unit, 10
##
## Conditional model:
## Estimate Std. Error z value Pr(>|z|)
## (Intercept) -3.6497801 0.8044349 -4.537 5.7e-06 ***
## Treatment6PPD-Q 0.6939568 1.1714546 0.592 0.55359
## Temperature20 1.2010291 1.0343589 1.161 0.24559
## Day_c 0.0599287 0.2134153 0.281 0.77886
## Treatment6PPD-Q:Temperature20 0.3357476 1.5979918 0.210 0.83358
## Treatment6PPD-Q:Day_c -0.8494312 0.4415006 -1.924 0.05436 .
## Temperature20:Day_c -0.0004855 0.2241482 -0.002 0.99827
## Treatment6PPD-Q:Temperature20:Day_c 1.2862155 0.4980670 2.582 0.00981 **
## ---
## Signif. codes: 0 '***' 0.001 '**' 0.01 '*' 0.05 '.' 0.1 ' ' 1

library(MuMIn)

options(na.action = "na.fail")
dd <- dredge(mod_nondeveloped_RE, rank = "AICc")
dd

## Global model call: glmmTMB(formula = cbind(nondeveloped, eggs - nondeveloped) ~
## Treatment * Temperature * Day_c + (1 | unit), data = development,
## family = binomial(link = "logit"), ziformula = ~0, dispformula = ~1)
## ---
## Model selection table
## cnd((Int)) dsp((Int)) cnd(Day_c) cnd(Tmp) cnd(Trt) cnd(Day_c:Tmp)
## 3 -3.189 + +
## 12 -3.185 + -0.24740 + +
## 128 -3.650 + 0.05993 + + +
## 16 -3.446 + -0.25970 + + +
## 7 -3.342 + + +
## 4 -3.218 + 0.02671 +
## 1 -2.652 +
## 8 -3.395 + 0.03219 + +
## 5 -2.853 + +
## 32 -3.479 + -0.31080 + + +
## 2 -2.674 + 0.03621
## 39 -3.396 + + +
## 48 -3.488 + -0.26140 + + +
## 6 -2.897 + 0.03957 +
## 24 -3.390 + 0.03653 + +
## 40 -3.424 + 0.03081 + +
## 64 -3.507 + -0.31200 + + +
## 22 -2.897 + 0.05501 +
## 56 -3.428 + 0.03605 + +
## cnd(Day_c:Trt) cnd(Tmp:Trt) cnd(Day_c:Tmp:Trt) df logLik AICc delta
## 3 3 -119.909 246.3 0.00
## 12 5 -117.721 246.7 0.39
## 128 + + + 9 -112.415 246.9 0.62
## 16 6 -117.141 248.1 1.77
## 7 4 -119.631 248.1 1.78
## 4 4 -119.812 248.4 2.14
## 1 2 -122.138 248.5 2.21
## 8 5 -119.491 250.2 3.93
## 5 3 -121.877 250.2 3.94
## 32 + 7 -116.950 250.3 4.04
## 2 3 -121.966 250.4 4.11
## 39 + 5 -119.607 250.5 4.17
## 48 + 7 -117.130 250.7 4.40
## 6 4 -121.672 252.2 5.86
## 24 + 6 -119.483 252.8 6.46
## 40 + 6 -119.484 252.8 6.46
## 64 + + 8 -116.945 253.1 6.79
## 22 + 5 -121.529 254.3 8.01
## 56 + + 7 -119.470 255.4 9.08
## weight
## 3 0.199
## 12 0.164
## 128 0.146
## 16 0.082
## 7 0.082
## 4 0.068
## 1 0.066
## 8 0.028
## 5 0.028
## 32 0.026
## 2 0.025
## 39 0.025
## 48 0.022
## 6 0.011
## 24 0.008
## 40 0.008
## 64 0.007
## 22 0.004
## 56 0.002
## Models ranked by AICc(x)
## Random terms (all models):
## cond(1 | unit)

top_mods_nondev <- get.models(dd, subset = delta <= 2)
top_mods_nondev

## $`3`
## Formula:
## cbind(nondeveloped, eggs - nondeveloped) ~ Temperature + (1 | unit)
## Data: development
## AIC BIC logLik -2*log(L) df.resid
## 245.8175 251.7844 -119.9087 239.8175 51
## Random-effects (co)variances:
##
## Conditional model:
## Groups Name Std.Dev.
## unit (Intercept) 0.4973
##
## Number of obs: 54 / Conditional model: unit, 10
##
## Fixed Effects:
##
## Conditional model:
## (Intercept) Temperature20
## -3.189 1.172
##
## $`12`
## Formula:
## cbind(nondeveloped, eggs - nondeveloped) ~ Day_c + Temperature +
## (1 | unit) + Day_c:Temperature
## Data: development
## AIC BIC logLik -2*log(L) df.resid
## 245.4425 255.3874 -117.7212 235.4425 49
## Random-effects (co)variances:
##
## Conditional model:
## Groups Name Std.Dev.
## unit (Intercept) 0.6851
##
## Number of obs: 54 / Conditional model: unit, 10
##
## Fixed Effects:
##
## Conditional model:
## (Intercept) Day_c Temperature20
## -3.1854 -0.2474 1.1191
## Day_c:Temperature20
## 0.3368
##
## $`128`
## Formula:
## cbind(nondeveloped, eggs - nondeveloped) ~ Day_c + Temperature +
## Treatment + (1 | unit) + Day_c:Temperature + Day_c:Treatment +
## Temperature:Treatment + Day_c:Temperature:Treatment
## Data: development
## AIC BIC logLik -2*log(L) df.resid
## 242.8309 260.7317 -112.4154 224.8309 45
## Random-effects (co)variances:
##
## Conditional model:
## Groups Name Std.Dev.
## unit (Intercept) 1.061
##
## Number of obs: 54 / Conditional model: unit, 10
##
## Fixed Effects:
##
## Conditional model:
## (Intercept) Day_c
## -3.6497801 0.0599287
## Temperature20 Treatment6PPD-Q
## 1.2010291 0.6939568
## Day_c:Temperature20 Day_c:Treatment6PPD-Q
## -0.0004855 -0.8494312
## Temperature20:Treatment6PPD-Q Day_c:Temperature20:Treatment6PPD-Q
## 0.3357476 1.2862155
##
## $`16`
## Formula:
## cbind(nondeveloped, eggs - nondeveloped) ~ Day_c + Temperature +
## Treatment + (1 | unit) + Day_c:Temperature
## Data: development
## AIC BIC logLik -2*log(L) df.resid
## 246.2826 258.2165 -117.1413 234.2826 48
## Random-effects (co)variances:
##
## Conditional model:
## Groups Name Std.Dev.
## unit (Intercept) 0.6691
##
## Number of obs: 54 / Conditional model: unit, 10
##
## Fixed Effects:
##
## Conditional model:
## (Intercept) Day_c Temperature20
## -3.4460 -0.2597 1.1489
## Treatment6PPD-Q Day_c:Temperature20
## 0.5964 0.3573
##
## $`7`
## Formula:
## cbind(nondeveloped, eggs - nondeveloped) ~ Temperature + Treatment +
## (1 | unit)
## Data: development
## AIC BIC logLik -2*log(L) df.resid
## 247.2617 255.2177 -119.6309 239.2617 50
## Random-effects (co)variances:
##
## Conditional model:
## Groups Name Std.Dev.
## unit (Intercept) 0.5087
##
## Number of obs: 54 / Conditional model: unit, 10
##
## Fixed Effects:
##
## Conditional model:
## (Intercept) Temperature20 Treatment6PPD-Q
## -3.3420 1.1891 0.3428
##
## attr(,"rank")
## function (x)
## do.call("rank", list(x))
## <environment: 0x000001e94f462bd0>
## attr(,"call")
## AICc(x)
## attr(,"class")
## [1] "function" "rankFunction"
## attr(,"beta")
## [1] "none"

summary(top_mods_nondev[[1]])

## Family: binomial ( logit )
## Formula:
## cbind(nondeveloped, eggs - nondeveloped) ~ Temperature + (1 | unit)
## Data: development
##
## AIC BIC logLik -2*log(L) df.resid
## 245.8 251.8 -119.9 239.8 51
##
## Random effects:
##
## Conditional model:
## Groups Name Variance Std.Dev.
## unit (Intercept) 0.2473 0.4973
## Number of obs: 54, groups: unit, 10
##
## Conditional model:
## Estimate Std. Error z value Pr(>|z|)
## (Intercept) -3.1891 0.3729 -8.553 <2e-16 ***
## Temperature20 1.1716 0.4592 2.551 0.0107 *
## ---
## Signif. codes: 0 '***' 0.001 '**' 0.01 '*' 0.05 '.' 0.1 ' ' 1

avg_mod_nondev <- model.avg(top_mods_nondev)
summary(avg_mod_nondev)

##
## Call:
## model.avg(object = top_mods_nondev)
##
## Component model call:
## glmmTMB(formula = cbind(nondeveloped, eggs - nondeveloped) ~ <5 unique
## rhs>, data = development, family = binomial(link = "logit"), ziformula
## = ~0, dispformula = ~1)
##
## Component models:
## df logLik AICc delta weight
## 2 3 -119.91 246.30 0.00 0.30
## 124 5 -117.72 246.69 0.39 0.24
## 1234567 9 -112.42 246.92 0.62 0.22
## 1234 6 -117.14 248.07 1.77 0.12
## 23 4 -119.63 248.08 1.78 0.12
##
## Term codes:
## cond(Day_c) cond(Temperature)
## 1 2
## cond(Treatment) cond(Day_c:Temperature)
## 3 4
## cond(Day_c:Treatment) cond(Temperature:Treatment)
## 5 6
## cond(Day_c:Temperature:Treatment)
## 7
##
## Model-averaged coefficients:
## (full average)
## Estimate Std. Error Adjusted SE
## cond((Int)) -3.33805 0.56608 0.57938
## cond(Temperature20) 1.16455 0.65867 0.67604
## cond(Day_c) -0.07888 0.19030 0.19291
## cond(Day_c:Temperature20) 0.12543 0.22178 0.22440
## cond(Treatment6PPD-Q) 0.26499 0.67621 0.69095
## cond(Day_c:Treatment6PPD-Q) -0.18418 0.40594 0.40885
## cond(Temperature20:Treatment6PPD-Q) 0.07280 0.75685 0.77706
## cond(Day_c:Temperature20:Treatment6PPD-Q) 0.27888 0.57854 0.58114
## z value Pr(>|z|)
## cond((Int)) 5.761 <2e-16 ***
## cond(Temperature20) 1.723 0.085 .
## cond(Day_c) 0.409 0.683
## cond(Day_c:Temperature20) 0.559 0.576
## cond(Treatment6PPD-Q) 0.384 0.701
## cond(Day_c:Treatment6PPD-Q) 0.450 0.652
## cond(Temperature20:Treatment6PPD-Q) 0.094 0.925
## cond(Day_c:Temperature20:Treatment6PPD-Q) 0.480 0.631
##
## (conditional average)
## Estimate Std. Error Adjusted SE
## cond((Int)) -3.3380 0.5661 0.5794
## cond(Temperature20) 1.1645 0.6587 0.6760
## cond(Day_c) -0.1355 0.2335 0.2372
## cond(Day_c:Temperature20) 0.2155 0.2551 0.2590
## cond(Treatment6PPD-Q) 0.5754 0.9023 0.9263
## cond(Day_c:Treatment6PPD-Q) -0.8494 0.4415 0.4537
## cond(Temperature20:Treatment6PPD-Q) 0.3357 1.5980 1.6421
## cond(Day_c:Temperature20:Treatment6PPD-Q) 1.2862 0.4981 0.5118
## z value Pr(>|z|)
## cond((Int)) 5.761 <2e-16 ***
## cond(Temperature20) 1.723 0.0850 .
## cond(Day_c) 0.571 0.5678
## cond(Day_c:Temperature20) 0.832 0.4055
## cond(Treatment6PPD-Q) 0.621 0.5345
## cond(Day_c:Treatment6PPD-Q) 1.872 0.0612 .
## cond(Temperature20:Treatment6PPD-Q) 0.204 0.8380
## cond(Day_c:Temperature20:Treatment6PPD-Q) 2.513 0.0120 *
## ---
## Signif. codes: 0 '***' 0.001 '**' 0.01 '*' 0.05 '.' 0.1 ' ' 1

## Diagnostics final model for non-developed

mod_nondeveloped_final <- glmmTMB(
 cbind(nondeveloped, eggs - nondeveloped) ~
 Treatment*Temperature * Day_c +
 (1 | unit),
 family = binomial(link = "logit"),
 data = development
)

summary(mod_nondeveloped_final)

## Family: binomial ( logit )
## Formula:
## cbind(nondeveloped, eggs - nondeveloped) ~ Treatment * Temperature *
## Day_c + (1 | unit)
## Data: development
##
## AIC BIC logLik -2*log(L) df.resid
## 242.8 260.7 -112.4 224.8 45
##
## Random effects:
##
## Conditional model:
## Groups Name Variance Std.Dev.
## unit (Intercept) 1.126 1.061
## Number of obs: 54, groups: unit, 10
##
## Conditional model:
## Estimate Std. Error z value Pr(>|z|)
## (Intercept) -3.6497801 0.8044349 -4.537 5.7e-06 ***
## Treatment6PPD-Q 0.6939568 1.1714546 0.592 0.55359
## Temperature20 1.2010291 1.0343589 1.161 0.24559
## Day_c 0.0599287 0.2134153 0.281 0.77886
## Treatment6PPD-Q:Temperature20 0.3357476 1.5979918 0.210 0.83358
## Treatment6PPD-Q:Day_c -0.8494312 0.4415006 -1.924 0.05436 .
## Temperature20:Day_c -0.0004855 0.2241482 -0.002 0.99827
## Treatment6PPD-Q:Temperature20:Day_c 1.2862155 0.4980670 2.582 0.00981 **
## ---
## Signif. codes: 0 '***' 0.001 '**' 0.01 '*' 0.05 '.' 0.1 ' ' 1

AIC(mod_nondeveloped_final)

## [1] 242.8309

MuMIn::AICc(mod_nondeveloped_final)

## [1] 246.9218

performance::r2(mod_nondeveloped_final) # Marginal and conditional R2

## # R2 for Mixed Models
##
## Conditional R2: 0.900
## Marginal R2: 0.442

res_nondev_RE <- DHARMa::simulateResiduals(mod_nondeveloped_final, plot = FALSE)
plot(res_nondev_RE) # simulated residual diagnostics


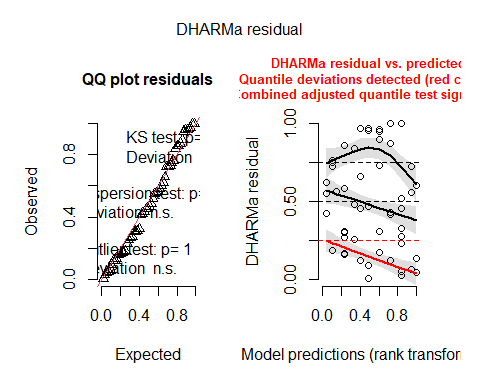


testDispersion(res_nondev_RE) # dispersion test


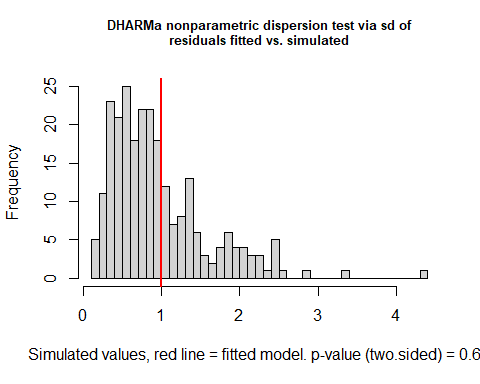


##
## DHARMa nonparametric dispersion test via sd of residuals fitted vs.
## simulated
##
## data: simulationOutput
## dispersion = 1.053, p-value = 0.68
## alternative hypothesis: two.sided

testZeroInflation(res_nondev_RE) # zero-inflation test (less critical in binomial, but informative)


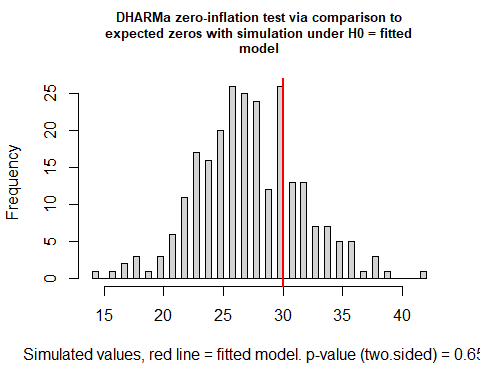


##
## DHARMa zero-inflation test via comparison to expected zeros with
## simulation under H0 = fitted model
##
## data: simulationOutput
## ratioObsSim = 1.0923, p-value = 0.656
## alternative hypothesis: two.sided

testUniformity(res_nondev_RE) # KS test for uniformity


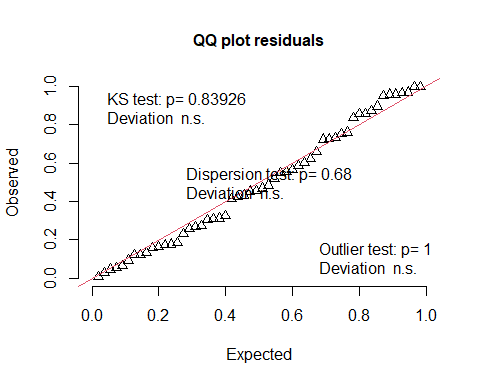


##
## Exact one-sample Kolmogorov-Smirnov test
##
## data: simulationOutput$scaledResiduals
## D = 0.081293, p-value = 0.8393
## alternative hypothesis: two-sided

# # 6. GROWTH

#6.1. Load the data

# Load data
growth <- read.csv("growth.csv", sep = ";", header = TRUE)
summary(growth)

## replicate individual Treatment Temperature
## Min. :1.000 Min. :1.10 Length:96 Min. :15.0
## 1st Qu.:1.000 1st Qu.:1.70 Class :character 1st Qu.:15.0
## Median :2.000 Median :2.50 Mode :character Median :17.5
## Mean :2.021 Mean :2.47 Mean :17.5
## 3rd Qu.:3.000 3rd Qu.:3.20 3rd Qu.:20.0
## Max. :3.000 Max. :3.90 Max. :20.0
## Ancho Largo incrementoLargo DM
## Min. :2.750 Min. :4.800 Min. :-106.87500 Min. : 4.910
## 1st Qu.:3.600 1st Qu.:5.787 1st Qu.: -0.08125 1st Qu.: 8.045
## Median :4.000 Median :6.300 Median : 0.43125 Median :10.325
## Mean :3.904 Mean :6.339 Mean : 33.69590 Mean :10.658
## 3rd Qu.:4.200 3rd Qu.:6.850 3rd Qu.: 0.98125 3rd Qu.:12.675
## Max. :5.000 Max. :8.150 Max. : 228.12500 Max. :19.900
## growtherror growth loggrowth
## Min. :-3.1200 Min. :-3.1200 Min. :-0.05552
## 1st Qu.: 0.0175 1st Qu.: 0.0175 1st Qu.: 0.60384
## Median : 2.3050 Median : 2.3050 Median : 0.79968
## Mean : 2.6326 Mean : 2.6329 Mean : 0.76206
## 3rd Qu.: 4.6450 3rd Qu.: 4.6450 3rd Qu.: 0.93676
## Max. :11.8800 Max. :11.8800 Max. : 1.20085

# Factors & basic cleaning
growth$Treatment <- factor(growth$Treatment, levels = c("CTL","6PPD-Q"))
growth$Temperature <- factor(growth$Temperature) # 15 / 20 as factor
growth$unit <- interaction(growth$Treatment, growth$Temperature, growth$replicate) # random effect for "unit"

summary(growth)

## replicate individual Treatment Temperature Ancho
## Min. :1.000 Min. :1.10 CTL :52 15:48 Min. :2.750
## 1st Qu.:1.000 1st Qu.:1.70 6PPD-Q:44 20:48 1st Qu.:3.600
## Median :2.000 Median :2.50 Median :4.000
## Mean :2.021 Mean :2.47 Mean :3.904
## 3rd Qu.:3.000 3rd Qu.:3.20 3rd Qu.:4.200
## Max. :3.000 Max. :3.90 Max. :5.000
##
## Largo incrementoLargo DM growtherror
## Min. :4.800 Min. :-106.87500 Min. : 4.910 Min. :-3.1200
## 1st Qu.:5.787 1st Qu.: -0.08125 1st Qu.: 8.045 1st Qu.: 0.0175
## Median :6.300 Median : 0.43125 Median :10.325 Median : 2.3050
## Mean :6.339 Mean : 33.69590 Mean :10.658 Mean : 2.6326
## 3rd Qu.:6.850 3rd Qu.: 0.98125 3rd Qu.:12.675 3rd Qu.: 4.6450
## Max. :8.150 Max. : 228.12500 Max. :19.900 Max. :11.8800
##
## growth loggrowth unit
## Min. :-3.1200 Min. :-0.05552 CTL.15.1 :10
## 1st Qu.: 0.0175 1st Qu.: 0.60384 CTL.20.2 :10
## Median : 2.3050 Median : 0.79968 CTL.15.3 :10
## Mean : 2.6329 Mean : 0.76206 6PPD-Q.20.3:10
## 3rd Qu.: 4.6450 3rd Qu.: 0.93676 CTL.20.1 : 9
## Max. :11.8800 Max. : 1.20085 6PPD-Q.15.2: 9
## (Other) :38

#6.2. LMM with random effects

mod_growth_RE <- lmer(
 loggrowth ~ Treatment * Temperature + (1 | unit),
 data = growth,
 REML = FALSE
)
summary(mod_growth_RE)

## Linear mixed model fit by maximum likelihood ['lmerMod']
## Formula: loggrowth ~ Treatment * Temperature + (1 | unit)
## Data: growth
##
## AIC BIC logLik -2*log(L) df.resid
## -4.9 10.5 8.4 -16.9 90
##
## Scaled residuals:
## Min 1Q Median 3Q Max
## -3.4289 -0.8121 0.1269 0.7541 2.0066
##
## Random effects:
## Groups Name Variance Std.Dev.
## unit (Intercept) 0.00000 0.0000
## Residual 0.04911 0.2216
## Number of obs: 96, groups: unit, 12
##
## Fixed effects:
## Estimate Std. Error t value
## (Intercept) 0.70432 0.04188 16.818
## Treatment6PPD-Q 0.05220 0.06488 0.805
## Temperature20 0.21326 0.06164 3.460
## Treatment6PPD-Q:Temperature20 -0.29124 0.09111 -3.197
##
## Correlation of Fixed Effects:
## (Intr) Tr6PPD-Q Tmpr20
## Trtmn6PPD-Q -0.645
## Temperatr20 -0.679 0.439
## T6PPD-Q:T20 0.460 -0.712 -0.677
## optimizer (nloptwrap) convergence code: 0 (OK)
## boundary (singular) fit: see help('isSingular')

library(lmerTest) # carrega DESPRÉS de lme4 per sobreescriure summary()
mod_growth_RE <- lmer(loggrowth ~ Treatment * Temperature + (1 | unit),
 data = growth, REML = FALSE)

summary(mod_growth_RE) # df and p-values (mètode Satterthwaite)

## Linear mixed model fit by maximum likelihood . t-tests use Satterthwaite's
## method [lmerModLmerTest]
## Formula: loggrowth ~ Treatment * Temperature + (1 | unit)
## Data: growth
##
## AIC BIC logLik -2*log(L) df.resid
## -4.9 10.5 8.4 -16.9 90
##
## Scaled residuals:
## Min 1Q Median 3Q Max
## -3.4289 -0.8121 0.1269 0.7541 2.0066
##
## Random effects:
## Groups Name Variance Std.Dev.
## unit (Intercept) 0.00000 0.0000
## Residual 0.04911 0.2216
## Number of obs: 96, groups: unit, 12
##
## Fixed effects:
## Estimate Std. Error df t value Pr(>|t|)
## (Intercept) 0.70432 0.04188 96.00000 16.818 < 2e-16 ***
## Treatment6PPD-Q 0.05220 0.06488 96.00000 0.805 0.423017
## Temperature20 0.21326 0.06164 96.00000 3.460 0.000809 ***
## Treatment6PPD-Q:Temperature20 -0.29124 0.09111 96.00000 -3.197 0.001883 **
## ---
## Signif. codes: 0 '***' 0.001 '**' 0.01 '*' 0.05 '.' 0.1 ' ' 1
##
## Correlation of Fixed Effects:
## (Intr) Tr6PPD-Q Tmpr20
## Trtmn6PPD-Q -0.645
## Temperatr20 -0.679 0.439
## T6PPD-Q:T20 0.460 -0.712 -0.677
## optimizer (nloptwrap) convergence code: 0 (OK)
## boundary (singular) fit: see help('isSingular')

tapply(growth$loggrowth, growth$unit, var)

## CTL.15.1 6PPD-Q.15.1 CTL.20.1 6PPD-Q.20.1 CTL.15.2 6PPD-Q.15.2
## 0.09401846 0.05577102 0.02743148 0.07575639 0.03770507 0.03511140
## CTL.20.2 6PPD-Q.20.2 CTL.15.3 6PPD-Q.15.3 CTL.20.3 6PPD-Q.20.3
## 0.02869096 0.01538129 0.09589280 0.05055987 0.03328593 0.05077096

boxplot(loggrowth ~ unit, data=growth)


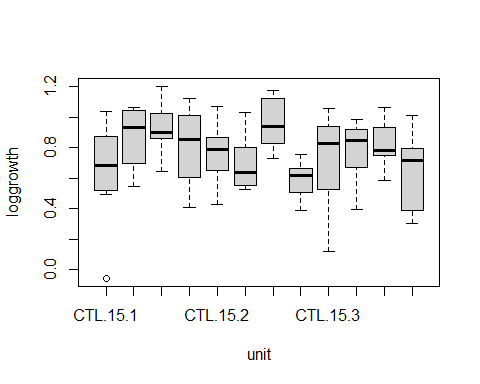


library(lme4); library(performance)

mod_RE_REML <- lmer(loggrowth ~ Treatment * Temperature + (1 | unit),
 data = growth, REML = TRUE)
VarCorr(mod_RE_REML) # mira la variància de (Intercept)|unit

## Groups Name Std.Dev.
## unit (Intercept) 0.00000
## Residual 0.22637

performance::icc(mod_RE_REML) # Intraclass Correlation Coefficient

## [1] NA

isSingular(mod_RE_REML)

## [1] TRUE

agg <- aggregate(loggrowth ~ Treatment + Temperature + unit, data = growth, mean)
lm_agg <- lm(loggrowth ~ Treatment * Temperature, data = agg)
 # prova F clàssica sobre mitjanes per unitat
summary(lm_agg)

##
## Call:
## lm(formula = loggrowth ~ Treatment * Temperature, data = agg)
##
## Residuals:
## Min 1Q Median 3Q Max
## -0.095630 -0.063409 -0.003116 0.048252 0.120093
##
## Coefficients:
## Estimate Std. Error t value Pr(>|t|)
## (Intercept) 0.70835 0.04826 14.676 4.56e-07 ***
## Treatment6PPD-Q 0.07018 0.06826 1.028 0.3339
## Temperature20 0.19381 0.06826 2.839 0.0218 *
## Treatment6PPD-Q:Temperature20 -0.29074 0.09653 -3.012 0.0168 *
## ---
## Signif. codes: 0 '***' 0.001 '**' 0.01 '*' 0.05 '.' 0.1 ' ' 1
##
## Residual standard error: 0.0836 on 8 degrees of freedom
## Multiple R-squared: 0.6099, Adjusted R-squared: 0.4636
## F-statistic: 4.169 on 3 and 8 DF, p-value: 0.04724

#6.3. LM without random effects

mod_growth_noRE <- lm(
 loggrowth ~ Treatment * Temperature,
 data = growth
)
summary(mod_growth_noRE)

##
## Call:
## lm(formula = loggrowth ~ Treatment * Temperature, data = growth)
##
## Residuals:
## Min 1Q Median 3Q Max
## -0.75984 -0.17995 0.02811 0.16711 0.44466
##
## Coefficients:
## Estimate Std. Error t value Pr(>|t|)
## (Intercept) 0.70432 0.04278 16.464 < 2e-16 ***
## Treatment6PPD-Q 0.05220 0.06627 0.788 0.43290
## Temperature20 0.21326 0.06297 3.387 0.00104 **
## Treatment6PPD-Q:Temperature20 -0.29124 0.09307 -3.129 0.00235 **
## ---
## Signif. codes: 0 '***' 0.001 '**' 0.01 '*' 0.05 '.' 0.1 ' ' 1
##
## Residual standard error: 0.2264 on 92 degrees of freedom
## Multiple R-squared: 0.1515, Adjusted R-squared: 0.1239
## F-statistic: 5.476 on 3 and 92 DF, p-value: 0.001656

#compare models

#Likelihood-ratio test (LMM vs LM)
anova(mod_growth_RE, mod_growth_noRE)

## Data: growth
## Models:
## mod_growth_noRE: loggrowth ~ Treatment * Temperature
## mod_growth_RE: loggrowth ~ Treatment * Temperature + (1 | unit)
## npar AIC BIC logLik -2*log(L) Chisq Df Pr(>Chisq)
## mod_growth_noRE 5 -6.8854 5.9363 8.4427 -16.885
## mod_growth_RE 6 -4.8854 10.5007 8.4427 -16.885 0 1 1

# AICc comparison
AICc(mod_growth_RE); AICc(mod_growth_noRE)

## [1] -3.941617

## [1] -6.21877

# Select best by AICc
#mods_growth <- list(RE = mod_growth_RE, noRE = mod_growth_noRE)
#best_growth <- mods_growth[[ which.min(sapply(mods_growth, AICc)) ]]
#best_growth
#summary(best_growth)

#6.4. LMM with random effects model selection

# Dredge over the fixed-effects structure of the LMM
options(na.action = "na.fail")
dredge_growth <- dredge(mod_growth_RE, rank = "AICc")
dredge_growth

## Global model call: lmer(formula = loggrowth ~ Treatment * Temperature + (1 | unit),
## data = growth, REML = FALSE)
## ---
## Model selection table
## (Int) Tmp Trt Tmp:Trt df logLik AICc delta weight
## 8 0.7043 + + + 6 8.443 -3.9 0.00 0.854
## 1 0.7640 3 2.264 1.7 5.67 0.050
## 3 0.8038 + 4 3.149 2.1 6.08 0.041
## 4 0.7694 + + 5 3.888 2.9 6.83 0.028
## 2 0.7321 + 4 2.738 3.0 6.90 0.027
## Models ranked by AICc(x)
## Random terms (all models):
## 1 | unit

# Select models with ΔAICc < 2 (or up to 95% cumulative weight)
#avgset_growth <- subset(dredge_growth, delta <= 2) #only one model
#avgset_growth
#if (nrow(avgset_growth) == 0) {
 # avgset_growth <- dredge_growth[cumsum(dredge_growth$weight) <= 0.95, ]
#}

# Model averaging
#avg_growth <- model.avg(avgset_growth) #only one model
#summary(avg_growth)

# Sum of weights by term
#sw_growth <- sw(avgset_growth)
#sw_growth

#6.5. Diagnostics

summary(mod_growth_RE)

## Linear mixed model fit by maximum likelihood . t-tests use Satterthwaite's
## method [lmerModLmerTest]
## Formula: loggrowth ~ Treatment * Temperature + (1 | unit)
## Data: growth
##
## AIC BIC logLik -2*log(L) df.resid
## -4.9 10.5 8.4 -16.9 90
##
## Scaled residuals:
## Min 1Q Median 3Q Max
## -3.4289 -0.8121 0.1269 0.7541 2.0066
##
## Random effects:
## Groups Name Variance Std.Dev.
## unit (Intercept) 0.00000 0.0000
## Residual 0.04911 0.2216
## Number of obs: 96, groups: unit, 12
##
## Fixed effects:
## Estimate Std. Error df t value Pr(>|t|)
## (Intercept) 0.70432 0.04188 96.00000 16.818 < 2e-16 ***
## Treatment6PPD-Q 0.05220 0.06488 96.00000 0.805 0.423017
## Temperature20 0.21326 0.06164 96.00000 3.460 0.000809 ***
## Treatment6PPD-Q:Temperature20 -0.29124 0.09111 96.00000 -3.197 0.001883 **
## ---
## Signif. codes: 0 '***' 0.001 '**' 0.01 '*' 0.05 '.' 0.1 ' ' 1
##
## Correlation of Fixed Effects:
## (Intr) Tr6PPD-Q Tmpr20
## Trtmn6PPD-Q -0.645
## Temperatr20 -0.679 0.439
## T6PPD-Q:T20 0.460 -0.712 -0.677
## optimizer (nloptwrap) convergence code: 0 (OK)
## boundary (singular) fit: see help('isSingular')

# R2 (marginal / conditional)
performance::r2(mod_growth_RE)

## Random effect variances not available. Returned R2 does not account for random effects.

## # R2 for Mixed Models
##
## Conditional R2: NA
## Marginal R2: 0.153

# Visual diagnostics
check_model(mod_growth_RE)

# DHARMa simulated residuals
res_growth <- DHARMa::simulateResiduals(mod_growth_RE, plot = FALSE)
plot(res_growth)
testDispersion(res_growth)

##
## DHARMa nonparametric dispersion test via sd of residuals fitted vs.
## simulated
##
## data: simulationOutput
## dispersion = 1.0066, p-value = 0.896
## alternative hypothesis: two.sided

testUniformity(res_growth)

##
## Asymptotic one-sample Kolmogorov-Smirnov test
##
## data: simulationOutput$scaledResiduals
## D = 0.077583, p-value = 0.6101
## alternative hypothesis: two-sided

# (Zero-inflation is not relevant for continuous outcomes, but plots can still reveal patterns.)

#normality and homocedasticity (lm equivalent)

lm_equiv_growth <- lm(loggrowth ~ Treatment * Temperature, data = growth)

# Normality of residuals
shapiro.test(residuals(lm_equiv_growth))

##
## Shapiro-Wilk normality test
##
## data: residuals(lm_equiv_growth)
## W = 0.97557, p-value = 0.06975

# Homoscedasticity (Breusch–Pagan)
bptest(lm_equiv_growth)

##
## studentized Breusch-Pagan test
##
## data: lm_equiv_growth
## BP = 4.7951, df = 3, p-value = 0.1874

# #7. CN ratio snails

#7.1. Load the data

# Load data
CN <- read.csv("CN.csv", sep = ";", header = TRUE)

# Factors & basic cleaning
CN$Treatment <- factor(CN$Treatment, levels = c("CTL","6PPD-Q"))
CN$Temperature <- factor(CN$Temperature) # 15 / 20 as factor
CN$unit <- interaction(CN$Treatment, CN$Temperature, CN$replicate) # random effect for "unit"
summary(CN)

## Name replicate Mostra Weight..ug.
## Length:91 Min. :1.000 Length:91 Min. : 281.0
## Class :character 1st Qu.:1.000 Class :character 1st Qu.: 835.0
## Mode :character Median :2.000 Mode :character Median : 950.0
## Mean :2.066 Mean : 916.7
## 3rd Qu.:3.000 3rd Qu.:1077.0
## Max. :3.000 Max. :1172.0
##
## N.. C.. Treatment Temperature CN
## Min. : 6.937 Min. :31.78 CTL :48 15:44 Min. :3.994
## 1st Qu.: 9.015 1st Qu.:40.02 6PPD-Q:43 20:47 1st Qu.:4.321
## Median : 9.283 Median :41.52 Median :4.405
## Mean : 9.391 Mean :41.35 Mean :4.408
## 3rd Qu.: 9.921 3rd Qu.:43.50 3rd Qu.:4.487
## Max. :11.314 Max. :49.10 Max. :4.743
##
## unit
## CTL.15.3 :11
## CTL.20.2 :10
## 6PPD-Q.20.3:10
## 6PPD-Q.15.2: 9
## CTL.20.1 : 8
## CTL.15.1 : 7
## (Other) :36

#7.2. LMM with random effects

mod_CN_RE <- lmer(
 CN ~ Treatment * Temperature + (1 | unit),
 data = CN,
 REML = FALSE
)
summary(mod_CN_RE)

## Linear mixed model fit by maximum likelihood . t-tests use Satterthwaite's
## method [lmerModLmerTest]
## Formula: CN ~ Treatment * Temperature + (1 | unit)
## Data: CN
##
## AIC BIC logLik -2*log(L) df.resid
## -93.3 -78.3 52.7 -105.3 85
##
## Scaled residuals:
## Min 1Q Median 3Q Max
## -2.89135 -0.54044 0.02889 0.53786 2.63201
##
## Random effects:
## Groups Name Variance Std.Dev.
## unit (Intercept) 0.0000 0.0000
## Residual 0.0184 0.1356
## Number of obs: 91, groups: unit, 12
##
## Fixed effects:
## Estimate Std. Error df t value Pr(>|t|)
## (Intercept) 4.40646 0.02713 91.00000 162.424 <2e-16 ***
## Treatment6PPD-Q 0.05391 0.04128 91.00000 1.306 0.195
## Temperature20 -0.01611 0.03919 91.00000 -0.411 0.682
## Treatment6PPD-Q:Temperature20 -0.05785 0.05719 91.00000 -1.011 0.314
## ---
## Signif. codes: 0 '***' 0.001 '**' 0.01 '*' 0.05 '.' 0.1 ' ' 1
##
## Correlation of Fixed Effects:
## (Intr) Tr6PPD-Q Tmpr20
## Trtmn6PPD-Q -0.657
## Temperatr20 -0.692 0.455
## T6PPD-Q:T20 0.474 -0.722 -0.685
## optimizer (nloptwrap) convergence code: 0 (OK)
## boundary (singular) fit: see help('isSingular')

library(lme4); library(performance)

mod_RE_REML <- lmer(CN ~ Treatment * Temperature + (1 | unit),
 data = CN, REML = TRUE)
VarCorr(mod_RE_REML) # Variance (Intercept)|unit

## Groups Name Std.Dev.
## unit (Intercept) 0.01615
## Residual 0.13810

performance::icc(mod_RE_REML) # Intraclass Correlation Coefficient

## # Intraclass Correlation Coefficient
##
## Adjusted ICC: 0.013
## Unadjusted ICC: 0.013

isSingular(mod_RE_REML)

## [1] FALSE

#7.3. LM without random effects

mod_CN_noRE <- lm(
 CN ~ Treatment * Temperature,
 data = CN
)
summary(mod_CN_noRE)

##
## Call:
## lm(formula = CN ~ Treatment * Temperature, data = CN)
##
## Residuals:
## Min 1Q Median 3Q Max
## -0.39220 -0.07331 0.00392 0.07296 0.35702
##
## Coefficients:
## Estimate Std. Error t value Pr(>|t|)
## (Intercept) 4.40646 0.02775 158.815 <2e-16 ***
## Treatment6PPD-Q 0.05391 0.04222 1.277 0.205
## Temperature20 -0.01611 0.04008 -0.402 0.689
## Treatment6PPD-Q:Temperature20 -0.05785 0.05849 -0.989 0.325
## ---
## Signif. codes: 0 '***' 0.001 '**' 0.01 '*' 0.05 '.' 0.1 ' ' 1
##
## Residual standard error: 0.1387 on 87 degrees of freedom
## Multiple R-squared: 0.0404, Adjusted R-squared: 0.007314
## F-statistic: 1.221 on 3 and 87 DF, p-value: 0.307

#compare models

#Likelihood-ratio test (LMM vs LM)
anova(mod_CN_RE, mod_CN_noRE)

## Data: CN
## Models:
## mod_CN_noRE: CN ~ Treatment * Temperature
## mod_CN_RE: CN ~ Treatment * Temperature + (1 | unit)
## npar AIC BIC logLik -2*log(L) Chisq Df Pr(>Chisq)
## mod_CN_noRE 5 -95.335 -82.781 52.668 -105.33
## mod_CN_RE 6 -93.335 -78.270 52.668 -105.33 0 1 1

# AICc comparison
AICc(mod_CN_RE); AICc(mod_CN_noRE)

## [1] -92.3351

## [1] -94.62922

# Select best by AICc
mods_CN <- list(RE = mod_CN_RE, noRE = mod_CN_noRE)
best_CN <- mods_CN[[ which.min(sapply(mods_CN, AICc)) ]]
best_CN

##
## Call:
## lm(formula = CN ~ Treatment * Temperature, data = CN)
##
## Coefficients:
## (Intercept) Treatment6PPD-Q
## 4.40646 0.05391
## Temperature20 Treatment6PPD-Q:Temperature20
## -0.01611 -0.05785

summary(best_CN)

##
## Call:
## lm(formula = CN ~ Treatment * Temperature, data = CN)
##
## Residuals:
## Min 1Q Median 3Q Max
## -0.39220 -0.07331 0.00392 0.07296 0.35702
##
## Coefficients:
## Estimate Std. Error t value Pr(>|t|)
## (Intercept) 4.40646 0.02775 158.815 <2e-16 ***
## Treatment6PPD-Q 0.05391 0.04222 1.277 0.205
## Temperature20 -0.01611 0.04008 -0.402 0.689
## Treatment6PPD-Q:Temperature20 -0.05785 0.05849 -0.989 0.325
## ---
## Signif. codes: 0 '***' 0.001 '**' 0.01 '*' 0.05 '.' 0.1 ' ' 1
##
## Residual standard error: 0.1387 on 87 degrees of freedom
## Multiple R-squared: 0.0404, Adjusted R-squared: 0.007314
## F-statistic: 1.221 on 3 and 87 DF, p-value: 0.307

#7.4. LMM with random effects model selection

# Dredge over the fixed-effects structure of the LMM
options(na.action = "na.fail")
dredge_CN <- dredge(mod_CN_RE, rank = "AICc")
dredge_CN

## Global model call: lmer(formula = CN ~ Treatment * Temperature + (1 | unit), data = CN,
## REML = FALSE)
## ---
## Model selection table
## (Int) Tmp Trt Tmp:Trt df logLik AICc delta weight
## 1 4.408 3 50.791 -95.3 0.00 0.331
## 2 4.430 + 4 51.818 -95.2 0.14 0.310
## 4 4.419 + + 5 52.159 -93.6 1.69 0.142
## 3 4.399 + 4 51.037 -93.6 1.70 0.142
## 8 4.406 + + + 6 52.668 -92.3 2.97 0.075
## Models ranked by AICc(x)
## Random terms (all models):
## 1 | unit

# Select models with ΔAICc < 2 (or up to 95% cumulative weight)
avgset_CN <- subset(dredge_CN, delta < 2)
#if (nrow(avgset_CN) == 0) {
 avgset_CN <- dredge_CN[cumsum(dredge_CN$weight) <= 0.95, ]
#}

# Model averaging
avg_CN <- model.avg(avgset_CN)
summary(avg_CN)

##
## Call:
## model.avg(object = avgset_CN)
##
## Component model call:
## lmer(formula = CN ~ <4 unique rhs>, data = CN, REML = FALSE)
##
## Component models:
## df logLik AICc delta weight
## (Null) 3 50.79 -95.31 0.00 0.36
## 1 4 51.82 -95.17 0.14 0.33
## 12 5 52.16 -93.61 1.69 0.15
## 2 4 51.04 -93.61 1.70 0.15
##
## Term codes:
## Temperature Treatment
## 1 2
##
## Model-averaged coefficients:
## (full average)
## Estimate Std. Error Adjusted SE z value Pr(>|z|)
## (Intercept) 4.41575 0.02234 0.02258 195.594 <2e-16 ***
## Temperature20 -0.02051 0.02905 0.02924 0.701 0.483
## Treatment6PPD-Q 0.00677 0.01898 0.01917 0.353 0.724
##
## (conditional average)
## Estimate Std. Error Adjusted SE z value Pr(>|z|)
## (Intercept) 4.41575 0.02234 0.02258 195.594 <2e-16 ***
## Temperature20 -0.04199 0.02873 0.02914 1.441 0.150
## Treatment6PPD-Q 0.02206 0.02892 0.02932 0.752 0.452
## ---
## Signif. codes: 0 '***' 0.001 '**' 0.01 '*' 0.05 '.' 0.1 ' ' 1

# Sum of weights by term
sw_CN <- sw(avgset_CN)
sw_CN

## Temperature Treatment
## Sum of weights: 0.49 0.31
## N containing models: 2 2

#7.5. Diagnostics of the final model

# R2 (marginal / conditional)
mod_CNavg_RE <- lmer(CN ~ Treatment + Temperature + (1 | unit),
 data = CN, REML = TRUE)
summary(mod_CNavg_RE)

## Linear mixed model fit by REML. t-tests use Satterthwaite's method [
## lmerModLmerTest]
## Formula: CN ~ Treatment + Temperature + (1 | unit)
## Data: CN
##
## REML criterion at convergence: -87.2
##
## Scaled residuals:
## Min 1Q Median 3Q Max
## -2.9277 -0.6165 0.0174 0.5944 2.4771
##
## Random effects:
## Groups Name Variance Std.Dev.
## unit (Intercept) 5.685e-05 0.00754
## Residual 1.920e-02 0.13856
## Number of obs: 91, groups: unit, 12
##
## Fixed effects:
## Estimate Std. Error df t value Pr(>|t|)
## (Intercept) 4.41947 0.02470 5.46635 178.916 1.64e-11 ***
## Treatment6PPD-Q 0.02393 0.02953 6.21361 0.811 0.448
## Temperature20 -0.04330 0.02950 6.15929 -1.468 0.191
## ---
## Signif. codes: 0 '***' 0.001 '**' 0.01 '*' 0.05 '.' 0.1 ' ' 1
##
## Correlation of Fixed Effects:
## (Intr) T6PPD-
## Trtmn6PPD-Q -0.518
## Temperatr20 -0.573 -0.078

performance::r2(mod_CNavg_RE)

## # R2 for Mixed Models
##
## Conditional R2: 0.032
## Marginal R2: 0.029

# Visual diagnostics
check_model(mod_CNavg_RE)

# DHARMa simulated residuals
res_CN <- DHARMa::simulateResiduals(mod_CNavg_RE, plot = FALSE)
plot(res_CN)
testDispersion(res_CN)

##
## DHARMa nonparametric dispersion test via sd of residuals fitted vs.
## simulated
##
## data: simulationOutput
## dispersion = 0.97357, p-value = 0.864
## alternative hypothesis: two.sided

testUniformity(res_CN)

##
## Asymptotic one-sample Kolmogorov-Smirnov test
##
## data: simulationOutput$scaledResiduals
## D = 0.06822, p-value = 0.7908
## alternative hypothesis: two-sided

# (Zero-inflation is not relevant for continuous outcomes, but plots can still reveal patterns.)

#normality and homocedasticity (lm equivalent)

lm_equiv_CN <- lm(CN ~ Treatment + Temperature, data = CN)

# Normality of residuals
shapiro.test(residuals(lm_equiv_CN))

##
## Shapiro-Wilk normality test
##
## data: residuals(lm_equiv_CN)
## W = 0.99172, p-value = 0.8445

# Homoscedasticity (Breusch–Pagan)
bptest(lm_equiv_CN)

##
## studentized Breusch-Pagan test
##
## data: lm_equiv_CN
## BP = 1.9281, df = 2, p-value = 0.3814

# 8. Motility

#8.1. Load the data

# Load data
motility <- read.csv("motility.csv", sep = ";", header = TRUE)

# Factors & basic cleaning
motility$Treatment <- factor(motility$Treatment, levels = c("CTL","6PPD-Q"))
motility$Temperature <- factor(motility$Temperature) # 15 / 20 as factor
 # random effect for "unit" not needed (1 measure per replicate)

#8.2. LM without random effects

mod_motility_noRE <- lm(
 velocity ~ Treatment * Temperature,
 data = motility
)
summary(mod_motility_noRE)

##
## Call:
## lm(formula = velocity ~ Treatment * Temperature, data = motility)
##
## Residuals:
## Min 1Q Median 3Q Max
## -0.34489 -0.06280 -0.01506 0.06315 0.40333
##
## Coefficients:
## Estimate Std. Error t value Pr(>|t|)
## (Intercept) 1.1090 0.1417 7.825 5.12e-05 ***
## Treatment6PPD-Q -0.5146 0.2004 -2.567 0.0333 *
## Temperature20 -0.4727 0.2004 -2.358 0.0461 *
## Treatment6PPD-Q:Temperature20 0.4525 0.2835 1.596 0.1491
## ---
## Signif. codes: 0 '***' 0.001 '**' 0.01 '*' 0.05 '.' 0.1 ' ' 1
##
## Residual standard error: 0.2455 on 8 degrees of freedom
## Multiple R-squared: 0.5483, Adjusted R-squared: 0.3789
## F-statistic: 3.237 on 3 and 8 DF, p-value: 0.08175

#8.3. LM model selection

# Dredge over the fixed-effects structure of the LMM
options(na.action = "na.fail")
dredge_motility <- dredge(mod_motility_noRE, rank = "AICc")
dredge_motility

## Global model call: lm(formula = velocity ~ Treatment * Temperature, data = motility)
## ---
## Model selection table
## (Int) Tmp Trt Tmp:Trt df logLik AICc delta weight
## 1 0.7285 2 -2.509 10.4 0.00 0.371
## 3 0.8727 + 3 -0.912 10.8 0.47 0.293
## 2 0.8517 + 3 -1.386 11.8 1.42 0.182
## 4 0.9959 + + 4 0.600 12.5 2.16 0.126
## 8 1.1090 + + + 5 2.259 15.5 5.13 0.029
## Models ranked by AICc(x)

# Select models with ΔAICc < 2 (or up to 95% cumulative weight)
avgset_motility <- subset(dredge_motility, delta <= 2)
#if (nrow(avgset_motility) == 0) {
# avgset_motility <- dredge_motility[cumsum(dredge_motility$weight) <= 0.95, ]}

# Model averaging
avg_motility <- model.avg(avgset_motility)
summary(avg_motility)

##
## Call:
## model.avg(object = avgset_motility)
##
## Component model call:
## lm(formula = velocity ~ <3 unique rhs>, data = motility)
##
## Component models:
## df logLik AICc delta weight
## (Null) 2 -2.51 10.35 0.00 0.44
## 2 3 -0.91 10.82 0.47 0.35
## 1 3 -1.39 11.77 1.42 0.22
##
## Term codes:
## Temperature Treatment
## 1 2
##
## Model-averaged coefficients:
## (full average)
## Estimate Std. Error Adjusted SE z value Pr(>|z|)
## (Intercept) 0.80496 0.12674 0.13892 5.794 <2e-16 ***
## Treatment6PPD-Q -0.09980 0.16809 0.17610 0.567 0.571
## Temperature20 -0.05312 0.12896 0.13598 0.391 0.696
##
## (conditional average)
## Estimate Std. Error Adjusted SE z value Pr(>|z|)
## (Intercept) 0.8050 0.1267 0.1389 5.794 1e-08 ***
## Treatment6PPD-Q -0.2883 0.1651 0.1877 1.536 0.125
## Temperature20 -0.2465 0.1718 0.1953 1.262 0.207
## ---
## Signif. codes: 0 '***' 0.001 '**' 0.01 '*' 0.05 '.' 0.1 ' ' 1

# Sum of weights by term
sw_motility <- sw(avgset_motility)
sw_motility

## Treatment Temperature
## Sum of weights: 0.35 0.22
## N containing models: 1 1

#8.4. Diagnostics

#normality and homocedasticity (lm )

lm_equiv_motility <- lm(velocity ~ Treatment + Temperature, data = motility)
summary(lm_equiv_motility)

##
## Call:
## lm(formula = velocity ~ Treatment + Temperature, data = motility)
##
## Residuals:
## Min 1Q Median 3Q Max
## -0.41508 -0.14109 0.01472 0.11326 0.51645
##
## Coefficients:
## Estimate Std. Error t value Pr(>|t|)
## (Intercept) 0.9959 0.1329 7.495 3.71e-05 ***
## Treatment6PPD-Q -0.2883 0.1534 -1.879 0.0929 .
## Temperature20 -0.2465 0.1534 -1.606 0.1427
## ---
## Signif. codes: 0 '***' 0.001 '**' 0.01 '*' 0.05 '.' 0.1 ' ' 1
##
## Residual standard error: 0.2658 on 9 degrees of freedom
## Multiple R-squared: 0.4044, Adjusted R-squared: 0.2721
## F-statistic: 3.056 on 2 and 9 DF, p-value: 0.09709

# Normality of residuals
shapiro.test(residuals(lm_equiv_motility))

##
## Shapiro-Wilk normality test
##
## data: residuals(lm_equiv_motility)
## W = 0.97162, p-value = 0.927

# Homoscedasticity (Breusch–Pagan)
bptest(lm_equiv_motility)

##
## studentized Breusch-Pagan test
##
## data: lm_equiv_motility
## BP = 2.8721, df = 2, p-value = 0.2379
